# Synthesis and biological evaluation of novel benzothiazole derivatives as potential anticancer and antiinflammatory agents

**DOI:** 10.3389/fchem.2024.1384301

**Published:** 2024-03-18

**Authors:** Xuemei Xu, Zhaojingtao Zhu, Siyu Chen, Yanneng Fu, Jinxia Zhang, Yangyang Guo, Zhouyang Xu, Yingying Xi, Xuebao Wang, Faqing Ye, Huijun Chen, Xiaojiao Yang

**Affiliations:** ^1^ Department of Pharmacy, Wenzhou Hospital of Integrated Traditional Chinese and Western Medicine, Wenzhou, China; ^2^ School of Pharmaceutical Science, Wenzhou Medical University, Wenzhou, China; ^3^ Department of Pharmacy, The First People’s Hospital of Taizhou, Taizhou, China; ^4^ Scientific Research Center, Wenzhou Medical University, Wenzhou, China

**Keywords:** organic synthesis, benzothiazole derivatives, anticancer, antiinflammatory, biological evaluation

## Abstract

**Introduction:** Cancer, a significant global health concern, necessitates innovative treatments. The pivotal role of chronic inflammation in cancer development underscores the urgency for novel therapeutic strategies. Benzothiazole derivatives exhibit promise due to their distinctive structures and broad spectrum of biological effects. This study aims to explore new anti-tumor small molecule drugs that simultaneously anti-inflammatory and anticancer based on the advantages of benzothiazole frameworks.

**Methods:** The compounds were characterized by nuclear magnetic resonance (NMR), liquid chromatograph-mass spectrometer (LC-MS) and high performance liquid chromatography (HPLC) for structure as well as purity and other related physicochemical properties. The effects of the compounds on the proliferation of human epidermoid carcinoma cell line (A431) and human non-small cell lung cancer cell lines (A549, H1299) were evaluated by MTT method. The effect of compounds on the expression levels of inflammatory factors IL-6 and TNF-α in mouse monocyte macrophages (RAW264.7) was assessed using enzyme-linked immunosorbent assay (ELISA). The effect of compounds on apoptosis and cell cycle of A431 and A549 cells was evaluated by flow cytometry. The effect of compounds on A431 and A549 cell migration was evaluated by scratch wound healing assay. The effect of compounds on protein expression levels in A431 and A549 cells was assessed by Western Blot assay. The physicochemical parameters, pharmacokinetic properties, toxicity and drug similarity of the active compound were predicted using Swiss ADME and admetSAR web servers.

**Results:** Twenty-five novel benzothiazole compounds were designed and synthesized, with their structures confirmed through spectrogram verification. The active compound 6-chloro-*N*-(4-nitrobenzyl) benzo[d] thiazol-2-amine (compound **B7**) was screened through a series of bioactivity assessments, which significantly inhibited the proliferation of A431, A549 and H1299 cancer cells, decreased the activity of IL-6 and TNF-α, and hindered cell migration. In addition, at concentrations of 1, 2, and 4 μM, **B7** exhibited apoptosis-promoting and cell cycle-arresting effects similar to those of the lead compound 7-chloro-*N*-(2, 6-dichlorophenyl) benzo[d] thiazole-2-amine (compound **4i**). Western blot analysis confirmed that **B7** inhibited both AKT and ERK signaling pathways in A431 and A549 cells. The prediction results of ADMET indicated that **B7** had good drug properties.

**Discussion:** This study has innovatively developed a series of benzothiazole derivatives, with a focus on compound **B7** due to its notable dual anticancer and anti-inflammatory activities. **B7** stands out for its ability to significantly reduce cancer cell proliferation in A431, A549, and H1299 cell lines and lower the levels of inflammatory cytokines IL-6 and TNF-α. These results position **B7**B7 as a promising candidate for dual-action cancer therapy. The study’s mechanistic exploration, highlighting **B7**’s simultaneous inhibition of the AKT and ERK pathways, offers a novel strategy for addressing both the survival mechanisms of tumor cells and the inflammatory milieu facilitating cancer progression.

## 1 Introduction

Cancer is a formidable disease that poses a significant threat to human health and has been a persistent challenge in the global public health domain (Ferlay et al., 2015; Fitzmaurice et al., 2015; Bray et al., 2021; Sung et al., 2021). Despite notable advances in the medical field, cancer treatment still confronts various limitations and challenges (Chang et al., 2021; Craig et al., 2021; Ghanbar and Suresh, 2024). Drug therapy stands out as a widely employed approach in cancer treatment (Wu et al., 2022), where the effectiveness and side effects of drugs are pivotal factors influencing treatment outcomes (Dinić et al., 2020; Steinbrueck et al., 2020; Li et al., 2024b). Consequently, the quest for novel anticancer drugs has emerged as a central focus of contemporary research.

Chronic inflammation is increasingly recognized as a critical factor in cancer development. This complex interplay facilitates tumor initiation and progression. IL-6 and TNF-α are key inflammatory cytokines known for their roles in immune regulation, inflammation, and cell proliferation. Their involvement in tumor development and progression is a subject of ongoing research (Hastir et al., 2020; Park et al., 2020; Cowan et al., 2022). These research underscores the multifaceted roles of IL-6 and TNF-α in tumor development. These cytokines not only contribute to the pro-inflammatory tumor microenvironment but also interact with various signaling pathways and immune cells, influencing both local tumor growth and systemic inflammatory responses. Their impact on tumor development and progression highlights their potential as targets for therapeutic intervention in cancer treatment (Meza et al., 2021).

**FIGURE 1:**
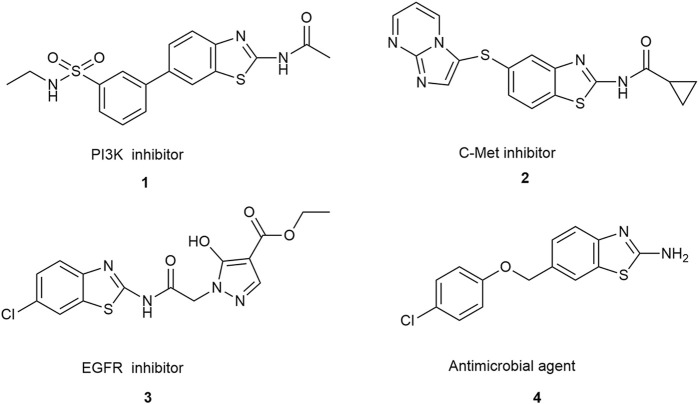
Benzothiazole as anticancer agents and antimicrobial agents.

**SCHEME 1:**
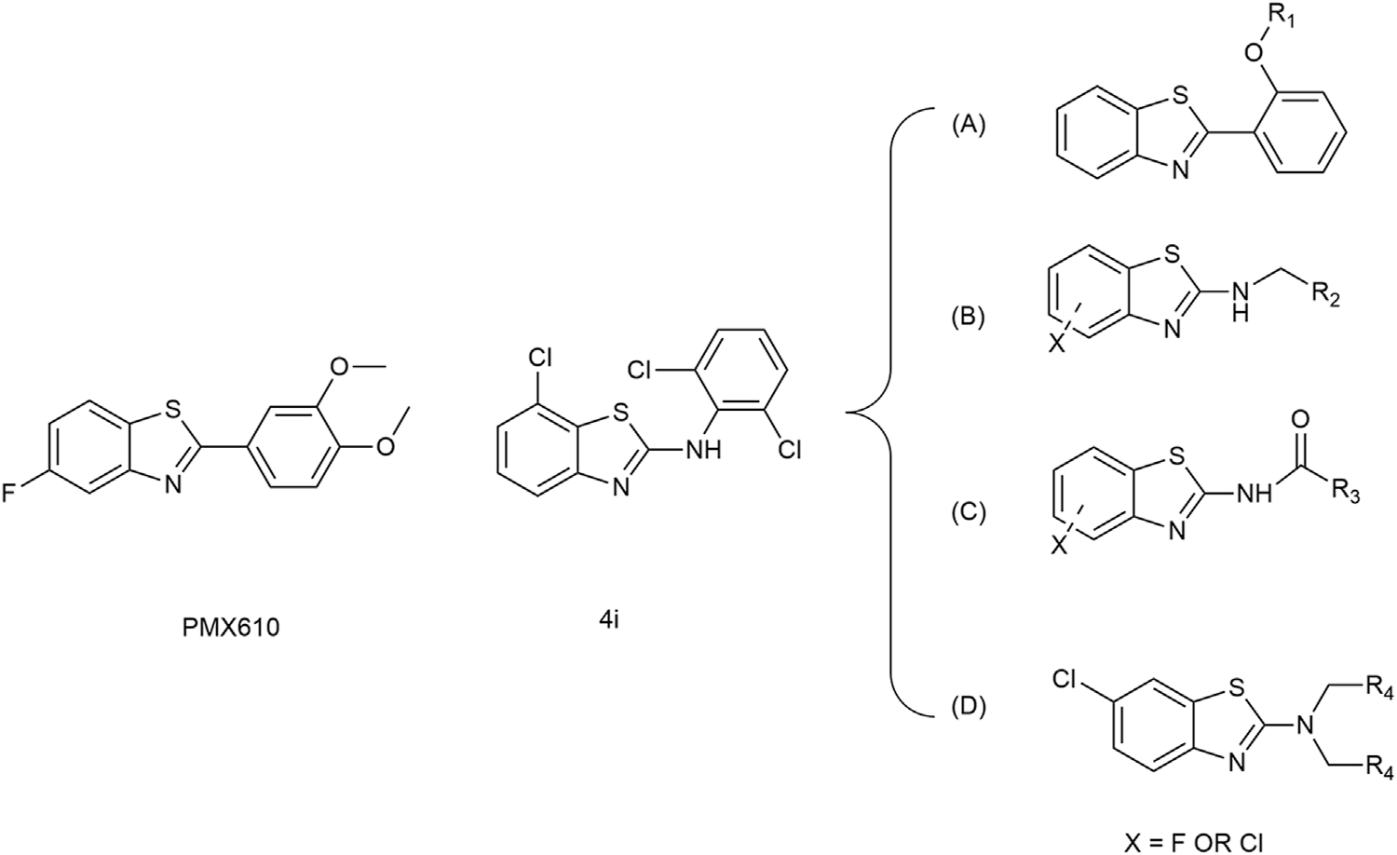
Design principle of target compounds.

Over the past two decades, benzothiazole compounds have attracted considerable research attention due to their distinctive structure and diverse biological activities, including anti-tumor (Kamal et al., 2010; Kamal et al., 2011; El-Helby et al., 2019; Makowska et al., 2019; Mokesch et al., 2020), anti-inflammatory (Lee et al., 2011; Kumar and Singh, 2021), neuroprotective (Choi et al., 2007), antibacterial (Al-Tel et al., 2011; Racané et al., 2020), and antiparasitic (Awadh, 2023) activities, etc. In recent years, extensive research has focused on modifying the benzothiazole nucleus to enhance its anti-tumor activities. Among the modified structures, benzothiazole derivatives exhibiting specificity towards various anti-tumor receptors have consistently emerged. This includes compounds interacting with receptor tyrosine kinases such as C-Met and EGFR (Noolvi et al., 2012), those influencing the PI3K/Akt/mTOR pathway (D'Angelo et al., 2011), and those exhibiting antimicrobial properties, as illustrated in Scheme 1. Additionally, two compounds have garnered special attention for their excellent anti-tumor effects: **PMX610** [2-(3,4-dimethoxyphenyl)-5-fluorobenzothiazole] (Mortimer et al., 2006) and compound **4i** (Noolvi et al., 2012) (Figure 1). **PMX610** has been reported to possess potent and selective *in vitro* anti-tumor properties in human cancer cell lines, particularly against non-small cell lung, colon, and breast cancer lines from 60 human cancer cell line screen. Compound **4i** demonstrated promising anticancer activity against the non-small cell lung cancer cell line HOP-92, with substitutions at the 2,6-positions displayed significant anticancer potential in initial cytotoxicity screening across three human cancer cell lines. These findings underscore the significance of benzothiazole as a core structure in drug synthesis, with modifications to the benzothiazole nucleus enhancing its anticancer activity. The importance of benzothiazole and its derivatives in anticancer research is progressively becoming apparent.

Based on the above analysis, this study is to explore new anti-tumor small molecule drugs that simultaneously anti-inflammatory and anticancer based on the advantages of benzothiazole frameworks. Based on compounds **PMX610** and **4i** as lead compounds, we attempted to design A, B, C, and D four series of compounds. Series A of benzothiazoles has been designed by incorporating diverse substituted phenyl groups at the 2-phenyl position of 2-phenyl benzothiazole, drawing inspiration from existing literature and undergoing further optimization (Kumbhare et al., 2012). Series B and C of benzothiazoles have been meticulously designed by introducing an additional carbon atom or carbonyl groups between two rings, compared to compound **4i**. Series D of benzothiazoles has been designed as a further replacement of Series B (Figure 1). The compounds designed in this study exhibit a distinct departure in structure from classical small molecule anti-tumor compounds. This structural variation enables the exploration of the relationship between activity and structure, with the intention of leading to the derivation of more advantageous molecular architectures. We anticipate that these compounds will emerge as promising candidates for anticancer prospects, providing scientific evidence for future anticancer drug development and introducing new possibilities to clinical treatment.

## 2 Materials and methods

### 2.1 Chemical reagents and instruments

The reagents and alkoxyamine intermediates employed in chemical synthesis were procured directly from reputable suppliers such as Macklin, Aladdin, and Energy Chemical. All solvents utilized possessed high chemical purity and underwent no further treatment. These included petroleum ether (PE), ethyl acetate (EA), dichloromethane (DCM), dimethyl sulfoxide (DMSO), and 1,4-Dioxane. The progression of the reaction was tracked through analytical thin-layer chromatography (TLC) utilizing a silica gel GF254 plate (Qingdao Haiyang Chemical Plant, China), with spot observations made under UV light at 254 nm or 365 nm. Column chromatography was conducted using silica gel (90–150 μm; Qingdao Ocean Chemical Co., Ltd.). The melting points were determined using the XT-4 micromelting point apparatus without correction. ^1^H NMR and ^13^C NMR spectra were acquired using a Bruker 400/600 MHz Avance NMR spectrometer, employing CDCl_3_ or DMSO-*d*_*6*_ as solvents. Mass spectra were generated using an ACQUITY I-Class UPLC and a XEVO TQD triple quadrupole Mass Spectrometer (Waters, USA). HPLC (Agilent 1260, USA) assessed the purity of the compounds, all of which exhibited purity levels surpassing 96%. Elemental analysis for C, H, and N was conducted via an elemental analyzer (Flash EA1112, United States) and found to be within ± 0.3% of the theoretical values.

### 2.2 Synthesis method of 2-phenol-benzothiazole

A substantial quantity of 2-phenol-benzothiazole must be synthesized. Benzothiazole (1 mmol), 2-hydroxyiodobenzene (1 mmol), and K_2_CO_3_ (2 mmol) as a binding agent were combined in 2 mL of DMSO. The resulting mixture is magnetically stirred at 120°C for 6 h, with the reaction progress monitored via TLC. Upon completion, the reaction mixture was cooled to room temperature, and the solvent is evaporated under reduced pressure. Following this, water (8 mL) and an equivalent volume of ethyl acetate were introduced for extraction through multiple iterations of small-volume extractions (3 times). The aqueous layer was discarded, and the organic layer was desiccated with Na_2_SO_4_. Subsequent to drying, the organic layer underwent evaporation under reduced pressure, and the resulting residue was subjected to column chromatography. Intermediate 1 was extracted through column chromatography using a mobile phase of petroleum ether: ethyl acetate = 2:1. The yield is 50%, and the product was dried in an oven for subsequent use.

### 2.3 General synthesis method of A1-A8

To attain the desired A-type final product, 2-phenol-benzothiazole (1 mmol) was combined with various brominated compounds (1.2 mmol) and K_2_CO_3_ (2 mmol) as a binding agent, within of acetonitrile (6 mL). The resulting mixture underwent stirring at room temperature for a duration of 3 h. Throughout the reaction, the solution underwent a noticeable transition from a pale-yellow suspension to a white suspension, with progress monitored through TLC. Following the completion of the reaction, solvent evaporation was conducted under reduced pressure. Subsequently, water (6 mL) was introduced to dissolve K_2_CO_3_, followed by the addition of an equivalent volume of ethyl acetate for extraction. This extraction process was iteratively performed in three steps, employing small aliquots. The aqueous layer was discarded, and the organic layers were consolidated and desiccated using solid Na_2_SO_4_. Following desiccation, the organic layer underwent evaporation under reduced pressure to eliminate excess ethyl acetate. The resultant product was subjected to recrystallization using petroleum ether. Yield determination took place after the drying process, and subsequent characterization of the product was performed.

### 2.4 General synthesis method of B1-B8

In a 100 mL three-necked flask, either 2-amino-6-chlorobenzothiazole (3.4 mmol) or 2-amino-5-fluorobenzothiazole (3.4 mmol), along with 2 mmol of K_2_CO_3_ as a binding agent, were introduced. Subsequently, acetonitrile (6 mL) was added, and the mixture underwent sonication. Concurrently, benzyl bromide (1 mmol) was dissolved in acetonitrile (15 mL). The benzyl bromide solution was then meticulously introduced dropwise into the three-necked flask using a constant pressure dropping funnel, with a controlled rate of 1 drop every 5 s. The ensuing reaction mixture underwent reflux for a duration of 6–7 h, with the progression monitored via TLC. Upon completion, the reaction mixture was gradually cooled to room temperature, and the solvent was then evaporated under reduced pressure. Extraction ensued by introducing saturated NaCl (8 mL) and an equivalent volume of ethyl acetate, followed by multiple iterations for thorough extraction. The resultant organic layer was separated, and solid Na_2_SO_4_ was incorporated for desiccation. The desiccated organic layer underwent further evaporation under reduced pressure, and the resultant residue underwent purification through column chromatography. The elution solvent comprised a blend of petroleum ether and ethyl acetate in a 9:1 ratio. Fractions collected during chromatography were concentrated under reduced pressure, yielding the final solid product. The overall yield was determined post-drying.

### 2.5 General synthesis method of C1-C7

In the reaction involving 2-amino-6-chlorobenzothiazole (1 mmol) or 2-amino-5-fluorobenzothiazole (1 mmol), acyl chloride (4 mmol), and triethylamine (1 mL) as a binding agent, dioxane (10 mL) was employed as the solvent. The reaction mixture underwent reflux for 3–4 h, with TLC used to monitor the reaction progress. After completion, the reaction mixture was cooled to room temperature, and saturated Na_2_CO_3_, in a molar ratio equivalent to the acyl chloride, was added. The resulting mixture was stirred overnight. The precipitated product was filtered, followed by drying. The yield was determined post-drying.

### 2.6 General synthesis method of D1-D2

In a 100 mL three-necked flask, either **B6** (3.4 mmol) or **B8** (3.4 mmol), along with K_2_CO_3_ (2 mmol) as a binding agent, was introduced. Following this, acetonitrile (6 mL) was added, and the resulting mixture underwent sonication. Concurrently, benzyl bromide (1 mmol) was dissolved in acetonitrile (15 mL). The benzyl bromide solution was subsequently added dropwise to the three-necked flask using a constant-pressure dropping funnel at a rate of 1 drop every 5 s. The reaction mixture underwent reflux for 6–7 h, with TLC monitoring the reaction progress. Upon completion, the reaction mixture was cooled to room temperature, and the solvent was evaporated under reduced pressure. Extraction was carried out by adding saturated NaCl (8 mL) and an equal volume of ethyl acetate in multiple iterations. The organic layer was separated, and solid Na_2_SO_4_ was added for drying. The dried organic layer was evaporated under reduced pressure, and the resulting residue underwent purification via column chromatography. The elution solvent, a mixture of petroleum ether and ethyl acetate in a 15:1 ratio, was employed. The collected fractions were concentrated under reduced pressure to obtain the solid product, with yield determination following the drying process.

### 2.7 Structural characterization data of target compounds

Ethyl 2-(2-(benzo[d]thiazol-2-yl)phenoxy)acetate (**A1**) White solid; Yield/%: 78%; Mp/°C: 73.4∼75.4; ESI-MS [M + H]^+^: 314.6; ^1^H NMR (600 MHz, CDCl_3_) δ ppm: 8.556 (d, *J* = 7.8 Hz, 1H, Ph-H), 8.096 (d, *J* = 8.2 Hz, 1H, Ph-H), 7.935 (d, *J* = 8.0 Hz, 1H, Ph-H), 7.491 (t, *J* = 7.68 Hz, 1H, Ph-H), 7.439 (t, *J* = 7.86 Hz, 1H, Ph-H), 7.377 (t, *J* = 7.62 Hz, 1H, Ph-H), 7.199 (t, *J* = 7.56 Hz, 1H, Ph-H), 6.969 (d, *J* = 8.28 Hz, 1H, Ph-H), 4.866 (s, 2H, -OCH_2_), 4.299–4.335 (m, 2H, CH_2_), 1.316 (t, *J* = 7.14 Hz, 3H, CH_3_). ^13^C NMR (150 MHz, CDCl_3_) δ ppm: 168.2 (-C=O), 163.1 (thiazole-C), 155.6 (benzothiazole-C), 136.3 (benzothiazole-C), 131.9 (Ph-C), 130.2 (Ph-C), 126.1 (Ph-C), 124.9 (Ph-C), 122.9 (Ph-C), 122.4 (Ph-C), 121.4 (Ph-C), 112.6 (Ph-C), 66.1 (-OCH_2_COOCH_2_CH_3_), 61.7(-OCH_2_COOCH_2_CH_3_), 14.3(-OCH_2_COOCH_2_CH_3_). Anal. Calcd for C_17_H_15_NO_3_S: C, 65.16%; H, 4.82%; N, 4.47%; Found: C, 65.18%; H, 4.83%; N, 4.46%.

2-(2-((2-nitrobenzyl)oxy)phenyl)benzo[d]thiazole (**A2**) White solid; Yield/%: 81%; Mp/°C: 115.6∼117.6; ESI-MS [M + H]^+^: 363.2; ^1^H NMR (600 MHz, CDCl_3_) δ ppm: 8.502 (d, *J* = 7.8 Hz, 1H, Ph-H), 8.231 (d, *J* = 8.22 Hz, 1H, Ph-H), 8.115 (d, *J* = 8.16 Hz, 1H, Ph-H), 7.971 (d, *J* = 7.86 Hz, 1H, Ph-H), 7.905 (d, *J* = 7.92 Hz, 1H, Ph-H), 7.687 (t, *J* = 7.5 Hz, 1H, Ph-H), 7.496–7.547 (m, 2H, Ph-H), 7.373–7.440 (m, 2H, Ph-H), 7.182 (t, *J* = 7.5 Hz, 1H, Ph-H), 7.036 (d, *J* = 8.34 Hz, 1H, Ph-H), 5.792 (s, 2H, -OCH_2_). ^13^C NMR (150 MHz, CDCl_3_) δ ppm: 163.1 (thiazole-C), 155.8 (benzothiazole-C), 152.3 (Ph-C), 147.1 (Ph-C), 136.0 (Ph-C), 134.3 (Ph-C), 133.3 (benzothiazole-C), 132.0 (Ph-C), 130.4 (Ph-C), 129.1 (Ph-C), 128.8 (Ph-C), 126.2 (Ph-C), 125.3 (Ph-C), 125.0 (Ph-C), 123.0 (Ph-C), 122.2 (Ph-C), 121.4 (Ph-C), 113.4 (Ph-C), 68.2(-OCH_2_-). Anal. Calcd for C_20_H_14_N_2_O_3_S: C, 66.29%; H, 3.89%; N, 7.73%; Found: C, 66.31%; H, 3.88%; N, 7.74%.

2-(3-(2-(benzo[d]thiazol-2-yl)phenoxy)propyl)isoindoline-1,3-dione (**A3**) Pale yellow solid; Yield/%: 67%; Mp/°C: 141.6∼143.6; ESI-MS[M + H]^+^: 415.1; ^1^H NMR (600 MHz, CDCl_3_) δ ppm: 8.476 (dd, *J* = 7.8, 1.8 Hz, 1H, Ph-H), 8.033 (d, *J* = 8.4 Hz, 1H, Ph-H), 7.978 (d, *J* = 8.4 Hz, 1H, Ph-H), 7.721–7.735 (m, 2H, Ph-H), 7.585–7.599 (m, 2H Ph-H), 7.421–7.486 (m, 2H, Ph-H), 7.374 (t, *J* = 7.8 Hz, 1H, Ph-H), 7.115 (t, *J* = 7.68 Hz, 1H, Ph-H), 7.033 (d, *J* = 8.4 Hz, 1H, Ph-H), 4.319 (t, *J* = 5.88 Hz, 2H, -OCH_2_), 4.080 (t, *J* = 6.84 Hz, 2H, -CH_2_), 2.418–2.460 (m, 2H, -CH_2_). ^13^C NMR (150 MHz, CDCl_3_) δ ppm: 168.5 (-C=O), 163.3 (thiazole-C), 156.5 (benzothiazole-C), 135.9 (benzothiazole-C), 133.9 (Ph-C), 132.1 (Ph-C), 129.8 (Ph-C), 126.1 (Ph-C), 124.8 (Ph-C), 123.1 (Ph-C), 122.6 (Ph-C), 121.6 (Ph-C), 112.2 (Ph-C), 67.3 (-OCH_2_CH_2_CH_2_-), 36.1 (-OCH_2_CH_2_CH_2_-), 28.8 (-OCH_2_CH_2_CH_2_-). Anal. Calcd for C_24_H_18_N_2_O_3_S: C, 69.55%; H, 4.38%; N, 6.76%; Found: C,69.51%; H, 4.37%; N, 6.75%.

2-(2-((4-nitrobenzyl)oxy)phenyl)benzo[d]thiazole (**A4**) White solid; Yield/%: 70%; Mp/°C: 135.5∼137.5; ESI-MS [M + H]^+^: 363.2; ^1^H NMR (600 MHz, CDCl_3_) δ ppm: 8.535 (dd, *J* = 8.16, 2.4 Hz, 1H, Ph-H), 8.287 (d, *J* = 8.58 Hz, 2H, Ph-H), 8.101 (d, *J* = 8.16 Hz, 1H, Ph-H), 7.897 (d, *J* = 7.98 Hz, 1H, Ph-H), 7.733 (d, *J* = 8.58 Hz, 2H, Ph-H), 7.503 (t, *J* = 7.86 Hz, 1H, Ph-H), 7.370–7.454 (m, 2H, Ph-H), 7.184 (t, *J* = 7.62 Hz, 1H, Ph-H), 7.057 (d, *J* = 8.28 Hz, 1H, Ph-H), 5.426 (s, 2H,-OCH_2_). ^13^C NMR (150 MHz, CDCl_3_) δ ppm: 162.9 (thiazole-C), 155.8 (benzothiazole-C), 148.0 (Ph-C), 143.4 (Ph-C), 135.8 (Ph-C), 132.1 (benzothiazole-C), 130.4 (Ph-C), 128.3 (Ph-C), 126.3 (Ph-C), 125.1 (Ph-C), 124.1 (Ph-C), 122.9 (Ph-C), 122.2 (Ph-C), 121.4 (Ph-C), 113.0 (Ph-C), 69.9 (-OCH_2_-). Anal. Calcd for C_20_H_14_N_2_O_3_S: C, 66.29%; H, 3.89%; N, 7.73%; Found: C, 66.30%; H, 3.90%; N, 7.75%.

2-(2-((3-chlorobenzyl)oxy)phenyl)benzo[d]thiazol (**A5**) White solid; Yield/%: 65%; Mp/°C: 53.4∼55.4; ESI-MS [M + H]^+^: 352.1; ^1^H NMR (600 MHz, CDCl_3_) δ ppm: 8.546 (dd, *J* = 7.68, 1.38 Hz, 1H, Ph-H), 8.098 (d, *J* = 8.10 Hz, 1H, Ph-H), 7.899 (d, *J* = 7.92 Hz, 1H, Ph-H), 7.567 (s, 1H, Ph-H), 7.491 (t, *J* = 8.04 Hz, 1H, Ph-H), 7.347–7.429 (m, 5H, Ph-H), 7.158 (t, *J* = 7.74 Hz, 1H, Ph-H), 7.069 (d, *J* = 8.34 Hz, 1H, Ph-H), 5.311 (s, 2H, -OCH_2_). ^13^C NMR (150 MHz, CDCl_3_) δ ppm: 156.1 (thiazole-C), 138.3 (Ph-C) (benzothiazole-C), 136.1 (Ph-C), 134.7 (benzothiazole-C), 131.9 (Ph-C), 130.1 (Ph-C), 128.5 (Ph-C), 127.9 (Ph-C), 126.1 (Ph-C), 125.9 (Ph-C), 124.8 (Ph-C), 122.9 (Ph-C), 121.8 (Ph-C), 121.4 (Ph-C), 113.0 (Ph-C), 70.4 (-OCH_2_-). Anal. Calcd for C_20_H_14_ClNOS: C, 68.27%; H, 4.01%; N, 3.98%; Found: C, 68.28%; H, 4.02%; N, 3.99%.

2-(2-((4-methylbenzyl)oxy)phenyl)benzo[d]thiazole (**A6**) White solid; Yield/% 77%; Mp/°C: 57.3∼59.3; ESI-MS [M + H]^+^: 332.0; ^1^H NMR (600 MHz, CDCl_3_) δ ppm: 8.555 (dd, *J* = 7.98, 1.32 Hz, 1H, Ph-H), 8.082 (d, *J* = 8.16Hz, 1H, Ph-H), 7.883 (d, *J* = 7.92Hz, 1H, Ph-H), 7.402–7.489 (m, 4H, Ph-H), 7.351 (t, *J* = 7.38 Hz, 1H, Ph-H), 7.225 (d, *J* = 7.8 Hz, 2H, Ph-H), 7.124 (dd, *J* = 7.56, 5.58 Hz, 2H, Ph-H), 5.309 (s, 2H, -OCH_2_), 2.390 (s, 3H, -CH_3_). ^13^C NMR (150 MHz, CDCl_3_) δ ppm: 163.2 (thiazole-C), 156.3 (Ph-C), (benzothiazole-C), 138.0 (Ph-C), 136.1 (Ph-C), 133.0 (benzothiazole-C), 131.7 (Ph-C), 129.3 (Ph-C), 127.9 (Ph-C), 125.9 (Ph-C), 124.5 (Ph-C), 122.7 (Ph-C), 121.3 (Ph-C), 121.3 (Ph-C), 113.0 (Ph-C), 70.9 (-OCH_2_-), 21.2 (-CH_3_). Anal. Calcd for C_21_H_17_NOS: C, 76.10%; H, 5.17%; N, 4.23%; Found: C, 76.08%; H, 5.16%; N, 4.22%.

2-(2-(benzyloxy)phenyl)benzo[d]thiazole (**A7**) Gray solid; Yield/%: 70%; Mp/°C: 89.7∼91.7; ESI-MS [M + H]^+^: 318.4; ^1^H NMR (600 MHz, CDCl_3_) δ ppm: 8.557 (dd, *J* = 7.98, 1.50 Hz, 1H, Ph-H), 8.089 (d, *J* = 8.22 Hz, 1H, Ph-H), 7.879 (d, *J* = 7.98 Hz, 1H, Ph-H), 7.546 (d, *J* = 7.32 Hz, 2H, Ph-H), 7.481 (t, *J* = 8.04 Hz, 1H, Ph-H), 7.342–7.435 (m, 5H, Ph-H), 7.102–7.151 (m, 2H, Ph-H), 5.352(s, 2H, -OCH_2_). ^13^C NMR (150 MHz, CDCl_3_) δ ppm: 163.3 (thiazole-C), 156.3 (benzothiazole-C), 136.0 (Ph-C), 131.9 (Ph-C), (benzothiazole-C), 129.9 (Ph-C), 128.6 (Ph-C), 128.3 (Ph-C), 127.8 (Ph-C), 126.0 (Ph-C), 124.7 (Ph-C), 122.6 (Ph-C), 121.5 (Ph-C), 121.3 (Ph-C), 113.0 (Ph-C), 71.0 (-OCH_2_-). Anal. Calcd for C_20_H_15_NOS: C, 75.68%; H, 4.76%; N, 4.41%; Found: C, 75.70%; H, 4.77%; N, 4.42%.

4-((2-(benzo[d]thiazol-2-yl)phenoxy)methyl)benzonitrile (**A8**) Gray solid; Yield/%: 66%; Mp/°C: 131.8∼133.8; ESI-MS [M + H]^+^: 343.1; ^1^H NMR (600 MHz, CDCl_3_) δ ppm: 8.546 (d, *J* = 7.8 Hz, 1H, Ph-H), 8.098 (d, *J* = 8.16 Hz, 1H, Ph-H), 7.894 (d, *J* = 7.92 Hz, 1H, Ph-H), 7.717 (d, *J* = 8.1Hz, 2H, Ph-H), 7.660 (d, *J* = 8.04 Hz, 2H, Ph-H), 7.501 (t, *J* = 7.74 Hz, 1H, Ph-H), 7.431 (t, *J* = 8.1 Hz,1H, Ph-H), 7.381 (t, *J* = 7.68Hz, 1H, Ph-H), 7.175 (t, *J* = 7.56 Hz, 1H, Ph-H), 7.041 (d, *J* = 8.34 Hz, 1H, Ph-H), 5.380 (s, 2H, -OCH_2_). ^13^C NMR (150 MHz, CDCl_3_) δ ppm: 155.7 (thiazole-C), 141.4 (benzothiazole-C), 132.5 (benzothiazole-C), 131.9 (Ph-C), 130.2 (Ph-C), 128.1 (Ph-C), 126.2 (Ph-C), 124.9 (Ph-C), 122.8 (Ph-C), 122.0 (Ph-C), 121.3 (Ph-C), 118.5 (-CN), 112.8 (Ph-C), 112.2 (Ph-C), 70.1 (-OCH_2_-). Anal. Calcd for C_21_H_14_N_2_OS: C, 73.66%; H, 4.12%; N, 8.18%; Found: C, 73.64%; H, 4.11%; N, 8.17%.

6-fluoro-*N*-phenethylbenzo[d]thiazol-2-amine (**B1**) White solid; Yield/%: 86%; Mp/°C: 132.4∼134.6; ESI-MS [M + H]^+^: 273.1; ^1^H NMR (400 MHz, DMSO-*d*_*6*_) δ ppm: 12.85 (s, 1H, -NH), 8.07–8.04 (m, 1H, Ph-H), 7.85–7.79 (m, 3H, Ph-H), 7.60 (dd, *J* = 9.9, 2.5 Hz, 1H, Ph-H), 7.25–7.13 (m, 3H, Ph-H), 3.89 (s, 2H, -CH_2_), 3.87 (s, 2H, -CH_2_). ^13^C NMR (101 MHz, DMSO-*d*_*6*_) δ ppm: 165.6 (thiazole-C), 163.0 (Ph-C), 161.9 (Ph-C), 160.6 (Ph-C), 153.2 (Ph-C), 148.9 (benzothiazole-C), 123.4 (C, d, *J*_C−C−C−F_ = 10.10 Hz), 122.9 (Ph-C), 112.2 (Ph-C), 111.9 (Ph-C), 111.6 (C, d, *J*_C−C−C−C−F_ = 4.04 Hz), 106.8 (C, d, *J*_C−C−F_ = 24.24 Hz), 56.2 (-CH_2_CH_2_-), 56.1 (-CH_2_CH_2_-). Anal. Calcd for C_15_H_13_FN_2_S: C, 66.15%; H, 4.81%; N, 10.29%; Found: C, 66.16%; H, 4.82%; N, 10.30%.

*N*-(2,6-dichlorobenzyl)-6-fluorobenzo[d]thiazol-2-amine (**B2**) Gray solid; Yield/%: 77%; Mp/°C: 156.8∼158.2; ESI-MS [M + H]^+^: 327.0; ^1^H NMR (400 MHz, DMSO-*d*_*6*_) δ ppm: 8.57 (d, *J* = 4.5 Hz, 1H, Ph-H), 7.70 (dd, *J* = 8.6, 5.6 Hz, 1H, Ph-H), 7.55 (d, *J* = 8.1 Hz, 2H, Ph-H), 7.43 (dd, *J* = 8.7, 7.5 Hz, 1H, Ph-H), 7.28 (dd, *J* = 10.4, 2.5 Hz, 1H, Ph-H), 6.99–6.85 (m, 1H, Ph-H), 4.83 (s, 2H, -CH_2_). ^13^C NMR (101 MHz, DMSO-*d*_*6*_) δ ppm: 168.1 (thiazole-C), 163.0 (Ph-C), 160.6 (benzothiazole-C), 136.2 (Ph-C), 132.9 (Ph-C), 131.2 (Ph-C), 129.1 (Ph-C), 126.0 (benzothiazole-C), 122.5 (C, d, *J*_C−C−C−F_ = 10.1 Hz), 109.1(C, d, *J*_C−C−F_ = 24.24 Hz), 105.1(C, d, *J*_C−C−F_ = 24.25 Hz), 44.1 (-CH_2_-). Anal. Calcd for C_14_H_9_Cl_2_FN_2_S: C, 51.39%; H, 2.77%; N, 8.56%; Found: C, 51.37%; H, 2.76%; N, 8.55%.

*N*-(3,4-dimethoxybenzyl)-6-fluorobenzo[d]thiazol-2-amine (**B3**) Gray solid; Yield/%: 79%; Mp/°C: 135.5∼137.4; ESI-MS [M + H]^+^: 319.1; ^1^H NMR (400 MHz, DMSO-*d*_*6*_) δ(ppm): 8.56 (s, 1H, -NH), 7.43 (dd, *J* = 8.3, 5.5 Hz, 1H, Ph-H), 7.11 (s, 1H, Ph-H), 6.99–6.75 (m, 4H, Ph-H), 5.08 (s, 2H, -CH_2_). 3.73 (d, *J* = 5.1 Hz, 6H, -OCH_3_). ^13^C NMR (101 MHz, DMSO-*d*_*6*_) δ ppm: 163.0 (thiazole-C), 160.6 (Ph-C), 160.0 (Ph-C), 149.2 (benzothiazole-C), 148.5 (Ph-C), 141.9 (Ph-C), 141.8 (Ph-C), 129.2 (benzothiazole-C), 123.1 (C, d, *J*_C−C−C−F_ = 9.09 Hz), 119.8 (Ph-C), 117.9 (Ph-C), 112.1 (C, d, *J*_C−C−F_ = 18.18 Hz), 108.1 (C, d, *J*_C−C−F_ = 23.23 Hz), 98.6 (Ph-C), 98.3 (Ph-C), 55.9 (-OCH_3_), 45.1 (-CH_2_-). Anal. Calcd for C_16_H_15_FN_2_O_2_S: C, 60.36%; H, 4.75%; N, 8.80%; Found: C, 60.38%; H, 4.76%; N, 8.81%.

6-fluoro-*N*-(4-nitrobenzyl)benzo[d]thiazol-2-amine (**B4**) White solid; Yield/%: 65%; Mp/°C: 142.5∼144.7; ESI-MS [M + H]^+^: 304.1; ^1^H NMR (400 MHz, DMSO-*d*_*6*_) δ ppm: 8.86 (t, *J* = 5.9 Hz, 1H, -NH), 8.27–8.21 (m, 2H, Ph-H), 7.70 (dd, *J* = 8.6, 5.6 Hz, 1H, Ph-H), 7.66–7.59 (m, 2H, Ph-H), 7.20 (dd, *J* = 10.5, 2.5 Hz, 1H, Ph-H), 6.91 (td, *J* = 9.3, 2.6 Hz, 1H, Ph-H), 4.76 (d, *J* = 5.9 Hz, 2H, -CH_2_). ^13^C NMR (101 MHz, DMSO-*d*_*6*_) δ ppm: 168.8 (thiazole-C), 153.9 (C, d, *J*_C−C−C−F_ = 12.12 Hz), 147.2 (C, d, *J*_C−C−F_ = 40.40 Hz), 128.6 (Ph-C), 127.5 (Ph-C), 126.4 (benzothiazole-C), 124.1 (Ph-C), 122.4 (C, d, *J*_C−C−F_ = 10.10 Hz), 109.1 (Ph-C), 105.5 (Ph-C), 46.9 (-CH_2_-). Anal. Calcd for C_14_H_10_FN_3_O_2_S: C, 55.44%; H, 3.32%; N, 13.85%; Found: C, 55.47%; H, 3.33%; N, 13.86%.

6-chloro-*N*-(3-fluorobenzyl)benzo[d]thiazol-2-amine (**B5**) White solid; Yield/%: 45%; Mp/°C: 145.6∼147.6; ESI-MS [M + H]^+^: 293.5; ^1^H NMR (600 MHz, CDCl_3_) δ ppm:7.545 (d, *J* = 2.1 Hz, 1H, Ph-H),7.429 (d, *J* = 8.64 Hz, 1H, Ph-H), 7.103–7.246 (m, 5H, Ph-H), 5.149 (s, 1H, -NH), 4.646 (s, 2H, -OCH_2_). ^13^C NMR (150 MHz, CDCl_3_) δ ppm: 130.4 (benzothiazole-C), 130.4 (Ph-C), 127.0 (Ph-C), 126.5 (Ph-C), 123.1 (Ph-C), 123.1 (Ph-C), 120.5 (Ph-C), 119.8 (Ph-C), 114.9 (Ph-C), 114.8 (Ph-C), 114.6 (Ph-C), 114.4 (Ph-C), 48.6 (-CH_2_-). Anal. Calcd for C_14_H_10_ClFN_2_S: C, 57.44%; H, 3.44%; N, 9.57%; Found: C, 57.48%; H, 3.45%; N, 9.58%.

6-chloro-*N*-(3,5-dimethoxybenzyl)benzo[d]thiazol-2-amine (**B6**) Pale yellow solid; Yield/%: 58%; Mp/°C: 101.3∼103.3; ESI-MS [M + H]^+^: 335.1; ^1^H NMR (600 MHz, CDCl_3_) δ ppm: 7.539 (d, *J* = 1.98 Hz, 1H, Ph-H), 7.427 (d, *J* = 8.58 Hz, 1H, Ph-H), 7.236 (s, 1H, Ph-H), 6.342–6.528 (m, 3H, Ph-H), 5.083 (s, 1H, -NH), 4.557(s, 2H, -CH_2_), 3.746 (s, 6H, -OCH_3_). ^13^C NMR (150 MHz, CDCl_3_) δ ppm: 167.5 (thiazole-C), 161.3 (Ph-C),151.0 (benzothiazole-C), 139.6 (Ph-C), 131.8 (benzothiazole-C), 126.5 (Ph-C), 121.5 (Ph-C), 120.6 (Ph-C), 119.7 (Ph-C), 110.7 (Ph-C), 107.0 (Ph-C), 107.0 (Ph-C), 105.7 (Ph-C), 99.8 (Ph-C), 99.1 (Ph-C), 55.5 (-CH_3_), 49.5 (-CH_2_-). Anal. Calcd for C_16_H_15_ClN_2_O_2_S: C, 57.40%; H, 4.52%; N, 8.37%; Found: C, 57.36%; H, 4.51%; N, 8.36%.

6-chloro-*N*-(4-nitrobenzyl)benzo[d]thiazol-2-amine (**B7**) Pale yellow solid; Yield/%: 31%; Mp/°C: 148.4∼150.8; ESI-MS [M + H]^+^: 320.4; ^1^H NMR (600 MHz, CDCl_3_) δ ppm: 8.412 (d, *J* = 8.4 Hz, 1H, Ph-H), 8.216 (d, *J* = 8.4 Hz, 2H, Ph-H), 8.199 (s, 1H, Ph-H), 8.145 (d, *J* = 8.4 Hz, 1H, Ph-H), 7.451 (d, *J* = 8.4 Hz, 2H, Ph-H), 4.850 (s, 2H, -CH_2_), 3.077 (s, 1H, -NH). ^13^C NMR (150 MHz, CDCl_3_) δ ppm: 167.5 (thiazole-C), 158.7 (benzothiazole-C),151.1 (Ph-C), 129.6 (Ph-C), 129.5 (benzothiazole-C), 126.6 (Ph-C), 121.6 (Ph-C), 115.8 (Ph-C), 110.5 (Ph-C), 48.7 (-CH_2_-). Anal. Calcd for C_14_H_10_ClN_3_O_2_S: C, 52.59%; H, 3.15%; N, 13.14%; Found: C, 52.61%; H, 3.14%; N, 13.13%.

*N*-benzyl-6-chlorobenzo[d]thiazol-2-amine (**B8**) Pale yellow solid; Yield/%: 50%; Mp/°C: 123.5∼125.5; ESI-MS [M + H]^+^: 275.1; ^1^H NMR (600 MHz, CDCl_3_) δ ppm: 7.540 (d, *J* = 2.04 Hz, 1H, Ph-H), 7.357–7.394 (m, 3H, Ph-H), 7.305–7.332 (m, 3H, Ph-H), 7.243 (d, *J* = 2.04 Hz, 1H, Ph-H), 5.160 (s, 1H, -NH), 4.631(s, 2H, -CH_2_). ^13^C NMR (125 MHz, DMSO-*d*_*6*_) δ ppm: 166.9 (thiazole-C), 151.2 (benzothiazole-C), 138.5 (Ph-C), 131.9 (benzothiazole-C), 128.6 (Ph-C), 128.3 (Ph-C), 127.3 (Ph-C), 127.1 (Ph-C), 126.8 (Ph-C), 125.7 (Ph-C), 124.7 (Ph-C), 120.5 (Ph-C), 118.9 (Ph-C), 47.2 (-CH_2_-). Anal. Calcd for C_14_H_11_ClN_2_S: C, 61.20%; H, 4.04%; N, 10.20%; Found: C, 61.17%; H, 4.03%; N, 10.19%.

*N*-(6-fluorobenzo[d]thiazol-2-yl)benzamide (**C1**) Gray solid; Yield/%: 45%; Mp/°C: >300; ESI-MS [M + H]^+^: 273.1; ^1^H NMR (400 MHz, DMSO-*d*_*6*_) δ ppm: 12.99 (s, 1H, -CONH), 8.21–8.11 (m, 2H, Ph-H), 8.06 (dd, *J* = 8.7, 5.5 Hz, 1H, Ph-H), 7.68 (t, *J* = 7.4 Hz, 1H, Ph-H), 7.65–7.53 (m, 3H, Ph-H), 7.23 (td, *J* = 9.1, 2.4 Hz, 1H, Ph-H). ^13^C NMR (101 MHz, DMSO-*d*_*6*_) δ 166.4 (thiazole-C), 163.0 (Ph-C), 161.6 (Ph-C), 160.6 (Ph-C), 133.4 (Ph-C), 132.1 (Ph-C), 129.1 (Ph-C), 128.8 (Ph-C), 127.8 (benzothiazole-C), 123.5 (C, d, *J*_C−C−C−F_ = 10.10 Hz), 112.2 (C, d, *J*_C−C−F_ = 24.24 Hz), 107.0 (C, d, *J*_C−C−F_ = 24.24 Hz). Anal. Calcd for C_14_H_9_FN_2_OS: C, 61.75%; H, 3.33%; N, 10.29%; Found: C, 61.73%; H, 3.32%; N, 10.28%.

2,6-dichloro-*N*-(6-fluorobenzo[d]thiazol-2-yl) benzamide (**C2**) White solid; Yield/%: 51%; Mp/°C: >300; ESI-MS [M + H]^+^: 341.0; ^1^H NMR (400 MHz, DMSO-*d*_*6*_) δ ppm: 13.32 (s, 1H, -CONH), 8.09 (dd, *J* = 8.8, 5.5 Hz, 1H, Ph-H), 7.73–7.48 (m, 4H, Ph-H), 7.26 (td, *J* = 9.1, 2.5 Hz, 1H, Ph-H). ^13^C NMR (101 MHz, DMSO-*d*_*6*_) δ ppm: 163.8 (thiazole-C), 163.1 (Ph-C), 160.7 (Ph-C), 160.1 (Ph-C), 150.0 (C, d, *J*_C−C−C−F_ = 12.12 Hz), 134.6 (Ph-C), 132.8 (Ph-C), 131.6 (Ph-C), 128.8 (benzothiazole-C), 127.8 (Ph-C), 123.7 (C, d, *J*_C−C−C−F_ = 10.10 Hz), 112.7 (C, d, *J*_C−C−F_ = 24.24 Hz), 107.6 (C, d, *J*_C−C−F_ = 24.24 Hz). Anal. Calcd for C_14_H_7_Cl_2_FN_2_OS: C, 49.29%; H, 2.07%; N, 8.21%; Found: C, 49.31%; H, 2.08%; N, 8.22%.

*N*-(6-fluorobenzo[d]thiazol-2-yl)-3,4-dimethoxybenzamide (**C3**) White solid; Yield/%: 46%; Mp/°C: >300; ESI-MS [M + H]^+^: 333.1; ^1^H NMR (400 MHz, DMSO-*d*_*6*_) δ ppm: 12.86 (s, 1H, -CONH), 8.05 (dd, *J* = 8.7, 5.5 Hz, 1H, Ph-H), 7.93–7.74 (m, 2H, Ph-H), 7.60 (dd, *J* = 10.0, 2.2 Hz, 1H, Ph-H), 7.22 (td, *J* = 9.1, 2.4 Hz, 1H, Ph-H), 7.14 (d, *J* = 8.5 Hz, 1H, Ph-H), 3.88 (d, *J* = 7.1 Hz, 6H, -OCH_3_). ^13^C NMR (101 MHz, DMSO-*d*_*6*_) δ ppm: 165.6 (thiazole-C), 163.0 (Ph-C), 161.9 (Ph-C), 160.6 (Ph-C), 153.2 (Ph-C), 150.1 (benzothiazole-C), 148.8 (Ph-C), 127.8 (Ph-C), 123.7 (C, d, *J*_C−C−C−F_ = 55.55 Hz), 122.8 (Ph-C), 111.8 (C, d, *J*_C−C−F_ = 32.32 Hz), 111.6, 106.9 (C, d, *J*_C−C−F_ = 25.25 Hz), 56.2 (-OCH_3_). Anal. Calcd for C_16_H_13_FN_2_O_3_S: C, 57.82%; H, 3.94%; N, 8.43%; Found: C, 57.79%; H, 3.93%; N, 8.42%.

*N*-(6-fluorobenzo[d]thiazol-2-yl)-4-nitrobenzamide (**C4**) White solid; Yield/%: 60%; Mp/°C:187.7∼189.4; ESI-MS [M + H]^+^: 319.1; ^1^H NMR (400 MHz, CF_3_COOD) δ ppm: δ 8.61 (dd, *J* = 63.9, 8.5 Hz, 4H, Ph-H), 8.21 (dd, *J* = 9.2, 4.4 Hz, 1H, Ph-H), 7.89 (d, *J* = 7.7 Hz, 1H, Ph-H), 7.64 (t, *J* = 8.9 Hz, 1H, Ph-H). ^13^C NMR (101 MHz, CF_3_COOD) δ ppm: 165.44 (thiazole-C), 151.2 (benzothiazole-C), 136.0 (C, d, *J*_C−C−C−F_ = 13.13 Hz), 134.8 (Ph-C), 129.8 (benzothiazole-C), 124.4 (Ph-C), 121.1 (Ph-C), 118.6 (Ph-C), 116.7 (C, d, *J*_C−C−F_ = 25.25 Hz), 115.8 (Ph-C), 113.0 (Ph-C), 110.18 (Ph-C), 103.3 (C, d, *J*_C−C−F_ = 28.28 Hz). Anal. Calcd for C_14_H_8_FN_3_O_3_S: C, 53.00%; H, 2.54%; N, 13.24%; Found: C, 53.03%; H, 2.55%; N, 13.22%.

*N*-(6-chlorobenzo[d]thiazol-2-yl)-4-methoxybenzamide (**C5**) Gray solid; Yield/%: 70%; Mp/°C: >300, ESI-MS [M + H]^+^: 319.1; ^1^H NMR (600 MHz, CDCl_3_) δ ppm: 10.081 (s, 1H, -NH), 7.947 (d, *J* = 8.3 Hz, 2H, Ph-H), 7.813 (s, 1H, Ph-H), 7.376 (d, *J* = 8.6 Hz, 1H, Ph-H), 7.291 (d, *J* = 8.6 Hz, 1H, Ph-H), 3.862 (s, 3H, -OCH_3_). ^13^C NMR (150 MHz, DMSO-*d*_*6*_) δ ppm: 166.1 (thiazole-C), 163.3 (-C=O), 160.7 (Ph-C), 147.8 (benzothiazole-C), 133.7 (benzothiazole-C), 130.9 (Ph-C), 127.9 (Ph-C), 126.8 (Ph-C), 124.4 (Ph-C), 121.8 (Ph-C), 121.7 (Ph-C), 114.4 (Ph-C), 56.0 (-OCH_3_). Anal. Calcd for C_15_H_11_ClN_2_O_2_S: C, 56.52%; H, 3.48%; N, 8.79%; Found: C, 56.59%; H, 3.47%; N, 8.78%.

*N*-(6-chlorobenzo[d]thiazol-2-yl)benzamide (**C6**) Gray solid; Yield/%: 82%; Mp/°C: >300; ESI-MS [M + H]^+^: 289.1; ^1^H NMR (600 MHz, CDCl_3_) δ ppm: 11.011 (s, 1H, -NH), 7.985 (d, *J* = 7.86 Hz, 2H, Ph-H), 7.819 (s, 1H, Ph-H), 7.610 (t, *J* = 7.1 Hz, 2H, Ph-H), 7.481 (d, *J* = 7.5 Hz, 2H, Ph-H), 7.273 (s, 1H, Ph-H). ^13^C NMR (150 MHz, DMSO-*d*_*6*_) δ ppm: 166.9 (thiazole-C), 160.6 (-C=O), 147.7 (benzothiazole-C), 133.7 (Ph-C), 133.3 (benzothiazole-C), 132.5 (Ph-C), 129.1 (Ph-C), 128.7 (Ph-C), 128.0 (Ph-C), 126.9 (Ph-C), 121.9 (Ph-C), 121.7 (Ph-C). Anal. Calcd for C_14_H_9_ClN_2_OS: C, 58.24%; H, 3.14%; N, 9.70%; Found: C, 58.20%; H, 3.13%; N, 9.71%.

*N*-(6-chlorobenzo[d]thiazol-2-yl)-2-fluorobenzamide (**C7**) White solid; Yield/%: 77%; Mp/°C: >300; ESI-MS [M + H]^+^: 307.0; ^1^H NMR (600 MHz, CDCl_3_) δ ppm: 11.755 (s, 1H, -NH), 9.562 (s, 1H, Ph-H), 9.444 (d, *J* = 8.58 Hz, 1H, Ph-H), 9.350–9.385 (m, 1H, Ph-H), 9.105–9.155 (m, 2H, Ph-H), 9.006 (s, 1H, Ph-H), 8.971 (s, 1H, Ph-H). ^13^C NMR (1500 MHz, DMSO-*d*_*6*_) δ ppm: 166.0 (thiazole-C), 165.9 (-C=O), 164.2(benzothiazole-C), 133.4(benzothiazole-C), 131.7 (Ph-C), 131.6 (Ph-C), 128.2 (Ph-C), 127.0 (Ph-C), 122.0 (Ph-C), 121.8 (Ph-C), 116.2 (Ph-C), 116.1 (Ph-C). Anal. Calcd for C_14_H_8_ClFN_2_OS: C, 54.82%; H, 2.63%; N, 9.13%; Found: C, 54.79%; H, 2.62%; N, 9.14%.

6-chloro-*N,N*-bis(3,5-dimethoxybenzyl)benzo[d]thiazol-2-amine (**D1**) White solid; Yield/%: 20%; Mp/°C: 83.4∼85.3; ESI-MS [M + H]^+^: 485.1; ^1^H NMR (600 MHz, CDCl_3_) δ ppm: 7.533 (d, *J* = 2.04 Hz, 1H, Ph-H), 7.311 (d, *J* = 2.04 Hz, 1H, Ph-H), 6.331–6.549 (m, 7H, Ph-H), 4.663 (s, 4H, -CH_2_), 3.743 (s, 12H, -OCH_3_). ^13^C NMR (150 MHz, CDCl_3_) δ ppm: 169.2 (thiazole-C), 161.2 (Ph-C), 151.6 (benzothiazole-C), 138.6 (Ph-C), 132.3 (benzothiazole-C), 126.5 (Ph-C), 123.9 (Ph-C), 122.2 (Ph-C), 120.4 (Ph-C), 119.7 (Ph-C), 100.0 (Ph-C), 99.6 (Ph-C), 55.4 (-CH_2_-, -OCH_3_). Anal. Calcd for C_25_H_25_ClN_2_O_4_S: C, 61.91%; H, 5.20%; N, 5.78%; Found: C, 61.93%; H, 5.21%; N, 5.79%.

*N,N*-dibenzyl-6-chlorobenzo[d]thiazol-2-amine (**D2**) Gray solid; Yield/%: 21%; Mp/°C: 134.4∼136.4; ESI-MS [M + H]^+^: 365.1; ^1^H NMR (600 MHz, CDCl_3_) δ ppm: 7.321–7.360 (m, 10H, Ph-H), 7.282 (d, *J* = 2.34 Hz, 1H, Ph-H), 7.059 (dd, J = 8.7, 2.34 Hz, 1H, Ph-H), 6.962 (d, *J* = 8.52 Hz, 1H, Ph-H), 4.484 (s, 2H, -CH_2_), 4.178 (s, 2H, -CH_2_). ^13^C NMR (150 MHz, DMSO-*d*_*6*_) δ ppm: 136.1 (benzothiazole-C), 134.1 (Ph-C), 133.0 (Ph-C), 129.0 (Ph-C), 128.9 (Ph-C), 128.6 (Ph-C), 128.5 (Ph-C), 127.6 (Ph-C), 127.4 (Ph-C), 126.4 (Ph-C), 56.6 (-CH_2_-). Anal. Calcd for C_21_H_17_ClN_2_S: C, 69.13%; H, 4.70%; N, 7.68%; Found: C, 69.16%; H, 4.71%; N, 7.69%.

### 2.8 Oil-water partition coefficient (log P) measurement experiment

The partition coefficient between n-octanol and water (log Po/w) is the classical descriptor for Lipophilicity (Daina et al., 2017). The test methodology followed previously published protocols (Wang et al., 2021). At room temperature, two large Erlenmeyer flasks were taken and filled with n-octanol and water, respectively. The flasks were placed in a constant temperature shaker at 150 rpm for 24 h to saturate the solvents. The mixtures were then transferred to separating funnels under normal pressure to obtain water-saturated n-octanol and n-octanol-saturated water for subsequent use. The target compound (2 mg) was accurately weighed and placed in a 2 mL brown volumetric flask. Anhydrous methanol was added to ultrasonically dissolve the compound, and the mixture was diluted to volume and thoroughly shaken to prepare a 1 mg/mL stock solution. The stock solution was further diluted to obtain 1, 1.5, 2, 2.5, 5 and 10 μg/mL reference solutions for construction of a standard curve using a UV-visible spectrophotometer. An excess amount of the analyte was dissolved in water-saturated n-octanol, and the mixture was shaken at 150 rpm for 24 h at constant temperature to obtain a saturated solution. The saturated solution was allowed to stand, centrifuged, and 1 mL of the supernatant was transferred to a 4 mL centrifuge tube. N-octanol saturated water (1 mL) was added, and the mixture was shaken at 150 rpm for 24 h at constant temperature. After standing for 8 h, the mixture was centrifuged. Appropriate amounts of the n-octanol phase before and after equilibration were diluted with methanol, and concentrations were determined from the standard curve to obtain C_0_ and C_1_. The concentration in the n-octanol saturated aqueous phase, C_w_, was calculated as C_w_ = C_0_ - C_1_. Thus, log P_o/w_ = log_10_C_0_/C_w_.

### 2.9 Cell lines and cell culture

Mouse monocyte macrophage leukemia cells RAW 264.7, human lung epithelial cells Beas-2b, human epidermoid carcinoma cells A431, non-small cell lung cancer cells A549 and H1299 were purchased from the Shanghai Cell Bank of the Chinese Academy of Sciences Committee. Beas-2b, A549 cells were cultured in DMEM/F12 (11330032, Gibco), and RAW264.7, A431, H1299 cells were cultured in high sugar DMEM (11965092, Gibco). Both media were supplemented with 10% FBS (12484028, Gibco) and 1% penicillin-streptomycin mixture (100×) (10378016, Gibco). Cells were grown in 37°C thermostat incubator (Thermo Fisher, United States) containing 5% CO_2_ and stored in −80°C for short-term storage, and in liquid nitrogen for long-term storage. None of the cell resuscitation passages used in the experiments herein exceeded 20 generations.

### 2.10 Cell proliferation and toxicity assay

Beas-2b, A431, A549 and H1299 cells were cultured in 96-well plates (5 × 10^3^ cells/well) for 16 h, respectively. Subsequently, positive compound **4i** and 25 newly synthesized compounds were added at a final concentration of 10 μM or gradient concentrations (0.01, 0.1, 0.5, 1, 5 and 10 μM), co-incubated with the cells for 48 h. MTT (M8180, Solarbio) solutions (5 mg/mL) were then added and incubated for 4 h in the dark. Then, formazan crystals were dissolved with DMSO (D8371, Solarbio), followed by shaking on a shaker (DRAGONLAB, China) for 10 min. Finally, absorbance at 490 nm wavelength for each well was measured with Microplate reader (Molecular Devices, United States) (Tang et al., 2020; Lee et al., 2021). The results are expressed as mean ± SD from three independent experiments. Cells inhibition rates or IC_50_ values were calculated using GraphPad Prism 9.5.0.

### 2.11 Anti-inflammation activity assay

RAW264.7 cells were cultured in 6-well plates (2×10^5^ cells/well) for 24 h and treated with 25 newly synthesized compounds (final concentration at 10 μM) for 30 min, respectively. Subsequently, the cells were stimulated with 1 μL of LPS (500 ng/mL) (L8880, Solarbio). After 24 h, the supernatant was collected and analyzed using an ELISA kit (EK206 and EK182, MULTISCIENCES) to quantify the levels of inflammatory cytokines IL-6 and TNF-α (Li et al., 2021).

### 2.12 Flow cytometry analysis of cell apoptosis

The A431 and A549 cells were cultured in 6-well plates (1×10^5^ cells/mL) for 16 h. Subsequently, cells were treated with different concentrations (1, 2 and 4 μM) of either **B7** or **4i** for 24 h. Then, the cells were collected, washed, and stained with the FITC Annexin V Apoptosis Detection Kit I (556547, BD) (Jiang et al., 2017). Sample testing was performed using a FACS Calibur Flow Cytometer (BD, United States), and subsequent data analysis was performed using FlowJo 10.6.2.

### 2.13 Flow cytometry analysis of cell cycle

The A431 and A549 cells were cultured in 6-well plates (1×10^5^ cells/mL) for 16 h. Subsequently, they were treated with different concentrations (1, 2 and 4 μM) of either **B7** or **4i** (4 μM) for 24 h. Cells were collected, mixed by adding 75% ethanol with shaking on a vortex shaker, and placed in the refrigerator at 4°C for overnight fixation. The cells were incubated with PI-Raze solution for 15 min at room temperature, protected from light, according to the instructions of BD Cycletest Plus DNA Reagent Kit (340242, BD), and detected by FACS Calibur flow cytometry (Oh et al., 2023), and subsequent data analysis was performed using FlowJo 10.6.2.

### 2.14 Wound healing analysis of cell migration

The A431 and A549 cells were cultured in 6-well plates (5×10^5^ cells/well). When the cell confluency reached approximately 90%, used a sterile 10 μL pipette tip to create three parallel scratches evenly. Subsequently, rinsed with PBS to remove floating cells, and then treated with **B7** and **4i** at final concentrations of 4 μM. Captured images using a microscope camera system (Nikon, JPN) at 0 and 48 h post-treatment (Xu et al., 2023).

### 2.15 Western blot analysis

The A431 and A549 cells were cultured in 6-well plates (2×10^5^ cells/well) for 2 h and treated with different concentrations of **B7**(1, 5, and 10 μM) or **4i** (10 μM). The corresponding cells were collected, washed with PBS (P1020, Solarbio), and lysed with RIPA buffer (R0010, Solarbio) to extract the total proteins. The extracted protein was loaded, subjected to SDS-PAGE electrophoresis (Bio-Rad) (Xu et al., 2023), and then the protein was transferred to a PDVF membrane (IPVH00010, Millipore) and incubated in the corresponding Primary Antibody AKT (9272, Cell Signaling Technology), phospho-AKT (4058, Cell Signaling Technology), ERK (A4782, ABclonal Technology), phospho-ERK (AP0974, ABclonal Technology) and GAPDH (AB0037, Abways Technology) overnight. Then, the Primary Antibody was recovered and enzyme-labeled secondary antibodies Goat Anti-Rabbit IgG HRP (H + L) (A0208, Beyotime Technology) were used. Finally, Imaging was performed on a gel Imaging System (Bio-Rad, United States) using the Ultra-sensitive ECL Chemiluminescence Assay Kit (P0018AS, Beyotime Technology).

### 2.16 ADMET analysis

The physicochemical parameters, pharmacokinetic properties and drug similarity of the active compound B7 were predicted using Swiss ADME web server (http://www.swissadme.ch/) (Daina et al., 2017). The toxicity associated with compound B7 was predicted by admetSAR web server (http://lmmd.ecust.edu.cn/admetsar2) (Cheng et al., 2012).

### 2.17 Statistical analysis

For the statistical analysis, Microsoft Excel 2016 and GraphPad Prism 9.5.0 software were used. The results were presented as the mean ± standard error of the mean. Statistical analyses were conducted via Student’s t-test. A value of *p* < 0.05 is defined as statistically significant. Statistical significance differences (compared to control group) are defined as follows: *p* > 0.05 (not significant, ns), *p* ≤ 0.05 (*), *p* ≤ 0.01 (**) and *p* ≤ 0.001 (***).

## 3 Results

### 3.1 Chemistry

**SCHEME 2:**
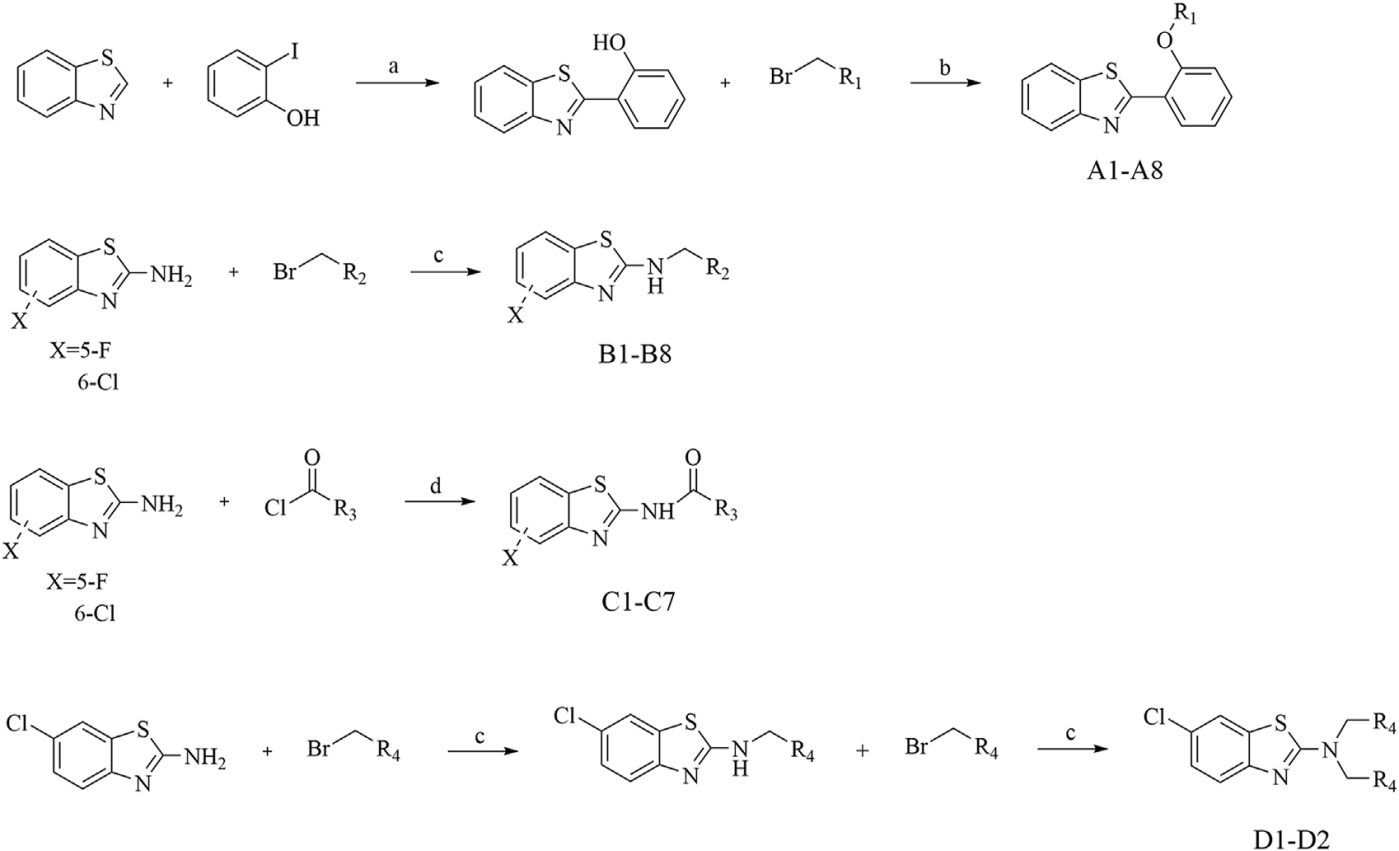
Synthesis of A Series, B Series, C Series, and D Series. Reagents and conditions: **(A)** K_2_CO_3_, DMSO, 120°C, 6 h; **(B)** K_2_CO_3_, acetonitrile, rt, 3 h; **(C)** K_2_CO_3_, acetonitrile, 65°C, 6 h; **(D)** Et_3_N, dioxane, 100°C, 3 h.

The synthetic pathways for the compounds in series A, B, C, and D were elucidated in Scheme 2. Commencing with benzothiazole and 2-hydroxyiodobenzene, and utilizing DMSO as the solvent, 2-phenol-benzothiazole was synthesized through nucleophilic substitution reactions. Subsequent steps involved the use of 2-phenol-benzothiazole and various substituted benzyl bromides as initial reactants, leading to the formation of eight compounds in the A series through the Williamson synthesis method. The B series is created by employing 2-amino-halogenated benzothiazole and diverse substituted benzyl bromides, resulting in the synthesis of eight compounds via amine halogenation reactions. The synthesis of the C series involved the use of 2-amino-halogenated benzothiazole and various substituted acyl chlorides, resulting in the production of seven compounds via amide formation reactions. Similarly, the synthetic procedure for the D series compounds closely resembled that of the B series. To validate the synthesized compounds in this study, thorough analyses utilizing ^1^H NMR, ^13^C NMR, ESI-MS, HPLC and elemental analysis were conducted, confirming the accuracy of their structures (Supplementary Material).

### 3.2 Biological evaluation

#### 3.2.1 Anti-proliferation assay *in vitro*

To assess the anti-proliferative effects of the compounds on cells, we employed the MTT method to analyze the impact of all 2-amino-benzo[d] thiazole derivatives, along with the lead compound **4i**, at a concentration of 10 μM across Beas-2b, A431, A549, and H1299 cells (Tables 1–3). At the same time, we tested the log *p* values of 25 new compounds. According to Lipinski’s rule of five (Ro5), the calculated log *p*-value should be < 5 for compounds intended for oral administration (Lipinski et al., 2001; Danalev et al., 2023). As shown in Tables 1–3, almost all of the compounds had log *p* < 5, which indicates that our newly synthesized compounds have good lipid solubility and have the conditions for drug formation.

**TABLE 1:** Structure-activity relationship of A series and cells inhibition rate (%).

Comp.	R_1_	log P	A431 (%)	A549 (%)	H1299 (%)	Beas-2b (%)
A1	ethyl acetate	2.35 ± 0.21	45.64 ± 1.21	42.34 ± 1.15	49.53 ± 0.84	10.22 ± 0.92
A2	2-nitrobenzyl	3.51 ± 0.19	35.84 ± 2.03	33.15 ± 2.34	42.37 ± 1.26	17.33 ± 3.26
A3	2-butylisoindoline-1,3-dione	3.63 ± 0.16	34.23 ± 1.15	28.54 ± 2.21	30.92 ± 0.77	5.25 ± 0.39
A4	4-nitrobenzyl	3.74 ± 0.35	35.81 ± 1.02	33.32 ± 1.36	39.43 ± 1.20	10.34 ± 0.64
A5	3-chlorobenzyl	4.51 ± 0.24	45.67 ± 0.87	47.62 ± 1.24	35.26 ± 2.24	18.25 ± 2.23
A6	4-methylbenzyl	4.72 ± 0.28	27.66 ± 1.62	20.73 ± 2.31	33.91 ± 0.68	15.11 ± 3.28
A7	benzyl	3.95 ± 0.15	15.31 ± 2.21	12.24 ± 3.42	26.53 ± 1.11	11.36 ± 2.65
A8	4-ethylbenzonitrile	3.44 ± 0.22	28.56 ± 1.24	24.35 ± 1.22	37.29 ± 2.30	14.21 ± 3.65

**TABLE 2:** Structure-activity relationship of B series and cells inhibition rate (%).

Comp.	X	R_2_	log P	A431 (%)	A549 (%)	H1299 (%)	Beas-2b (%)
B1	5-F	N-phenethyl	3.47 ± 0.27	21.13 ± 2.03	18.34 ± 0.93	26.31 ± 0.91	23.12 ± 1.52
B2	5-F	N-(2,6-dichlorobenzyl)	4.25 ± 0.16	27.48 ± 2.42	30.25 ± 2.13	31.29 ± 1.14	18.62 ± 1.67
B3	5-F	N-(3,4-dimethoxybenzyl)	2.56 ± 0.12	32.36 ± 2.92	22.35 ± 1.34	44.63 ± 1.75	25.37 ± 2.01
B4	5-F	N-(4-nitrobenzyl)	2.67 ± 0.25	24.33 ± 1.87	19.62 ± 1.32	25.02 ± 1.62	21.35 ± 2.21
B5	6-Cl	N-(3-fluorobenzyl)	3.58 ± 0.11	59.69 ± 3.62	58.64 ± 1.01	51.34 ± 3.03	20.65 ± 1.85
B6	6-Cl	N-(3,5-dimethoxybenzyl)	3.36 ± 0.18	43.42 ± 1.61	35.23 ± 2.86	32.84 ± 0.74	20.58 ± 1.32
B7	6-Cl	N-(4-nitrobenzyl)	3.25 ± 0.24	78.67 ± 1.75	80.88 ± 1.03	75.72 ± 1.37	16.64 ± 1.36
B8	6-Cl	N-benzyl	3.56 ± 0.16	57.43 ± 3.64	65.75 ± 0.96	55.22 ± 2.49	18.12 ± 3.22

**TABLE 3:** Structure-activity relationship of C and D series and cells inhibition rate (%).

Comp.	X	R_3_, R_4_	log P	A431 (%)	A549 (%)	H1299 (%)	Beas-2b (%)
C1	5-F	N-benzamide	2.56 ± 0.14	17.52 ± 1.35	19.31 ± 1.07	28.34 ± 1.25	16.31 ± 1.82
C2	5-F	N-(2,6-dichlorobenzyl)	2.93 ± 0.11	5.61 ± 0.26	0.27 ± 0.12	6.80 ± 0.22	12.82 ± 1.76
C3	5-F	N-(3,4-dimethoxybenzyl)	2.49 ± 0.39	12.35 ± 1.51	4.38 ± 0.35	17.99 ± 2.52	21.29 ± 1.62
C4	5-F	N-(4-nitrobenzyl)	3.06 ± 0.23	43.11 ± 1.45	35.26 ± 1.82	45.22 ± 0.55	13.45 ± 1.65
C5	6-Cl	N-(4-methoxybenzamide)	2.87 ± 0.15	55.67 ± 2.34	56.33 ± 2.96	51.98 ± 0.24	17.21 ± 1.24
C6	6-Cl	N-benzamide	3.05 ± 0.17	57.73 ± 2.12	55.56 ± 2.78	59.72 ± 1.84	20.56 ± 0.52
C7	6-Cl	N-(2-fluorobenzamide)	2.90 ± 0.32	40.36 ± 1.33	44.64 ± 0.94	48.28 ± 0.37	21.36 ± 2.25
D1	Cl	N, N-bis(3,5-dimethoxybenzyl)	5.69 ± 0.25	38.66 ± 1.56	15.45 ± 3.46	32.66 ± 1.25	19.32 ± 1.31
D2	Cl	N, N-dibenzyl	5.82 ± 0.22	43.63 ± 1.39	40.34 ± 1.06	46.89 ± 2.71	24.31 ± 3.10

The results indicated that following treatment with various compounds, the cell viability of Beas-2b cells remained consistently above 70%, underscoring the safety and reliability of the synthesized compounds at this concentration. The inhibitory rates of A series compounds on A431, A549, and H1299 cancer cells were consistently below 50%. Conversely, compounds from the B series (**B5**, **B7**, and **B8**) as well as those from the C and D series (**C5** and **C6**) demonstrated notable inhibitory effects on these cancer cells, surpassing 50% inhibition rates. Notably, compound **B7** exhibited a potent anti-proliferative effect, with inhibition rates exceeding 75% for all three cancer cell lines.

In summary, the data suggested that the recently synthesized compounds (**B5**, **B7**, **B8**, **C5**, and **C6**) exhibit promising anti-cancer properties without causing notable toxicity. Subsequent research and exploration are deemed necessary.

Then compounds **B5**, **B7**, **B8**, **C5**, and **C6** were assessed at gradient concentrations (0.01, 0.1, 0.5, 1, 5 and 10 μM), revealing significant inhibitory effects on A431, A549, and H1299 cell lines. Subsequently, the IC_50_ tests for A431, A549, and H1299 cells were conducted on the six selected compounds (Table 4). Notably, among these compounds, **B7** exhibited superior inhibitory effects across all three cancer cell lines, with IC_50_ values of 1.51 ± 0.20 μM for A431, 0.96 ± 0.24 μM for A549, and 1.68 ± 1.32 μM for H1299 cells. Remarkably, **B7** surpassed the efficacy of the other five compounds and demonstrated marginally superior inhibitory activity compared to the reference compound **4i**. Determining the IC_50_ values for **B7** lays the groundwork for further biological experiments, offering crucial insights for conducting experiments at specific dosage concentrations.

**TABLE 4:** IC_50_ (mean ± SD) (μM) values of some designed compounds and 4i.

Cell lines	B5 (μM)	B7 (μM)	B8 (μM)	C5 (μM)	C6 (μM)	4i (μM)
A431	10.79 ± 0.56	1.51 ± 0.20	5.36 ± 1.00	4.35 ± 0.90	8.40 ± 1.06	2.00 ± 0.41
A549	5.44 ± 1.00	0.96 ± 0.24	6.25 ± 0.99	9.82 ± 0.98	8.17 ± 0.40	1.09 ± 0.32
H1299	9.26 ± 1.34	1.68 ± 0.12	6.69 ± 0.54	8.94 ± 1.21	9.02 ± 0.37	2.48 ± 0.53

#### 3.2.2 Anti-inflammatory activity assay *in vitro*

**FIGURE 2:**
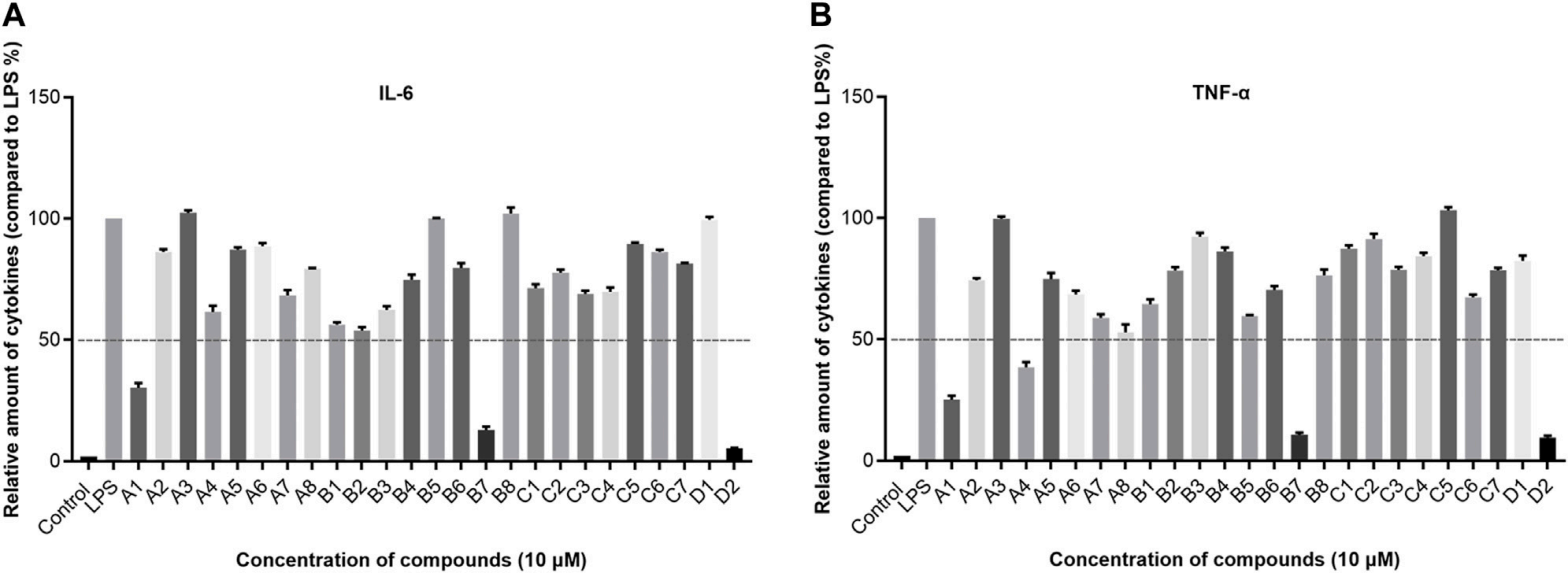
The expression levels of IL-6 and TNF-α *in vitro*. **(A)** The expression of IL-6 after compounds treatment; **(B)** The expression of TNF-α after compounds treatment.

In consideration of the interconnected development of inflammation and tumors, we undertook an initial screening of recently synthesized compounds to assess their anti-inflammatory activity. The parameters under scrutiny encompassed IL-6 and TNF-α, both serving as representative cytokines expressed significantly post-stimulation (Lee et al., 2011; Kumar and Singh, 2021). The down-regulatory effects of each compound on IL-6 and TNF-α were evaluated using ELISA Kit. Specifically, the ELISA method was employed to examine the impact of all recently synthesized compounds on the expression of IL-6 and TNF-α inflammatory factors at a concentration of 10 µM (Figure 2). Combining the expression levels of both inflammatory factors, in comparison to the blank control, compounds **A1**, **B7**, and **D2** demonstrated significant inhibitory effects on IL-6 and TNF-α expression at 10 μM, suggesting potent anti-inflammatory activity. Particularly, **D2** exhibited the most favorable effects, followed by **B7**, which showed efficacy comparable to that of **D2**.

#### 3.2.3 B7 promoted apoptosis of A431 and A549 cells

**FIGURE 3:**
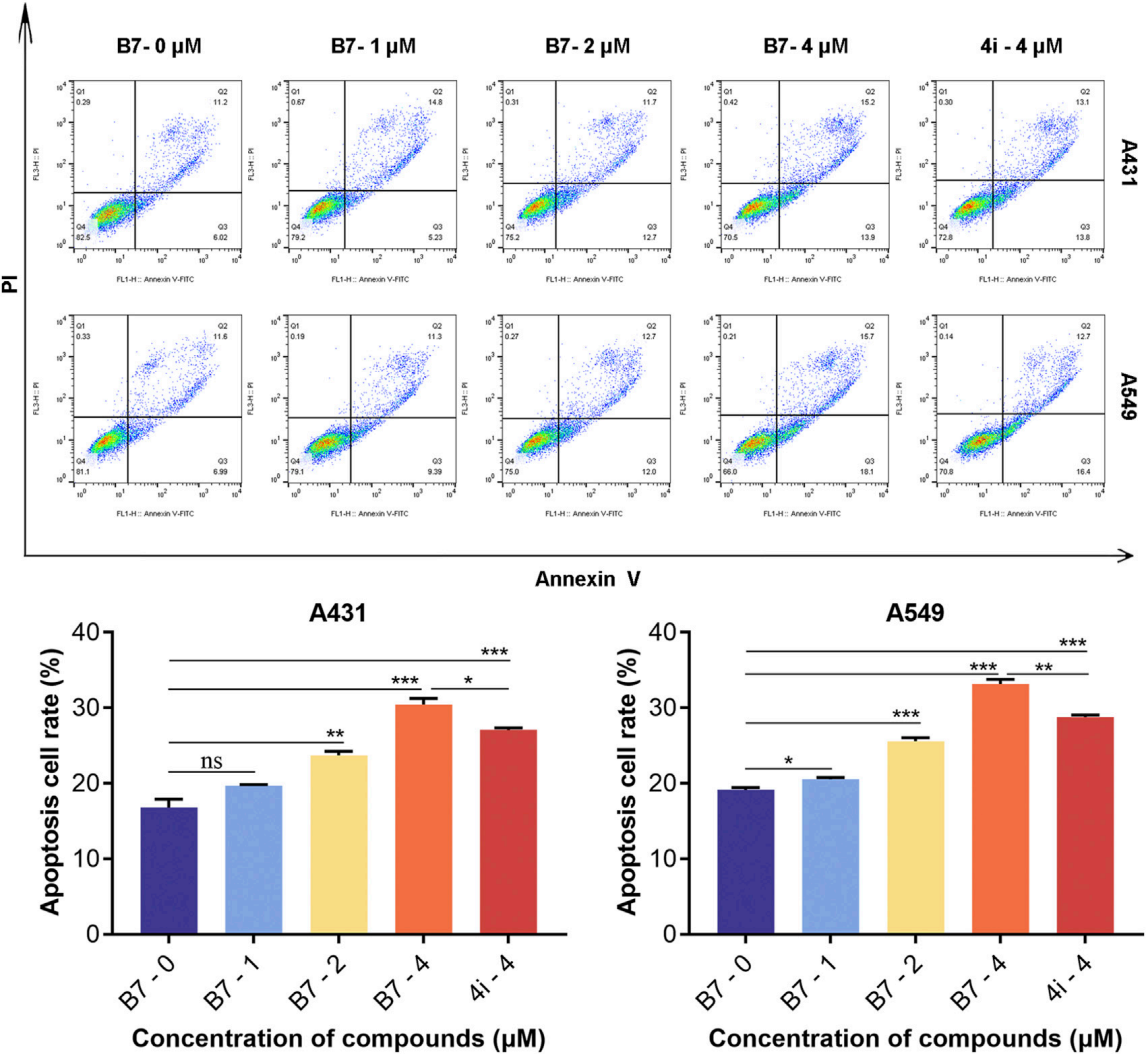
Apoptosis of A431 and A549 cells treated with B7 or 4i. Statistical significance differences were set to *p* > 0.05 (ns), *p* ≤ 0.05 (*), *p* ≤ 0.01 (**) and *p* ≤ 0.001 (***).

In subsequent experiments, a concise mechanistic exploration was undertaken on the optimized compound **B7**. The objective was to examine whether **B7** induces apoptosis in A431 and A549 cells. Flow cytometry was employed to assess the impact of **B7** on cancer cell apoptosis 24 h post-treatment (Figure 3). The findings unveiled a gradual increase in the apoptosis rate of both A431 and A549 cells with the escalating concentration of **B7**. Notably, at a concentration of 4 μM, the pro-apoptotic effect of **B7** equaled that of the lead compound **4i** and, indeed, surpassed the efficacy of **4i**.

#### 3.2.4 B7 blocked the cycle of A431 and A549 cells

**FIGURE 4:**
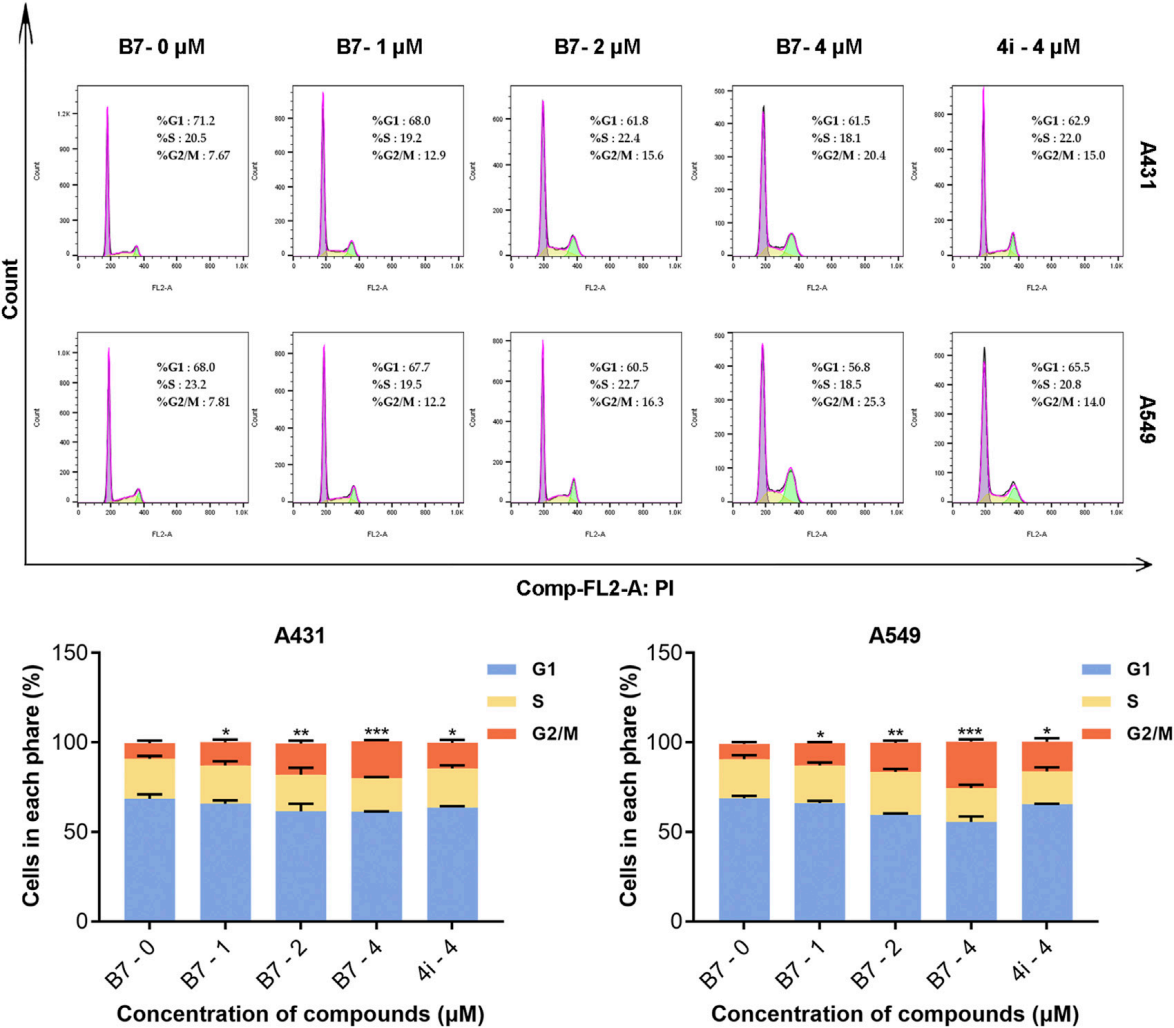
Cycle block of A431 and A549 cells treated with B7 or 4i. Statistical significance differences were set to *p* > 0.05 (ns), *p* ≤ 0.05 (*), *p* ≤ 0.01 (**) and *p* ≤ 0.001 (***).

In a parallel fashion, concentrations of 1, 2, and 4 μM were utilized for the application of flow cytometry to evaluate the cell cycle arrest induced by compound **B7** in A431 and A549 cells. As shown in Figure 4, compound **B7** and **4i** manifested a G2 phase cell cycle arrest effect on cancer cells, with **B7** exhibiting a noteworthy dose-dependent response. Notably, the cell cycle arrest induced by **B7** in both A431 and A549 cells surpassed that of **4i** at 4 μM.

#### 3.2.5 B7 inhibited the migration of A431 and A549 cells

**FIGURE 5:**
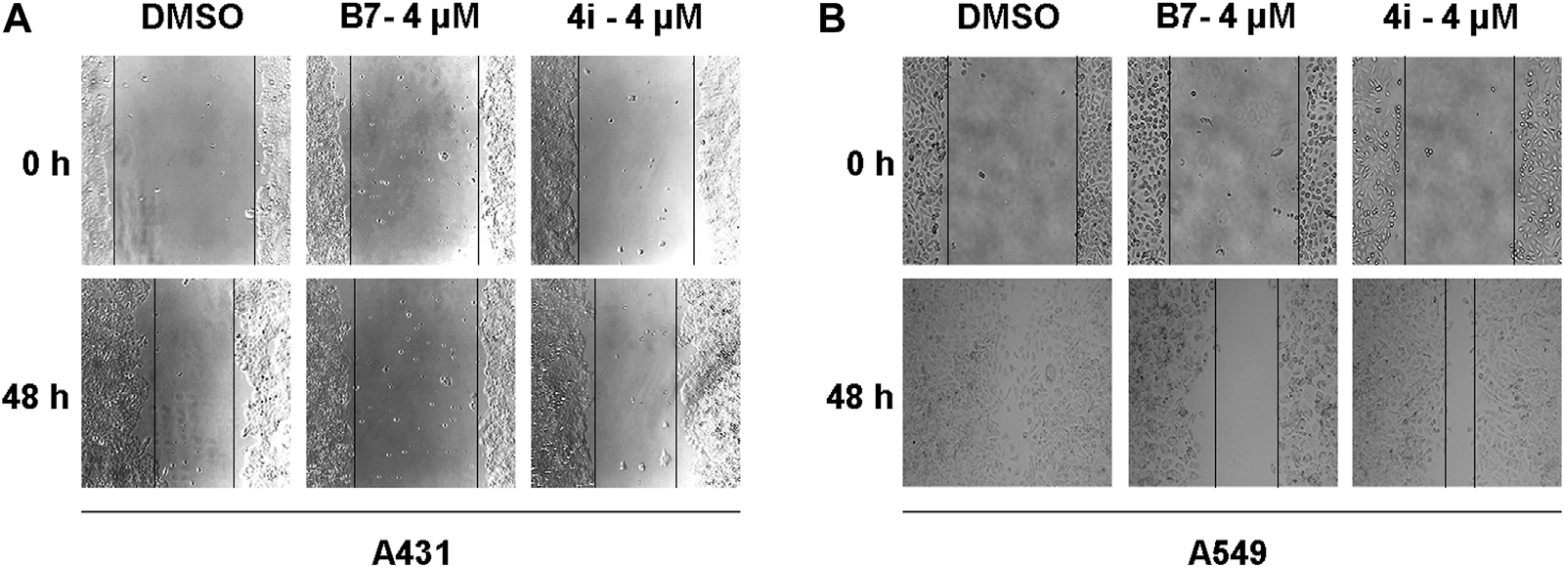
Migration of A431 and A549 cells treated with B7 or 4i. **(A)** The migration of A431 cells treated with B7 or 4i for 48 h; **(B)** The migration of A549 cells treated with B7 or 4i for 48 h.

Tumor cells are recognized for their capacity for unrestricted proliferation and resistance to apoptosis, accompanied by a proclivity for facile metastasis (Khan et al., 2023). Consequently, we employed the wound healing assay to assess the migratory inhibitory effects of **B7** and **4i** on A431 and A549 cells across 4 μM for 48 h (Figure 5). The findings indicated that in A431 cells, the migration inhibitory effect of **B7** slightly surpassed that of **4i**. However, as none of the groups exhibited complete healing, extending the experiment duration until full healing is observed could yield more precise conclusions. It is noteworthy that in A549 cells, scratches in the DMSO blank control group had fully healed, highlighting the stronger inhibitory effect of **B7** on cell migration compared to **4i**. In conclusion, **B7** demonstrated superior efficacy in inhibiting cancer cell migration.

#### 3.2.6 B7 inhibited the phosphorylation of AKT and ERK

**FIGURE 6:**
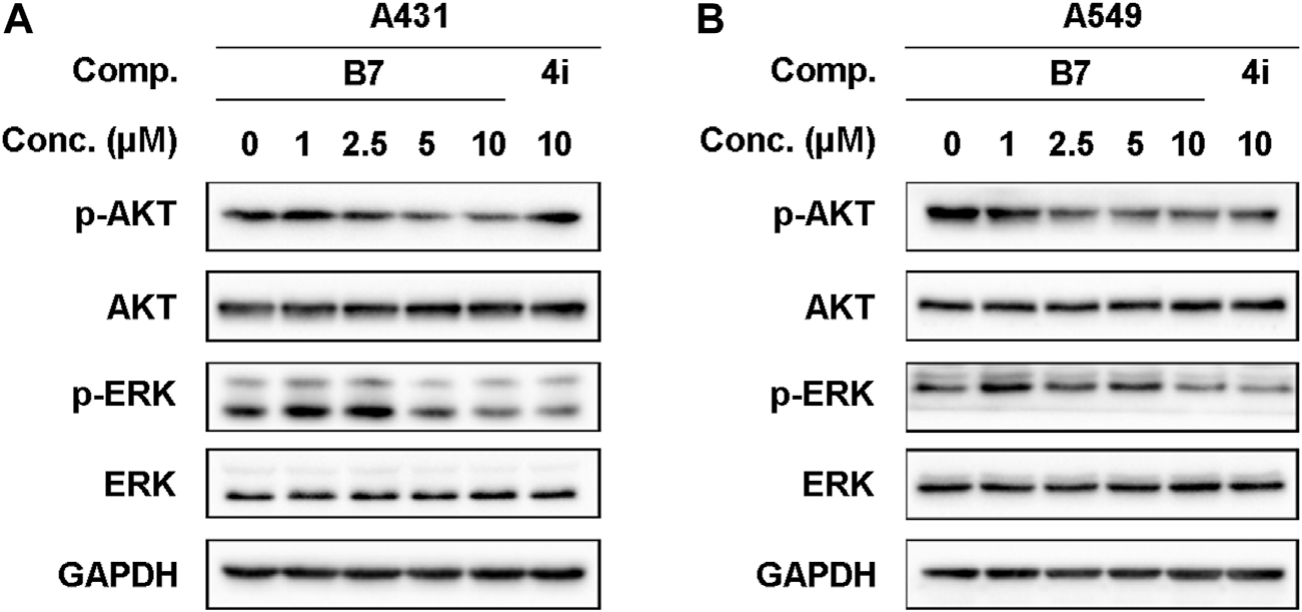
The protein expression levels of A431 and A549 cells treated with B7 or 4i. **(A)** The protein expression levels of A431 cells treated with B7 or 4i for 2 h; **(B)** The protein expression levels of A549 cells treated with B7 or 4i for 2 h.

The anticipated experimental findings underscored the significant anti-promotion, pro-apoptotic, cell cycle arrest effects, and anti-inflammatory properties of **B7** on A431 and A549 cells. A psychological inquiry into the pathways or key mechanisms through which **B7** manifests its remarkable anti-cancer and anti-inflammatory activity prompted investigation. To this end, we examined the activity of common anti-tumor receptor kinases, revealing that **B7** had minimal impact on them. Furthermore, employing Western blot analysis to scrutinize prevalent anti-tumor and anti-inflammatory signaling pathways, we observed that **B7** can markedly inhibit both AKT and ERK protein phosphorylation simultaneously, surpassing the inhibitory effect observed with **4i** (Figure 6). The synergistic effects of the AKT and ERK pathways in cancer therapy have garnered considerable attention. Some studies propose that co-inhibiting these two pathways may be more effective than inhibiting either one alone, as it can concurrently disrupt multiple biological processes in cells, thereby enhancing the overall effectiveness of treatment (Dimri et al., 2020; Wu et al., 2023). The concurrent inhibition of the AKT and ERK pathways by compound **B7** may elucidate its efficacy in both anti-inflammatory and anticancer capacities.

#### 3.2.7 In silico ADMET assessment of compound B7

The results of ADME and toxicity prediction were shown in Table 5. Based on the ADME prediction results, it could be seen that the physicochemical properties, ADME indexes and pharmacokinetic properties of B7 were within the permissible parameters (Ogbodo et al., 2023). From the toxicity prediction results, B7 had certain hepatotoxicity, but it was not irritating and corrosive to eyes and skin, and was not carcinogenic (Melo et al., 2022). Based on the above results, it was clear that B7 was a compound with better drug-like properties and deserved further studies.

**TABLE 5:** In silico ADMET properties of compound B7.

Properties	Prediction	Properties	Prediction
Physicochemical Properties		Pharmacokinetic	
Formula	C_14_H_10_ClN_3_O_2_S	GI absorption	High
Molecular weight	319.77 g/mol	BBB permeant	No
Num. heavy atoms	21	P-gp substrate	No
Num. arom. heavy atoms	15	CYP1A2 inhibitor	Yes
Fraction Csp3	0.07	CYP2C19 inhibitor	Yes
Num. rotatable bonds	4	CYP2C9 inhibitor	Yes
Num. H-bond acceptors	3	CYP2D6 inhibitor	No
Num. H-bond donors	1	CYP3A4 inhibitor	No
Molar Refractivity	87.24	Druglikeness	
TPSA	98.98 Å^2^	Lipinski	Yes; 0 violation
Lipophilicity		Ghose	Yes
Log *P*o/w (iLOGP)	2.52	Veber	Yes
Log *P*o/w (XLOGP)	4.65	Egan	Yes
Log *P*o/w (WLOGP)	4.13	Muegge	Yes
Log *P*o/w (MLOGP)	3.33	Bioavailability Score	0.55
Log *P*o/w (SILICOS-IT)	2.64	Toxicity	
Consensus Log *P*o/w	3.45	Carcinogenicity	-
Water Solubility		Eye corrosion/irritation	-
Log *S* (ESOL)	−5.02	Skin corrosion/irritation	-
Solubility	3.08e-03 mg/mL	Hepatotoxicity	+
Class	Moderately soluble	Acute oral toxicity (c)	ш

Legend: + (toxic); - (non-toxic); acute oral toxicity (c) level category—category I and II (toxic compound) and category III and IV (non-toxic compound), based on U.S. Environmental Protection Agency (EPA) criteria (Melo et al., 2022).

## 4 Discussion

In this study, a series of novel benzothiazole derivatives was synthesized, and their anti-proliferative and anti-inflammatory activities were systematically assessed in cellular contexts. The results delineate variable inhibitory effects of these compounds on the A431, A549, and H1299 cell lines. Notably, compound **B7** exhibits superior inhibitory effects across all three cancer cell lines. Furthermore, these compounds demonstrate a capacity to downregulate the expression of inflammatory factors IL-6 and TNF-α to varying extents. Specifically, compounds **B7** and **D2** significantly suppress the expression of IL-6 and TNF-α.

In light of the anti-tumor and anti-inflammatory activities of the target compounds, we conducted a comprehensive Structure-Activity Relationship (SAR) analysis on a novel series of 2-aminobenzothiazole compounds. The A-series compounds demonstrated inhibition rates below 50% for A549, A431, and H1299 cells, whereas the B, C, and D-series exhibited pronounced inhibitory effects on these cancer cells. It can be deduced that the benzylamine moiety, tethered to the benzothiazole ring in this compound class, serves as an indispensable pharmacophore and a pivotal pharmacophoric group. This necessity arises from the requirement for a larger substituent at this position to occupy, thereby promoting a more stable conformation. Upon comparison of the bioactivity between B-series and C-series compounds, it was observed that the overall bioactivity of B-series compounds surpasses that of the C-series. This observation implies that the introduction of a carbonyl group to form an amide bond did not yield an enhancement in anti-tumor and anti-inflammatory activities. Further scrutiny of various substituents on the benzothiazole ring unveiled a notable increase in bioactivity when chlorine is situated at the 6th position on the benzothiazole ring, in contrast to fluorine substitution at the 5th position. Exploring additional substituents on benzothiazole may hold promise for optimizing the activity of these compounds. The compound D2 exhibits significant advantages in anti-inflammatory aspects, which may be related to its unique bisubstituted structure, worthy of further study.

Additionally, the presence of a nitro group on compound **B7** has captured our attention. The nitro group is a common and distinctive functional group in medicinal chemistry, prevalent in various classes of drugs including anticancer agents, antibiotics, antituberculosis medications, antiparasitic agents, sedatives, insecticides, and herbicides. This moiety exhibits a potent electron-withdrawing capacity, leading to the formation of localized electron-deficient sites within the molecule. It engages with nucleophilic reagents present in biological systems such as proteins, amino acids, nucleic acids, and enzymes through processes like nucleophilic addition, electron transfer, or complexation. Consequently, compounds containing nitro groups have been extensively studied in medicinal chemistry research. For instance, Nifurtimox (Rolon et al., 2022; Xin et al., 2022; Eslin et al., 2023), utilized in the treatment of Chagas disease and recurrent neuroblastoma, and Venetoclax (Li et al., 2024a), a Bcl-2 inhibitor used for managing chronic lymphocytic leukemia, both feature nitro structures. However, it is imperative to acknowledge that drugs incorporating nitro groups often elicit severe adverse reactions and toxicity, including carcinogenicity, hepatotoxicity, mutagenicity, and bone marrow suppression. Consequently, the nitro group is frequently considered a red flag structural motif, which somewhat impedes the exploration of its therapeutic potential. Nevertheless, in our study, the investigated compounds demonstrated negligible inhibitory effects on Beas-2b cells, indicating a lack of cytotoxicity *in vitro*. However, further investigation is warranted to evaluate their toxicity *in vivo*.

A key mechanism underlying **B7**’s anticancer efficacy appears to be its dual inhibition of the AKT and ERK signaling pathways. The AKT and ERK cascades play pivotal roles in tumor cell proliferation, survival, and metastasis (Chen et al., 2023; Cui et al., 2023). While previous benzothiazole-based inhibitors have targeted single pathways, **B7** is unique in concurrently suppressing both AKT and ERK phosphorylation. This dual targeting likely contributes to **B7**’s potent induction of apoptosis, cell cycle arrest, and inhibition of cell migration in our experiments.

Additionally, **B7** significantly reduces the production of the inflammatory cytokines IL-6 and TNF-α. As highlighted in the Introduction, chronic inflammation driven by these factors can create microenvironments conducive to tumor growth and progression. Therefore, **B7**’s anti-inflammatory properties may further augment its anticancer effects.

Future studies should focus on elucidating the precise molecular interactions enabling **B7**’s dual pathway inhibition. Testing **B7**’s efficacy in animal models will also be crucial to assess its potential for clinical translation. Limitations of this initial study include the narrow range of cancer cell lines examined. Expanding the panel of cell lines could provide further insights into **B7**’s spectrum of anticancer activity.

## 5 Conclusion

In summary, this study has innovatively developed a series of benzothiazole derivatives, with a focus on compound **B7** due to its notable dual anticancer and anti-inflammatory activities. **B7** stands out for its ability to significantly reduce cancer cell proliferation in A431, A549, and H1299 cell lines and lower the levels of inflammatory cytokines IL-6 and TNF-α. These results position B7 as a promising candidate for dual-action cancer therapy. The study’s mechanistic exploration, highlighting **B7**’s simultaneous inhibition of the AKT and ERK pathways, offers a novel strategy for addressing both the survival mechanisms of tumor cells and the inflammatory milieu facilitating cancer progression.

## Data Availability

The original contributions presented in the study are included in the article/Supplementary Material, further inquiries can be directed to the corresponding authors.
