# Supplementary material for: Synthesis and biological evaluation of novel benzothiazole derivatives as potential anticancer and antiinflammatory agents
Source: Front Chem. 2024 Mar 18;12:1384301. doi: 10.3389/fchem.2024.1384301 (PMC10982501; doi:10.3389/fchem.2024.1384301)
Supplement: Supplementary file 1 [file DataSheet1.pdf]

Supplementary Material

1 Supplementary Figures

1.1 Spectrums of the compounds A1-A8

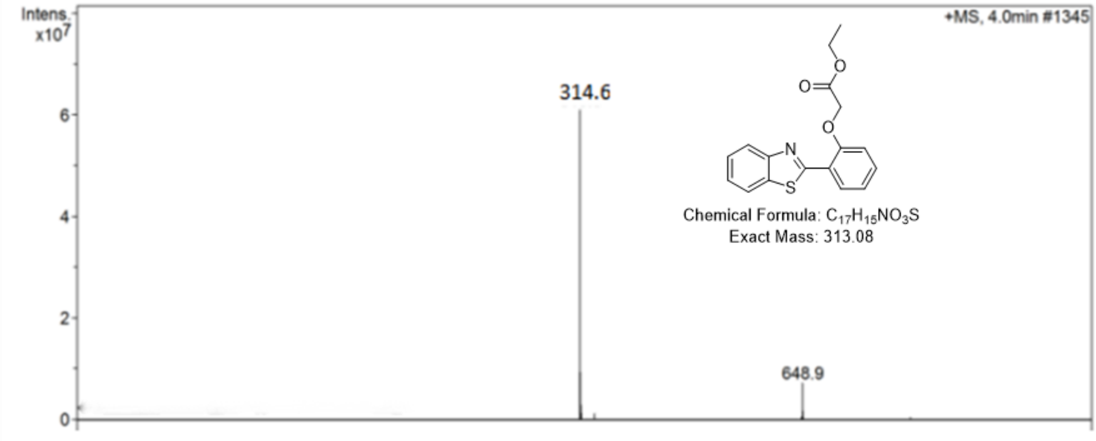

Supplementary Figure 1. Mass spectra of the compound **A1**.

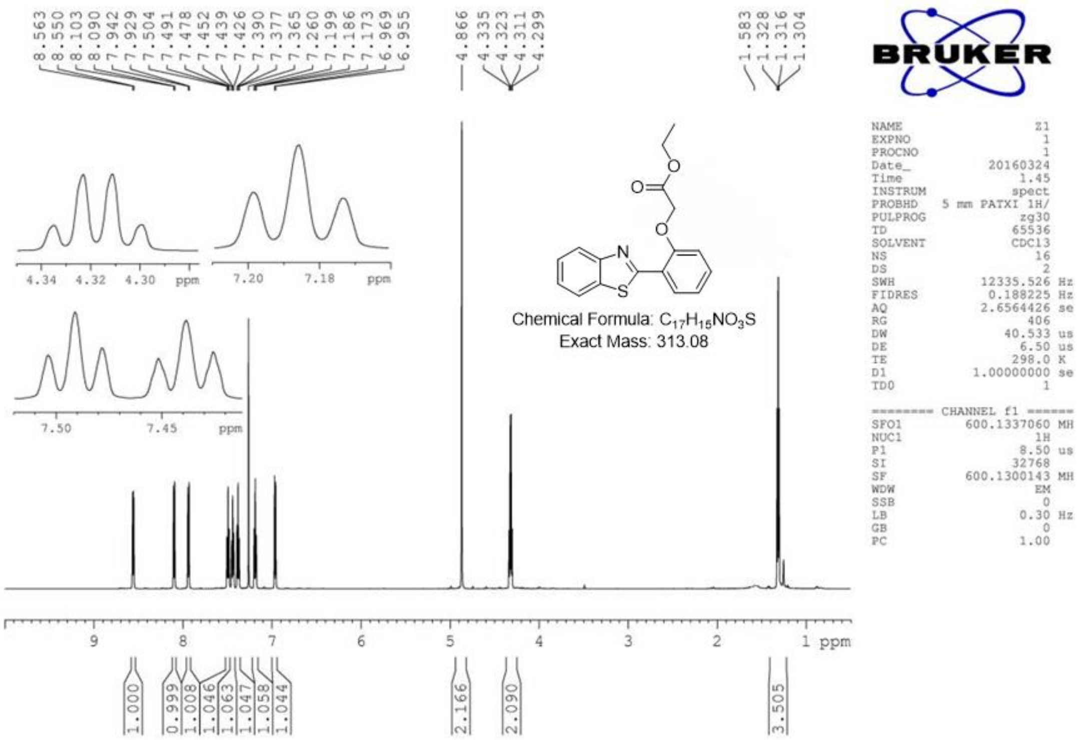

Supplementary Figure 2. ^{1}H NMR spectra of the compound **A1**.

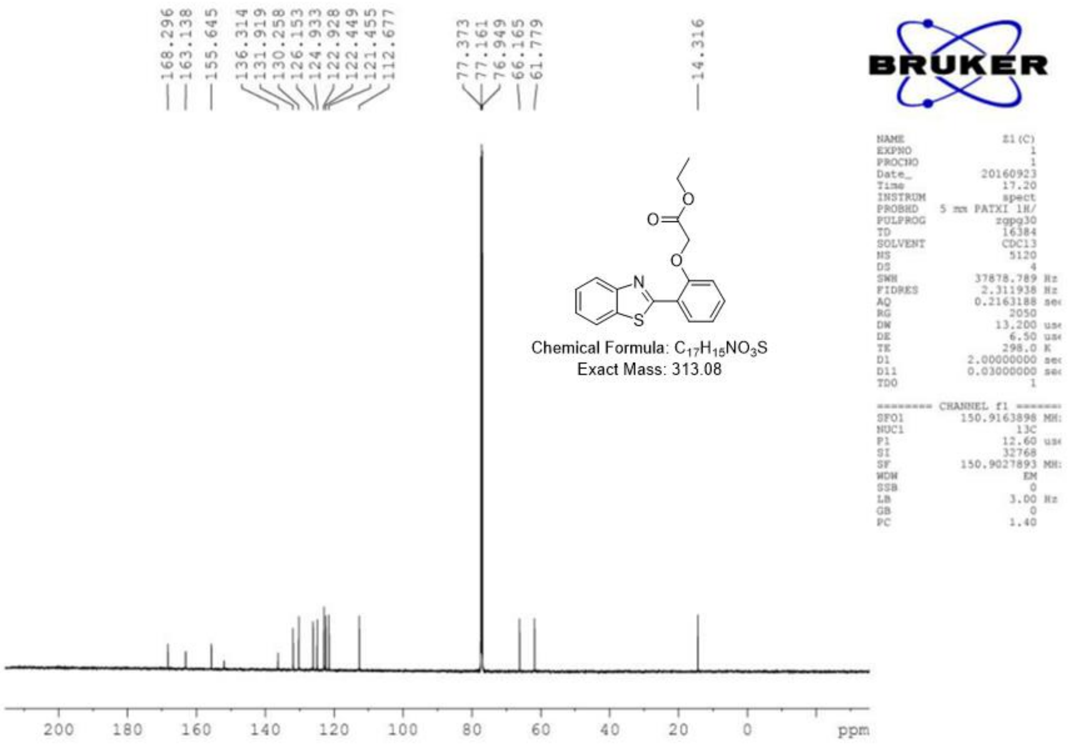

Supplementary Figure 3. ^{13}C NMR spectra of the compound **A1**.

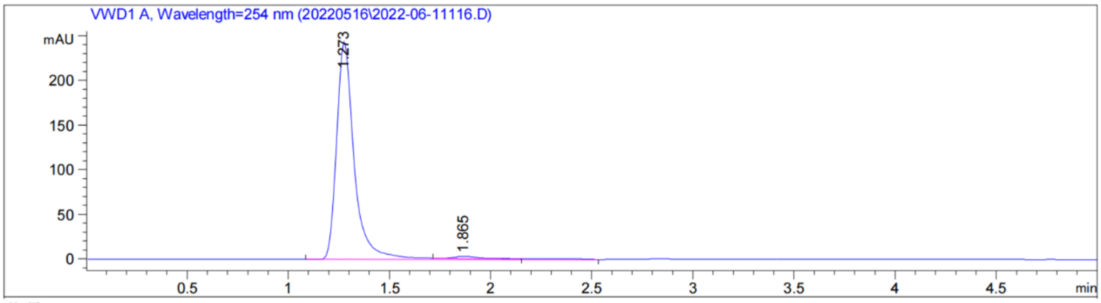

信号 1: VWD1 A, Wavelength=254 nm

峰 #	保留时间 [min]	类型	峰宽 [min]	峰面积 [mAU*s]	峰高 [mAU]	峰面积 %
1	1.273	BV R	0.0948	1520.11487	243.68446	98.3716
2	1.865	VV E	0.1418	25.16327	2.53583	1.6284
总量 :				1545.27814	246.22029	

Supplementary Figure 4. HPLC of the compound **A1**.

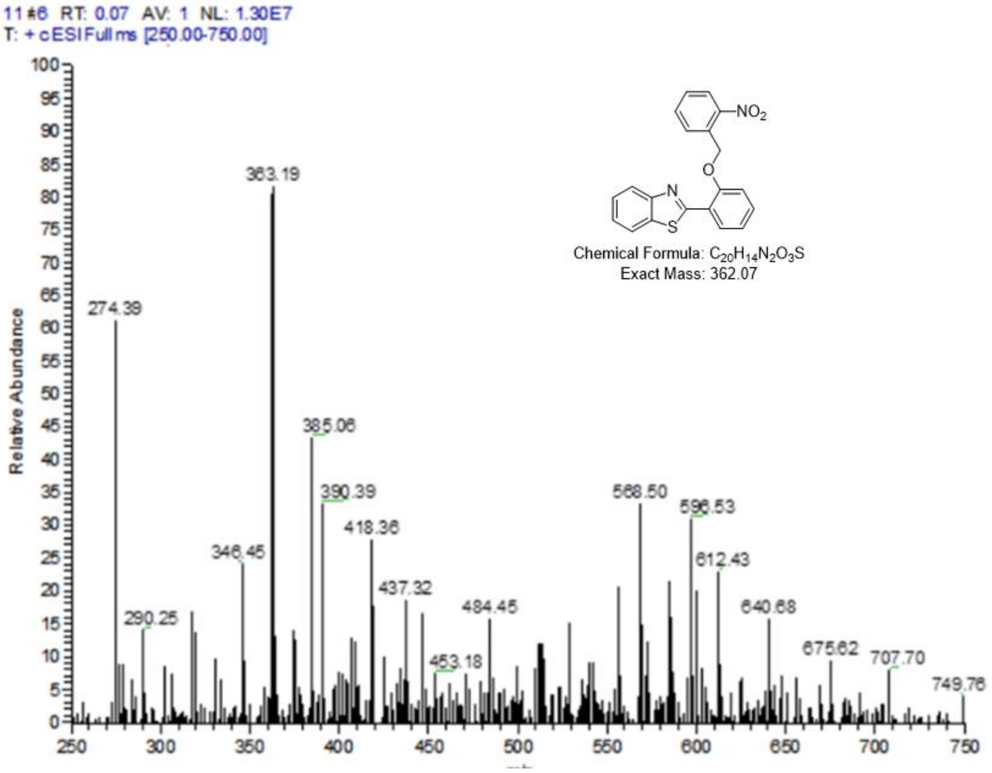

Supplementary Figure 5. Mass spectra of the compound **A2**.

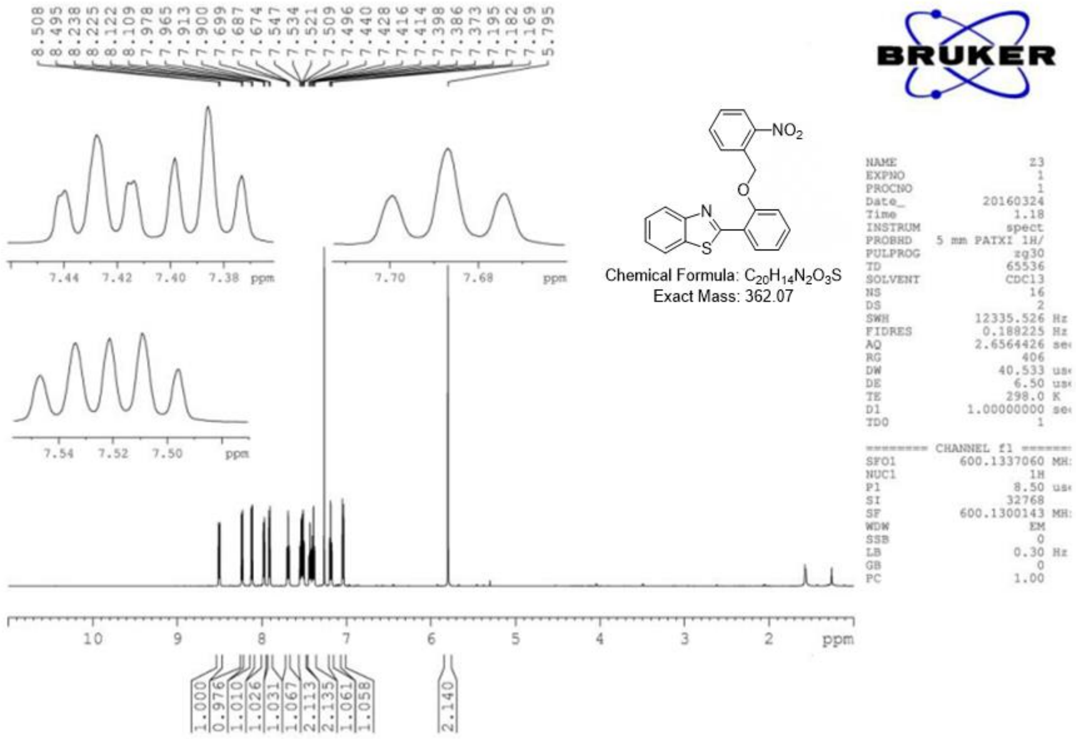

Supplementary Figure 6. ^{1}H NMR spectra of the compound **A2**.

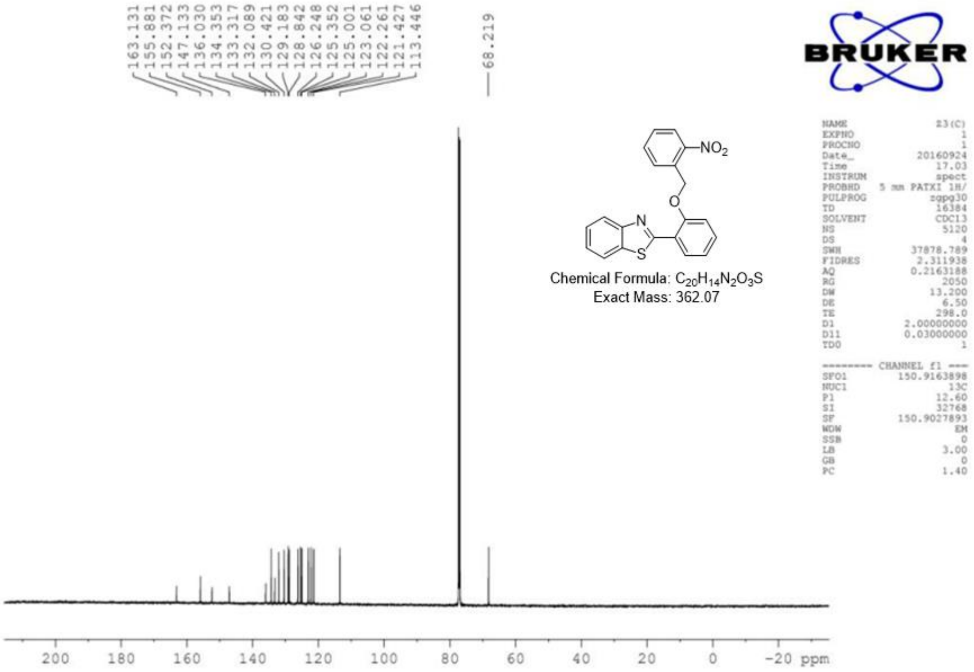

Supplementary Figure 7. ^{13}C NMR spectra of the compound **A2**.

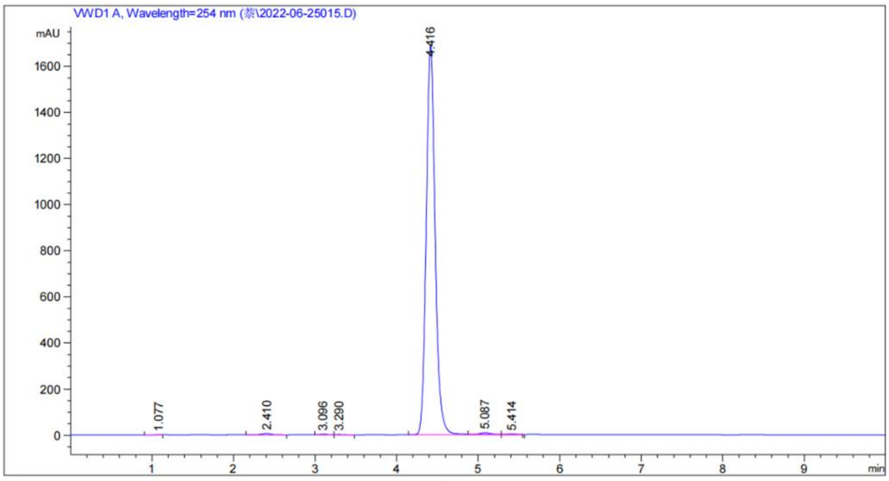

信号 1: VWD1 A, Wavelength=254 nm

峰 #	保留时间 [min]	类型	峰宽 [min]	峰面积 [mAU*s]	峰高 [mAU]	峰面积 %
1	1.077	BV	0.0675	8.40065	1.76508	0.0645
2	2.410	VB	0.1398	52.67533	5.59896	0.4046
3	3.096	VV	0.1188	21.37686	2.49791	0.1642
4	3.290	VB	0.0903	11.00731	1.80069	0.0846
5	4.416	BV R	0.1168	1.28363e4	1686.08447	98.6036
6	5.087	VV E	0.1419	67.00337	7.24184	0.5147
7	5.414	VB E	0.1348	21.32376	2.42075	0.1638
总量 :				1.30181e4	1707.40970	

Supplementary Figure 8. HPLC of the compound **A2**.

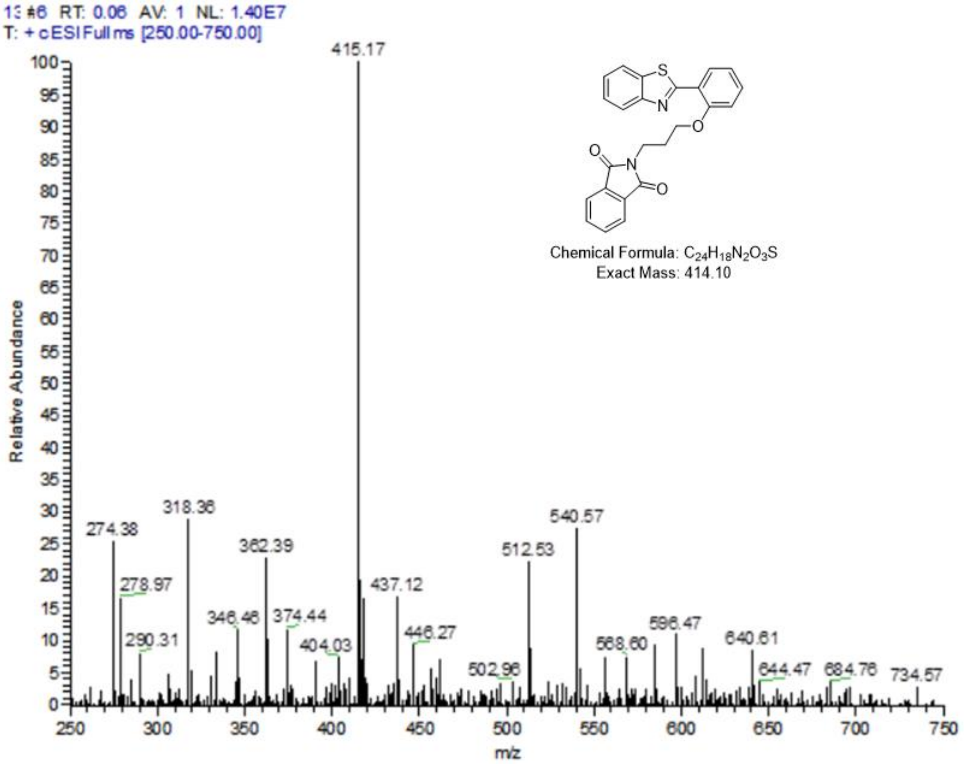

Supplementary Figure 9. Mass spectra of the compound **A3**.

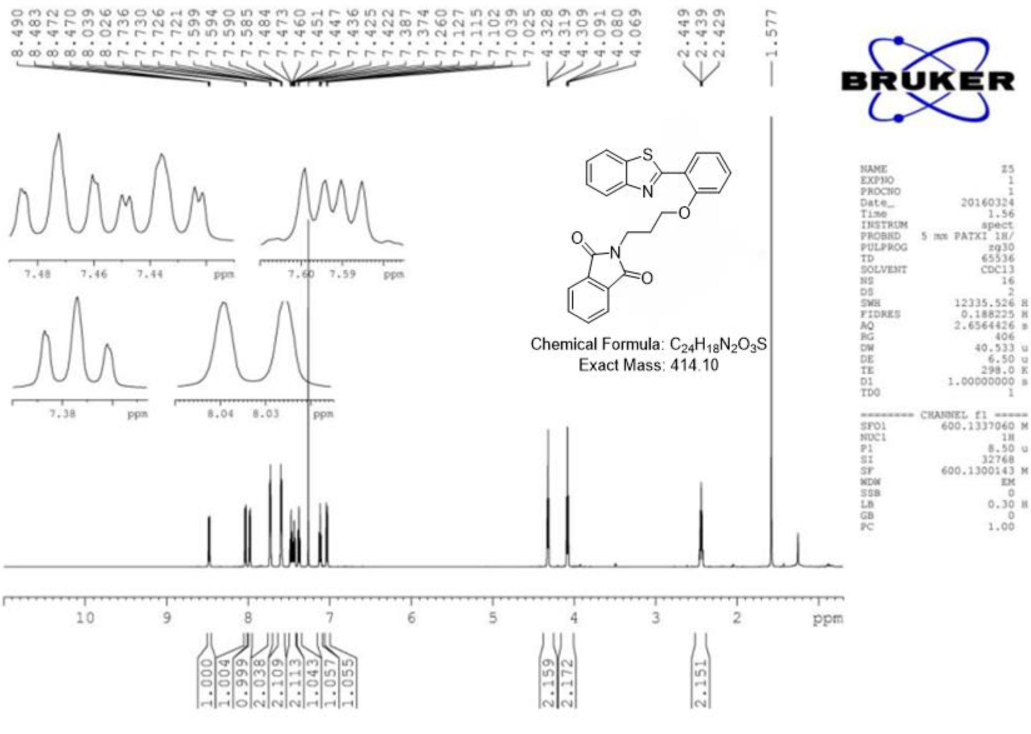

Supplementary Figure 10. ^{1}H NMR spectra of the compound **A3**.

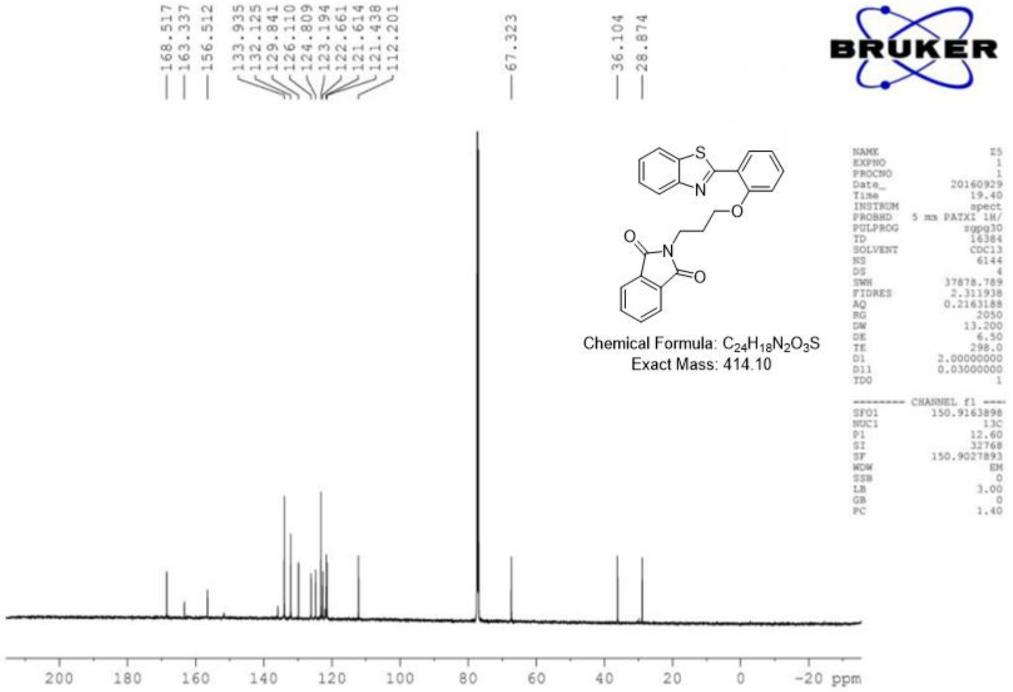

Supplementary Figure 11. ^{13}C NMR spectra of the compound **A3**.

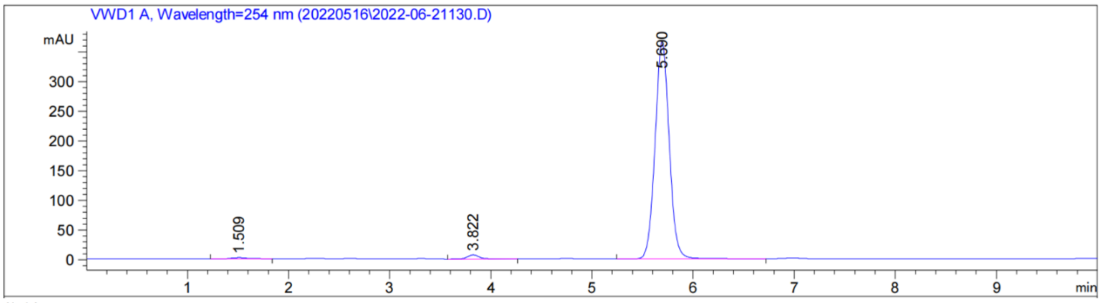

信号 1: VWD1 A, Wavelength=254 nm

峰 #	保留时间 [min]	类型	峰宽 [min]	峰面积 [mAU*s]	峰高 [mAU]	峰面积 %
1	1.509	BB	0.2049	30.24724	2.22123	0.8273
2	3.822	BB	0.1310	61.46513	6.96001	1.6811
3	5.690	BB	0.1518	3564.59229	365.41660	97.4917
总量 :				3656.30465	374.59784	

Supplementary Figure 12. HPLC of the compound **A3**.

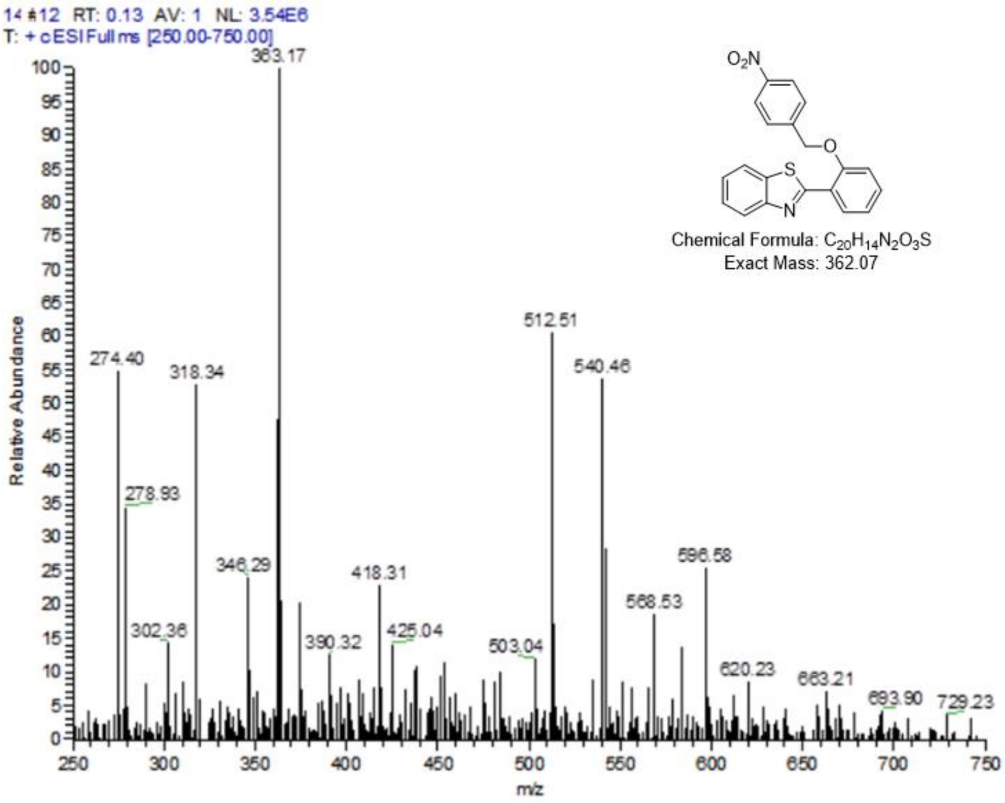

Supplementary Figure 13. Mass spectra of the compound **A4**.

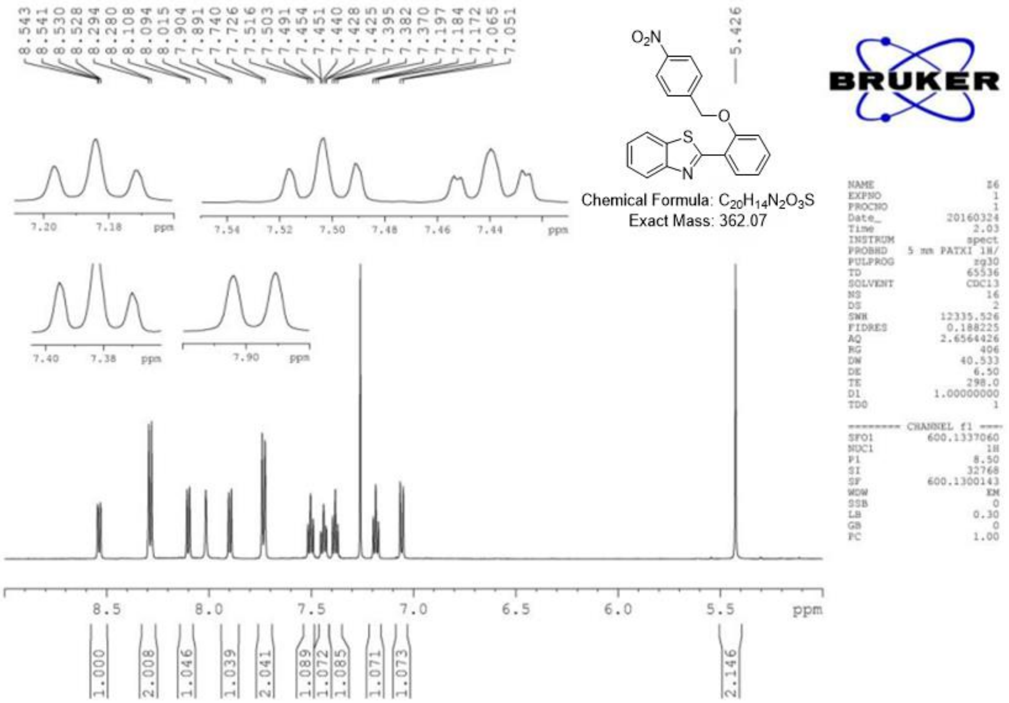

Supplementary Figure 14. ^{1}H NMR spectra of the compound **A4**.

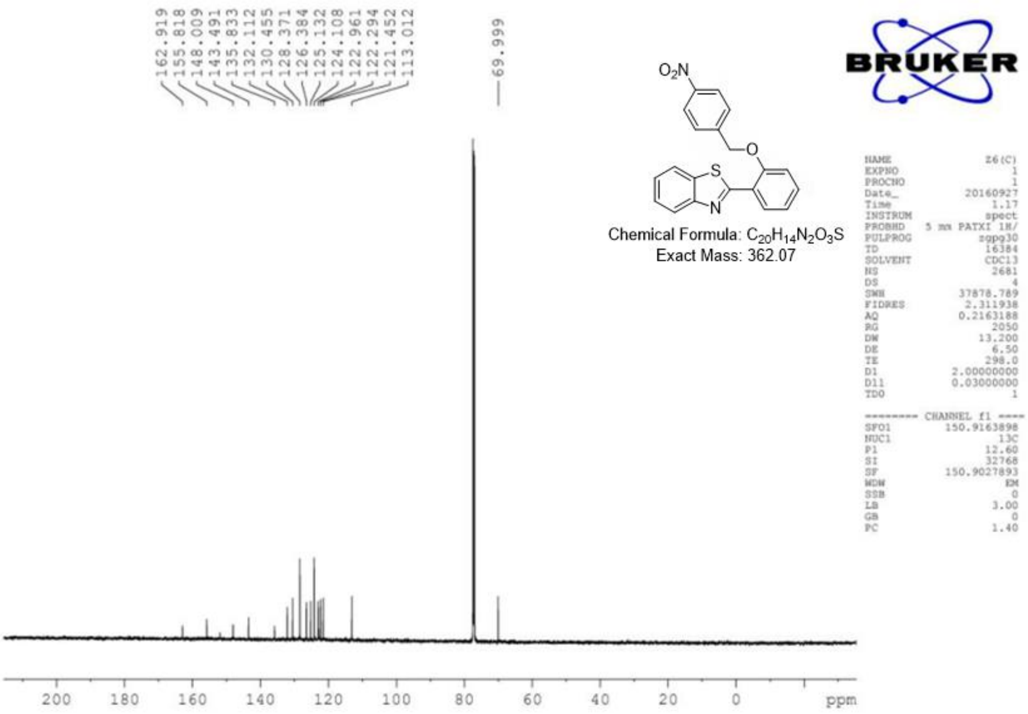

Supplementary Figure 15. ^{13}C NMR spectra of the compound **A4**.

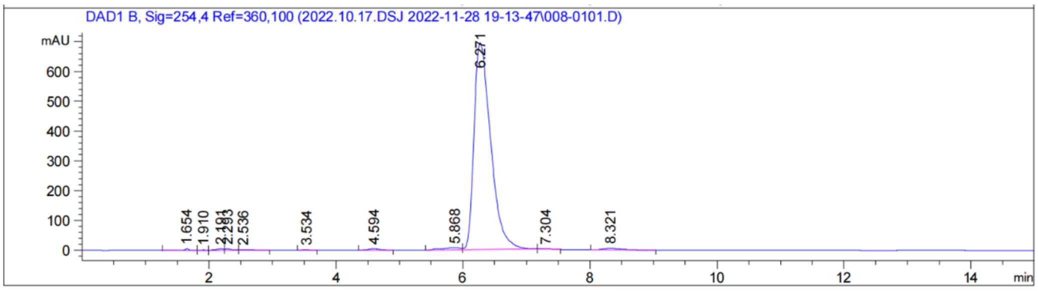

信号 2: DAD1 B, Sig=254,4 Ref=360,100

峰 #	保留时间 [min]	类型	峰宽 [min]	峰面积 [mAU*s]	峰高 [mAU]	峰面积 %
1	1.654	BV	0.0839	29.04776	4.85146	0.2208
2	1.910	VB	0.0958	11.12329	1.51289	0.0845
3	2.191	BV	0.1177	43.24784	5.05911	0.3287
4	2.293	VV	0.1107	39.32341	4.84372	0.2989
5	2.536	VB	0.2009	34.83689	2.22255	0.2648
6	3.534	VB	0.1323	15.61505	1.68195	0.1187
7	4.594	BB	0.1759	57.19411	4.51312	0.4347
8	5.868	BV	0.3156	142.58095	6.45640	1.0837
9	6.271	VB	0.2830	1.26612e4	693.51752	96.2290
10	7.304	BB	0.1755	12.64153	1.13891	0.0961
11	8.321	BB	0.3427	110.55927	4.87307	0.8403
总量 :				1.31574e4	730.67069	

Supplementary Figure 16. HPLC of the compound **A4**.

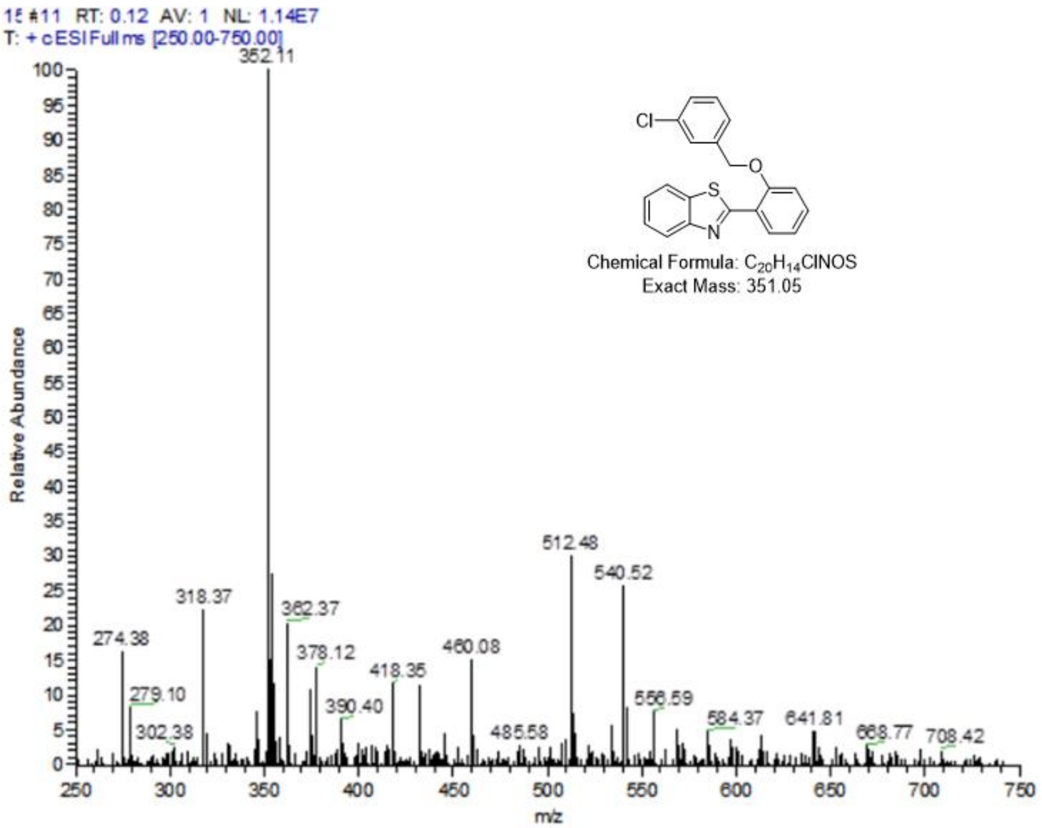

Supplementary Figure 17. Mass spectra of the compound **A5**.

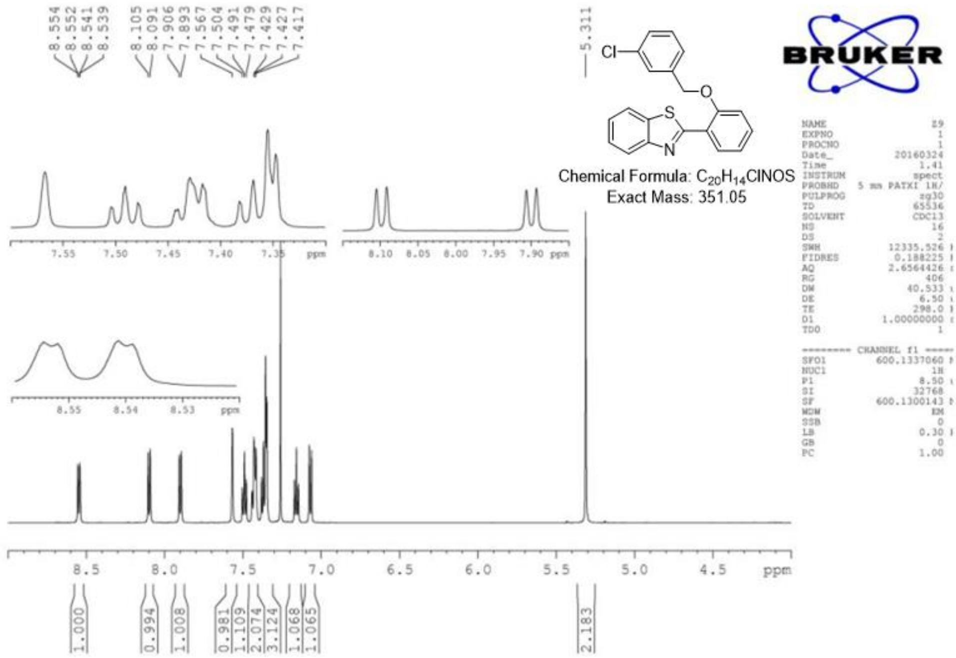

Supplementary Figure 18. ^{1}H NMR spectra of the compound **A5**.

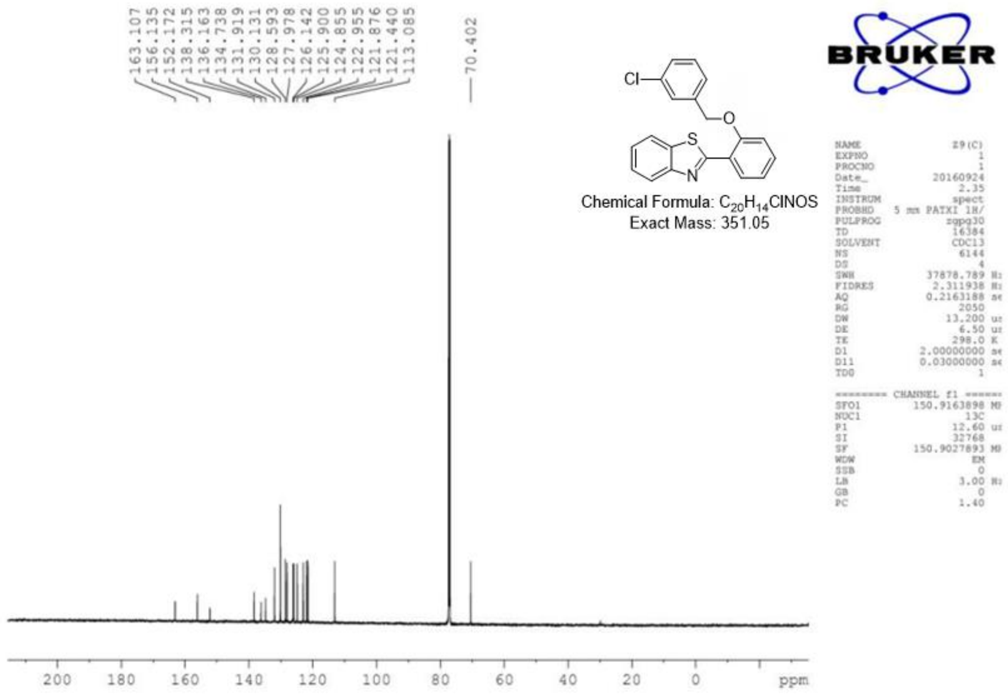

Supplementary Figure 19. ^{13}C NMR spectra of the compound **A5**.

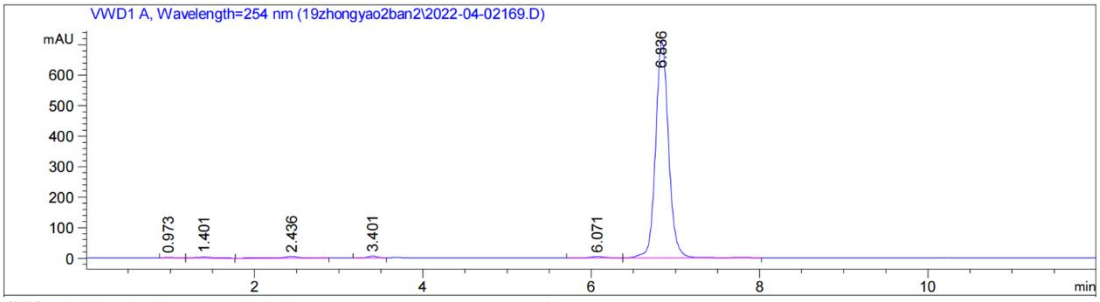

信号 1: VWD1 A, Wavelength=254 nm

峰 #	保留时间 [min]	类型	峰宽 [min]	峰面积 [mAU*s]	峰高 [mAU]	峰面积 %
1	0.973	VV	0.1504	20.67330	1.90885	0.2627
2	1.401	VB	0.1986	54.59683	3.69009	0.6938
3	2.436	BB	0.1783	70.54955	5.54883	0.8966
4	3.401	BV	0.1071	44.56075	6.41100	0.5663
5	6.071	BB	0.1528	46.51825	4.64743	0.5912
6	6.836	BV R	0.1654	7632.06494	710.30823	96.9895
总量 :				7868.96362	732.51443	

Supplementary Figure 20. HPLC of the compound **A5**.

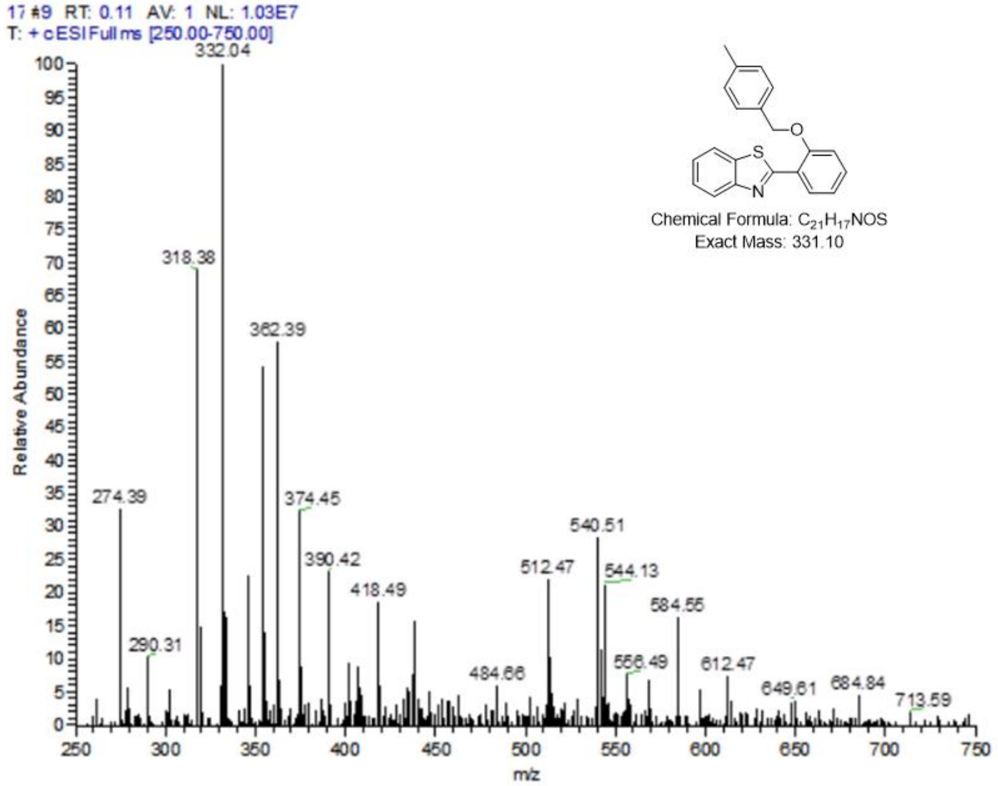

Supplementary Figure 21. Mass spectra of the compound **A6**.

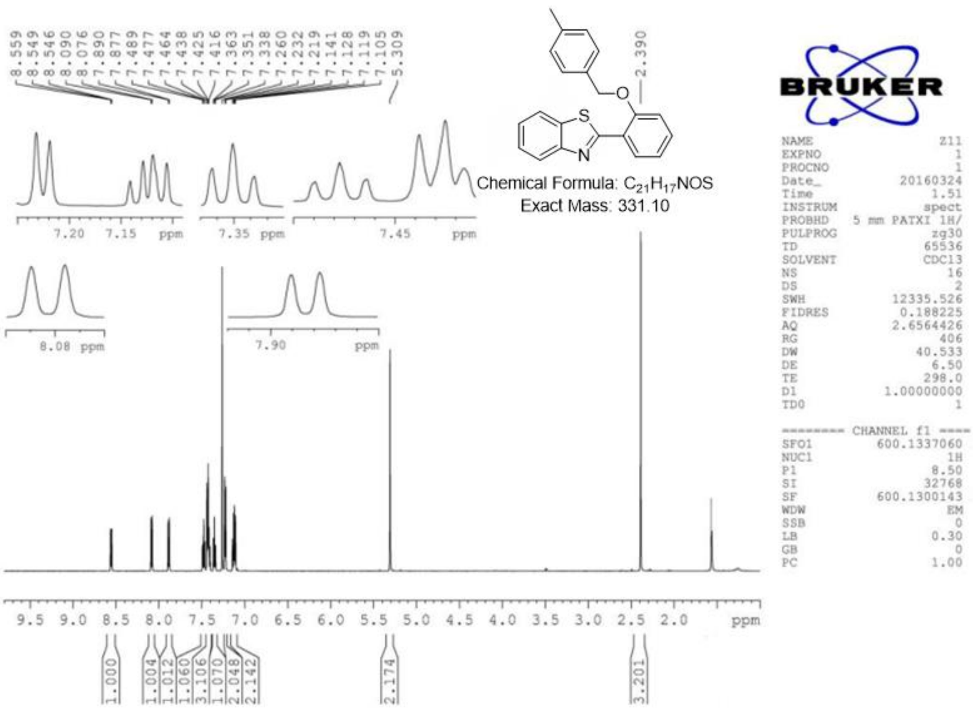

Supplementary Figure 22. ^{1}H NMR spectra of the compound **A6**.

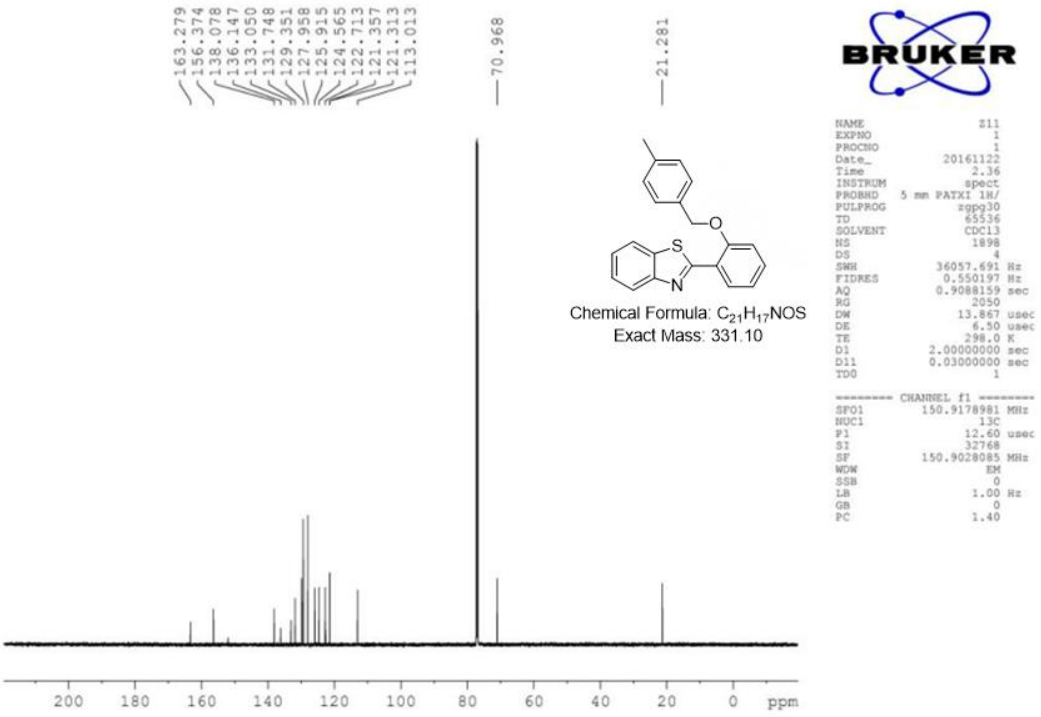

Supplementary Figure 23. ^{13}C NMR spectra of the compound **A6**.

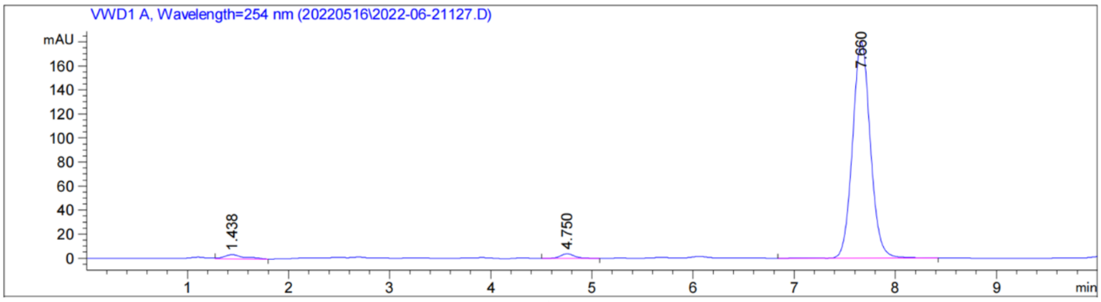

信号 1: VWD1 A, Wavelength=254 nm

峰 #	保留时间 [min]	类型	峰宽 [min]	峰面积 [mAU*s]	峰高 [mAU]	峰面积 %
1	1.438	VB	0.2005	46.44186	3.37575	2.0304
2	4.750	BB	0.1429	34.57592	3.70307	1.5116
3	7.660	VB R	0.1891	2206.31128	179.90427	96.4580
总量 :				2287.32905	186.98308	

Supplementary Figure 24. HPLC of the compound **A6**.

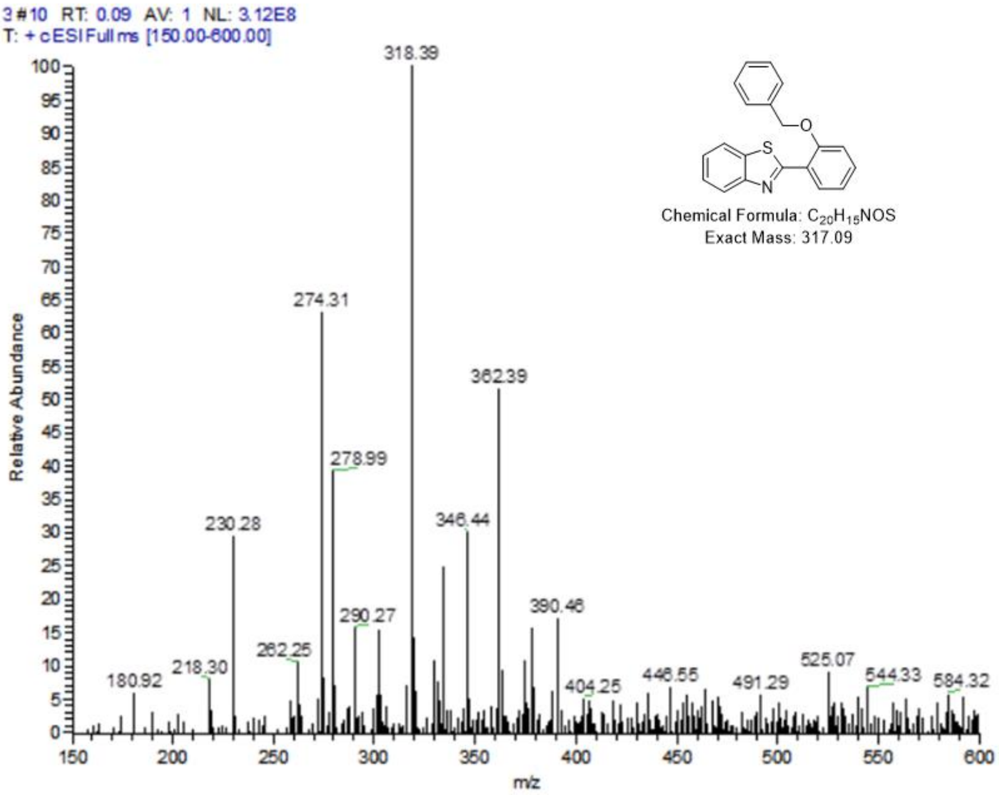

Supplementary Figure 25. Mass spectra of the compound **A7**.

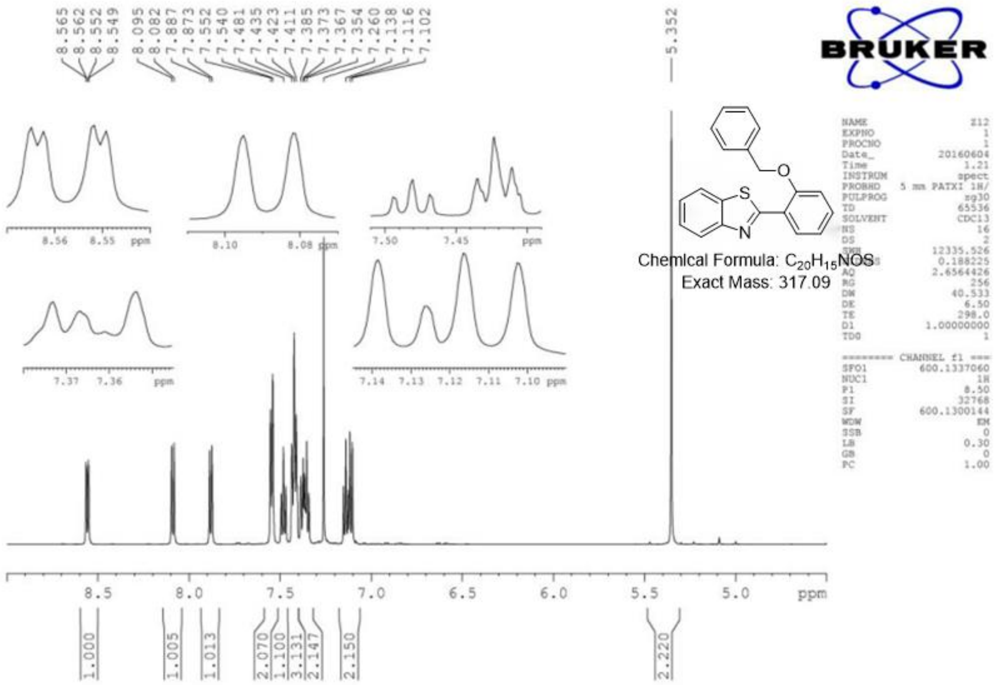

Supplementary Figure 26. ^{1}H NMR spectra of the compound **A7**.

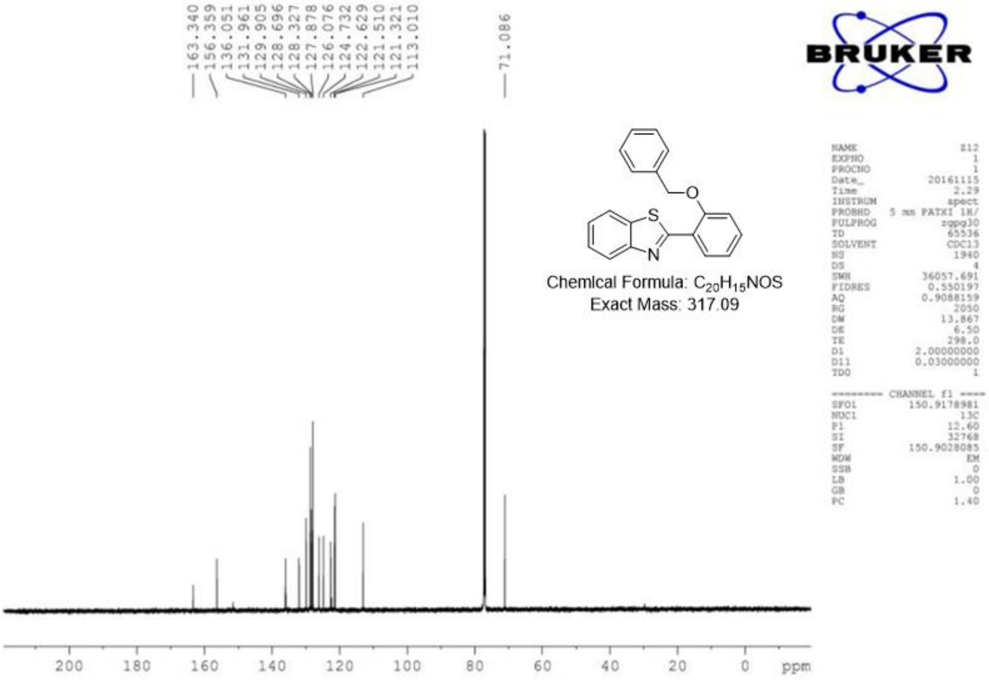

Supplementary Figure 27. ^{13}C NMR spectra of the compound **A7**.

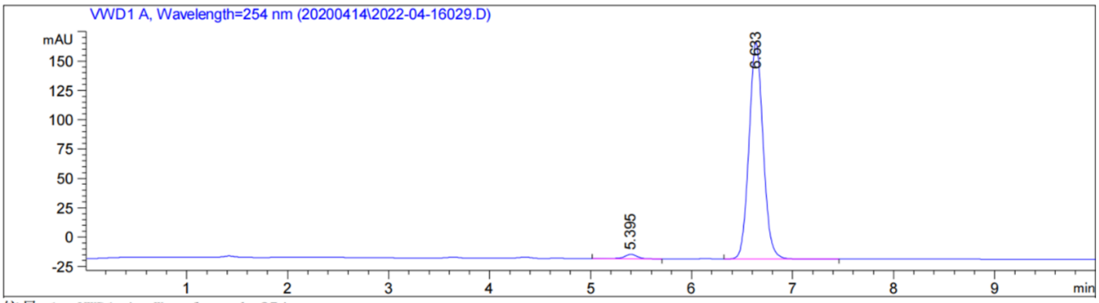

信号 1: VWD1 A, Wavelength=254 nm

峰 #	保留时间 [min]	类型	峰宽 [min]	峰面积 [mAU*s]	峰高 [mAU]	峰面积 %
1	5.395	BB	0.1419	35.44033	3.76353	1.9389
2	6.633	BB	0.1512	1792.38757	184.76604	98.0611

总量 : 1827.82790 188.52957

Supplementary Figure 28. HPLC of the compound **A7**.

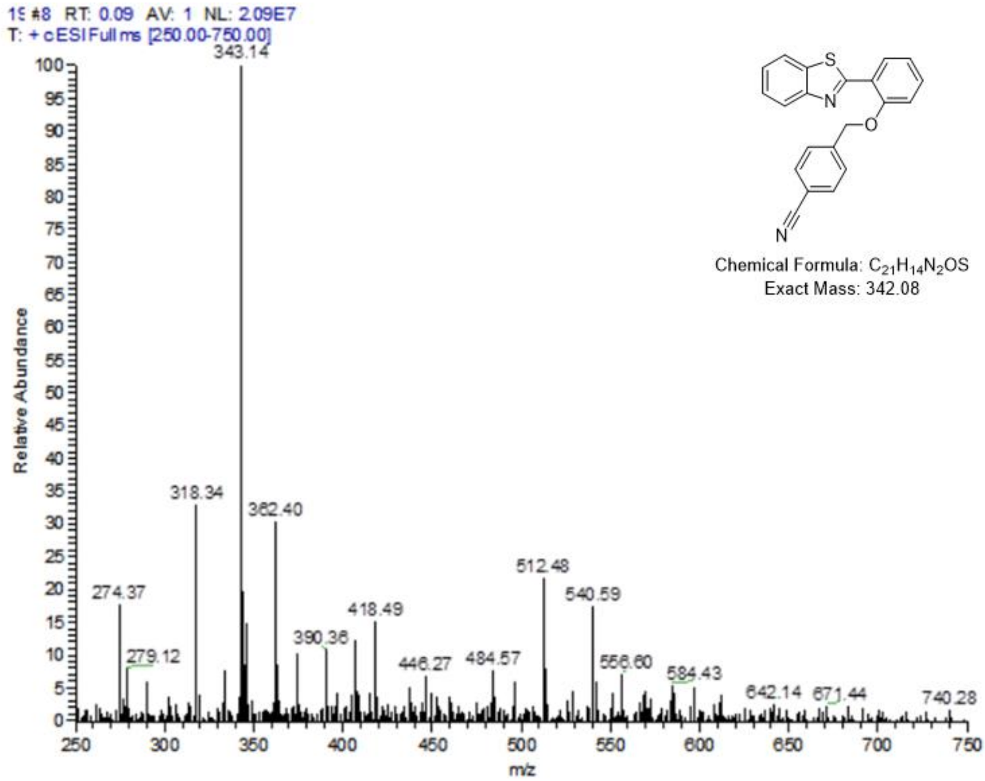

Supplementary Figure 29. Mass spectra of the compound **A8**.

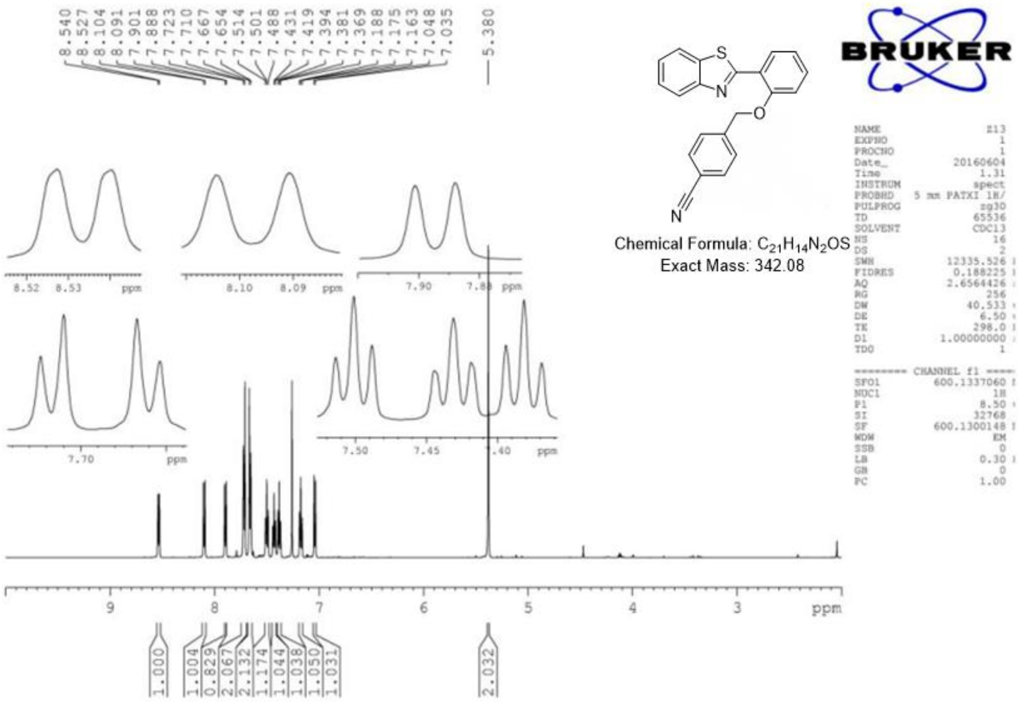

Supplementary Figure 30. ^{1}H NMR spectra of the compound **A8**.

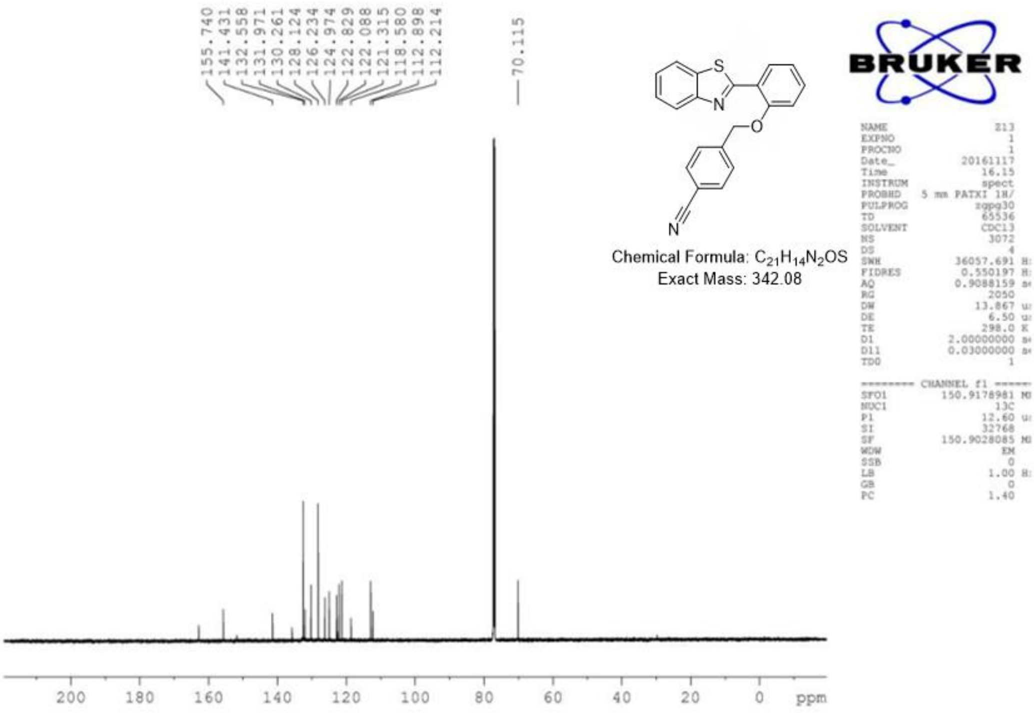

Supplementary Figure 31. ^{13}C NMR spectra of the compound **A8**.

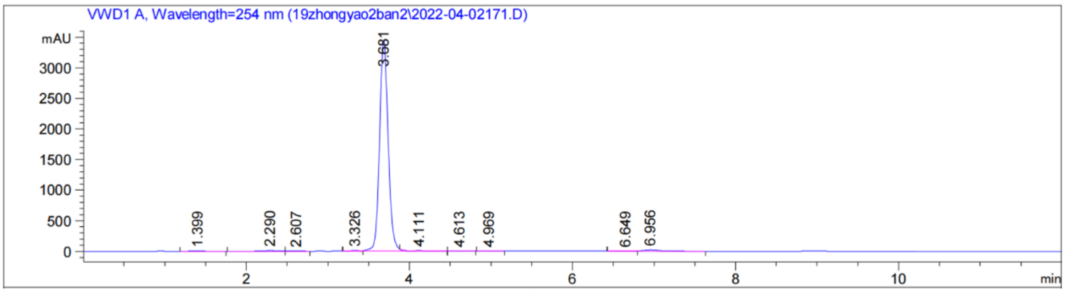

信号 1: VWD1 A, Wavelength=254 nm

峰 #	保留时间 [min]	类型	峰宽 [min]	峰面积 [mAU*s]	峰高 [mAU]	峰面积 %
1	1.399	VB	0.1925	50.27463	3.52215	0.1917
2	2.290	BV	0.1937	124.21664	9.55425	0.4737
3	2.607	VB	0.1245	54.69843	6.89834	0.2086
4	3.326	BV E	0.1009	68.90975	10.73108	0.2628
5	3.681	VV R	0.1143	2.54713e4	3442.91577	97.1428
6	4.111	VB E	0.1549	84.60328	7.77633	0.3227
7	4.613	BB	0.1225	31.12755	3.92652	0.1187
8	4.969	BB	0.1377	15.16656	1.73988	0.0578
9	6.649	BV E	0.1680	35.95943	3.38429	0.1371
10	6.956	VB R	0.1986	284.21329	21.72897	1.0839
总量 :				2.62204e4	3512.17757	

Supplementary Figure 32. HPLC of the compound **A8**.

1.2 Spectrums of the compounds B1-B8

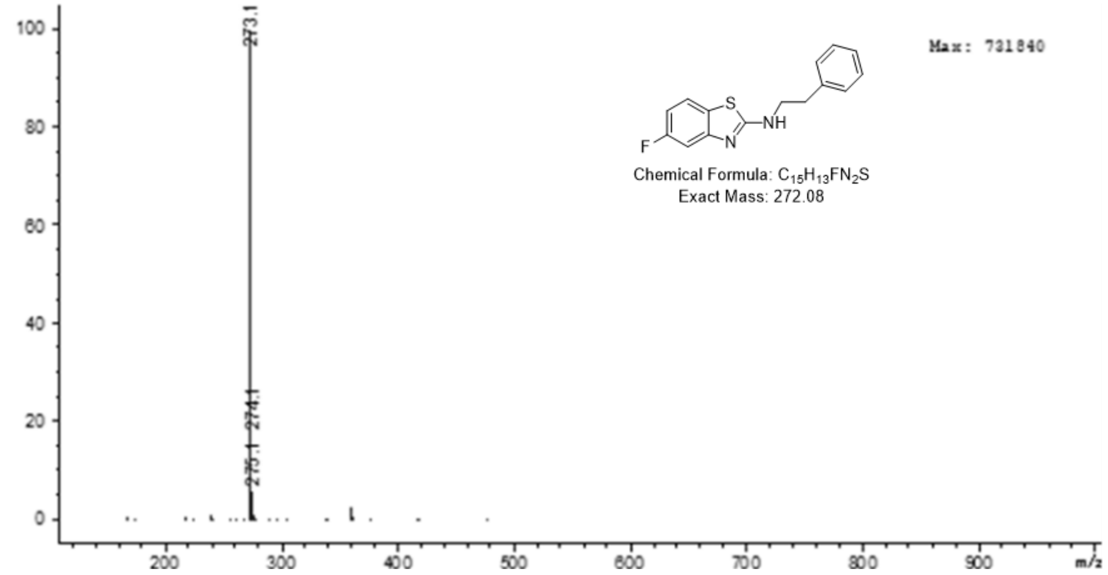

Supplementary Figure 33. Mass spectra of the compound **B1**.

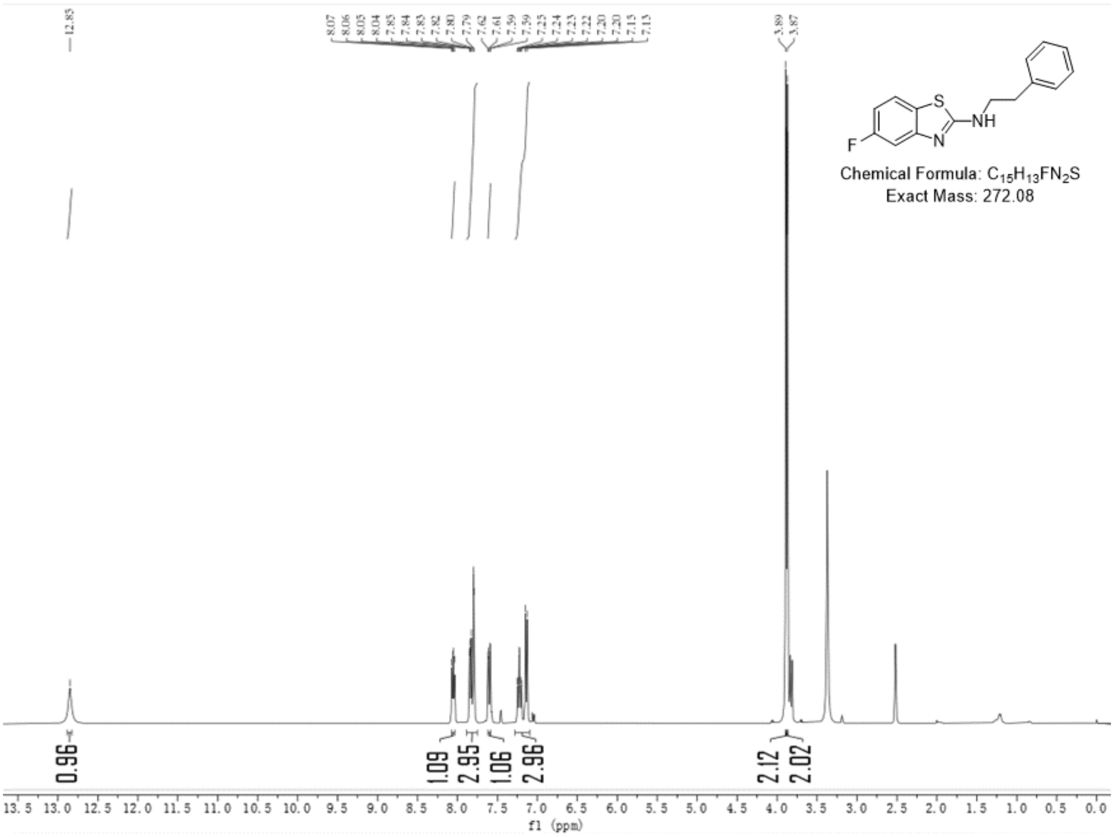

Supplementary Figure 34. ^{1}H NMR spectra of the compound **B1**.

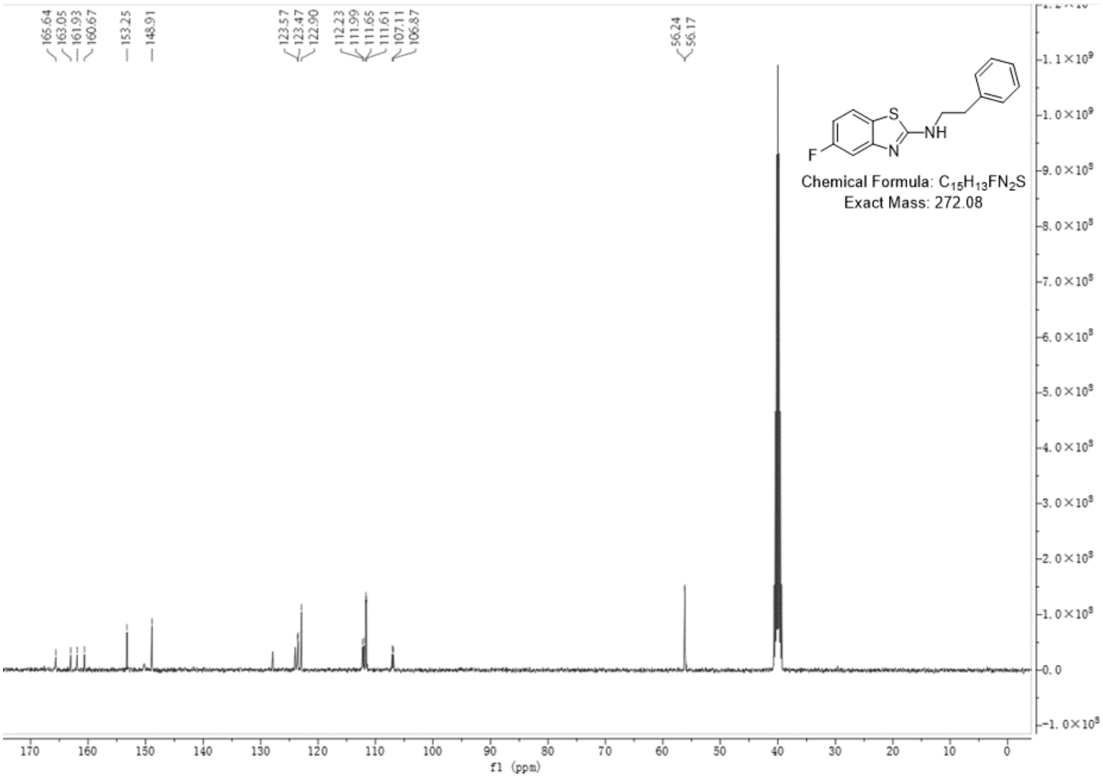

Supplementary Figure 35. ^{13}C NMR spectra of the compound **B1**.

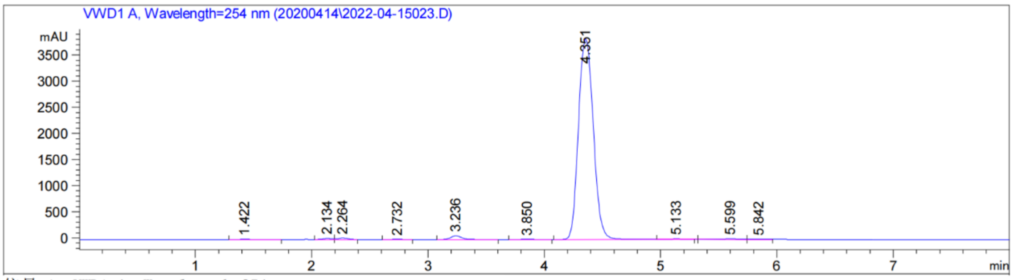

信号 1: VWD1 A, Wavelength=254 nm

峰 #	保留时间 [min]	类型	峰宽 [min]	峰面积 [mAU*s]	峰高 [mAU]	峰面积 %
1	1.422	BV R	0.1161	20.00203	2.71164	0.0571
2	2.134	BV	0.0837	103.95483	19.04805	0.2970
3	2.264	VB	0.0879	153.29745	27.15350	0.4380
4	2.732	BB	0.0872	21.14403	3.78597	0.0604
5	3.236	VB	0.0987	461.60654	72.10818	1.3188
6	3.850	BB	0.1112	26.68293	3.65166	0.0762
7	4.351	BV R	0.1414	3.40221e4	3837.96240	97.1973
8	5.133	VB E	0.1204	69.71523	8.99242	0.1992
9	5.599	BV	0.1348	97.73940	10.88426	0.2792
10	5.842	VV	0.1398	26.88429	2.90825	0.0768
总量 :				3.50031e4	3989.20633	

Supplementary Figure 36. HPLC of the compound **B1.**

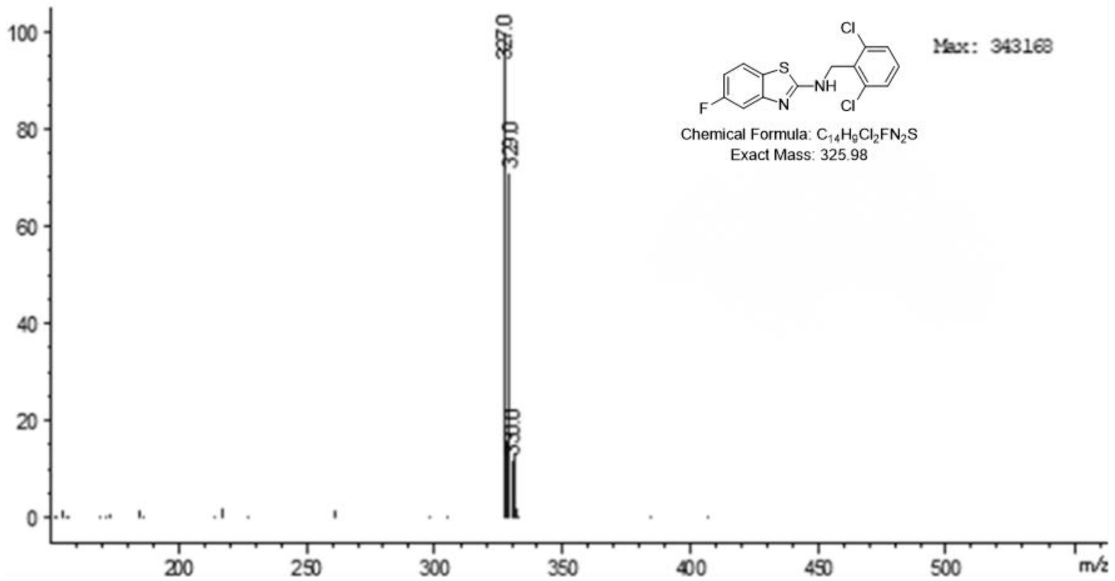

Supplementary Figure 37. Mass spectra of the compound **B2**.

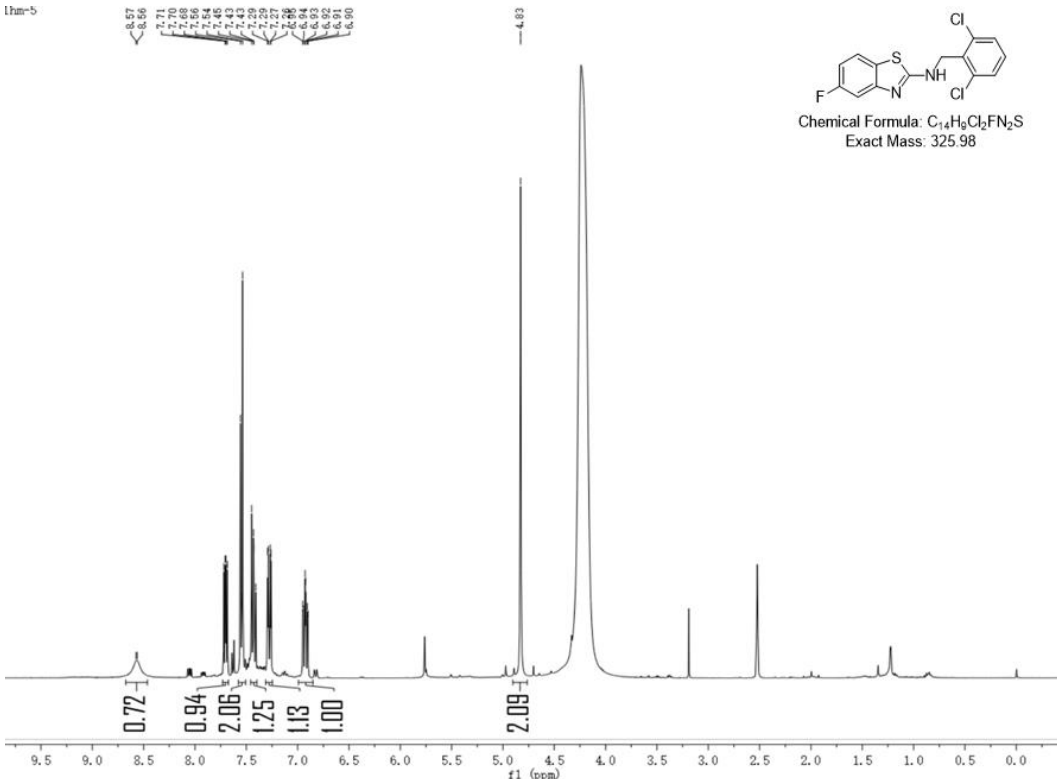

Supplementary Figure 38. ^{1}H NMR spectra of the compound **B2**.

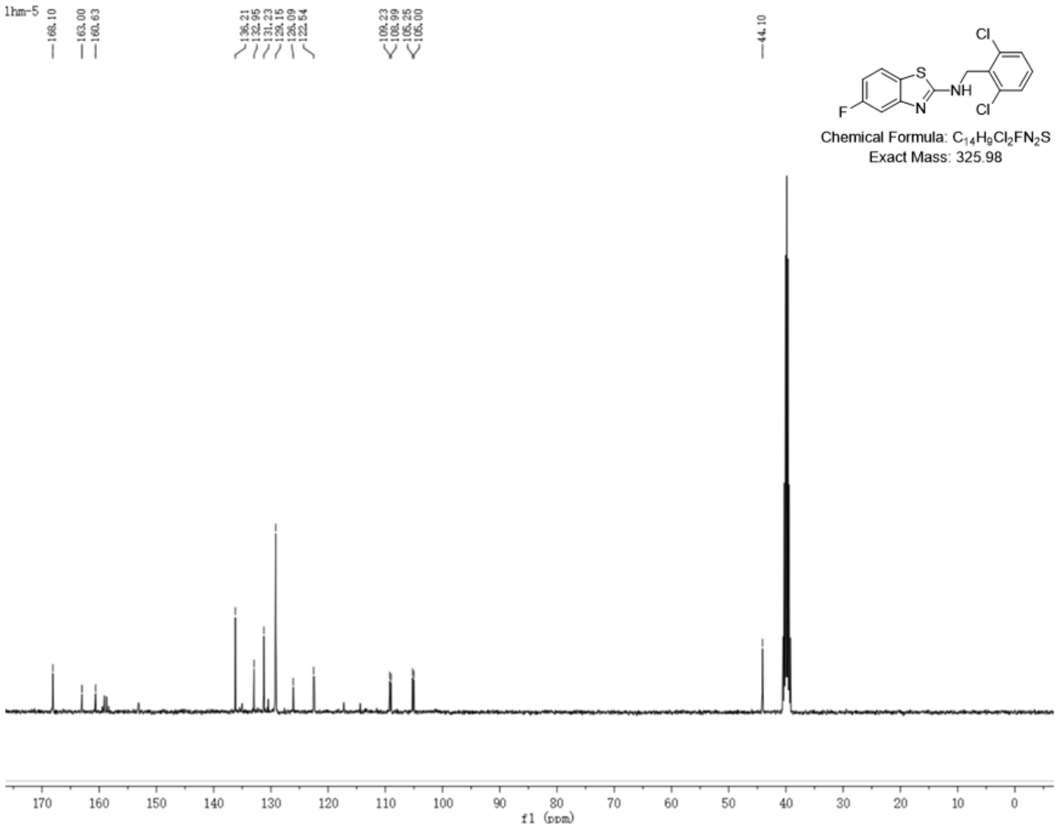

Supplementary Figure 39. ^{13}C NMR spectra of the compound **B2**.

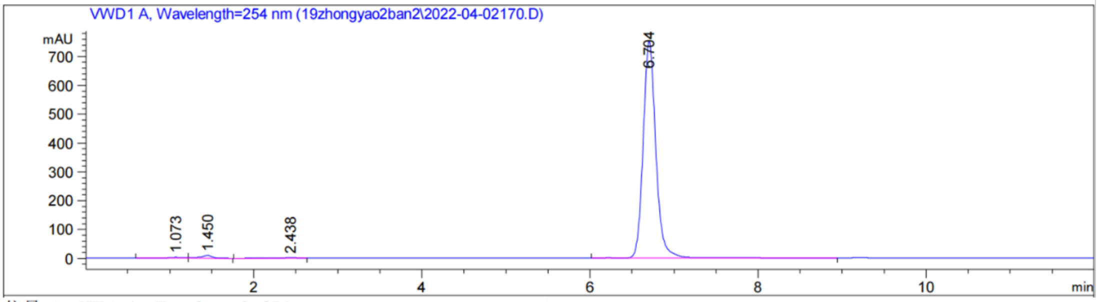

信号 1: VWD1 A, Wavelength=254 nm

峰 #	保留时间 [min]	类型	峰宽 [min]	峰面积 [mAU*s]	峰高 [mAU]	峰面积 %
1	1.073	BV	0.1866	40.33115	3.04347	0.4984
2	1.450	VB	0.1469	103.16669	9.95220	1.2748
3	2.438	BB	0.1825	33.88661	2.55858	0.4187
4	6.704	VB R	0.1609	7915.22754	751.15479	97.8081
总量 :				8092.61200	766.70903	

Supplementary Figure 40. HPLC of the compound **B2.**

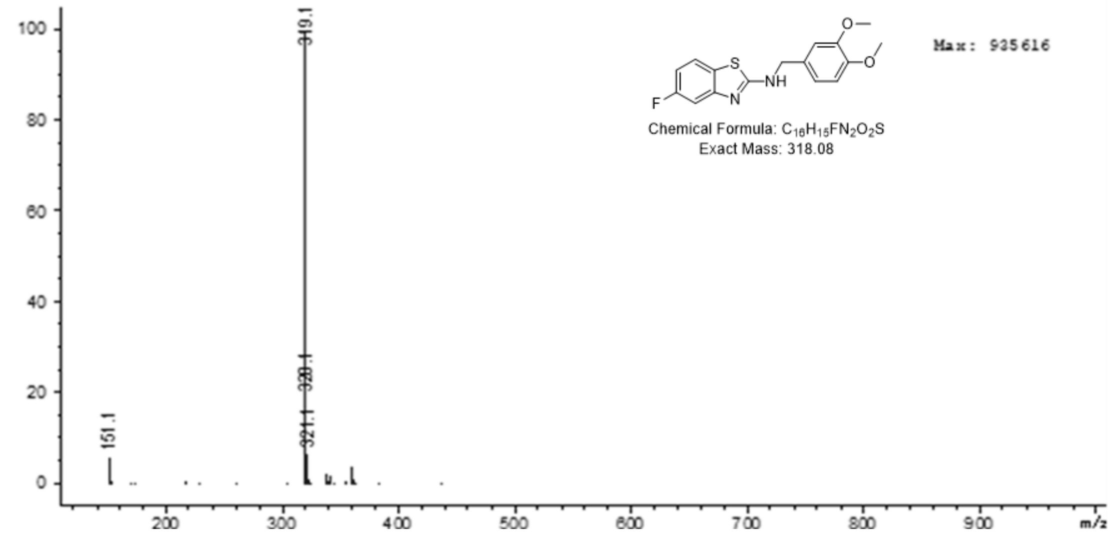

Supplementary Figure 41. Mass spectra of the compound **B3**.

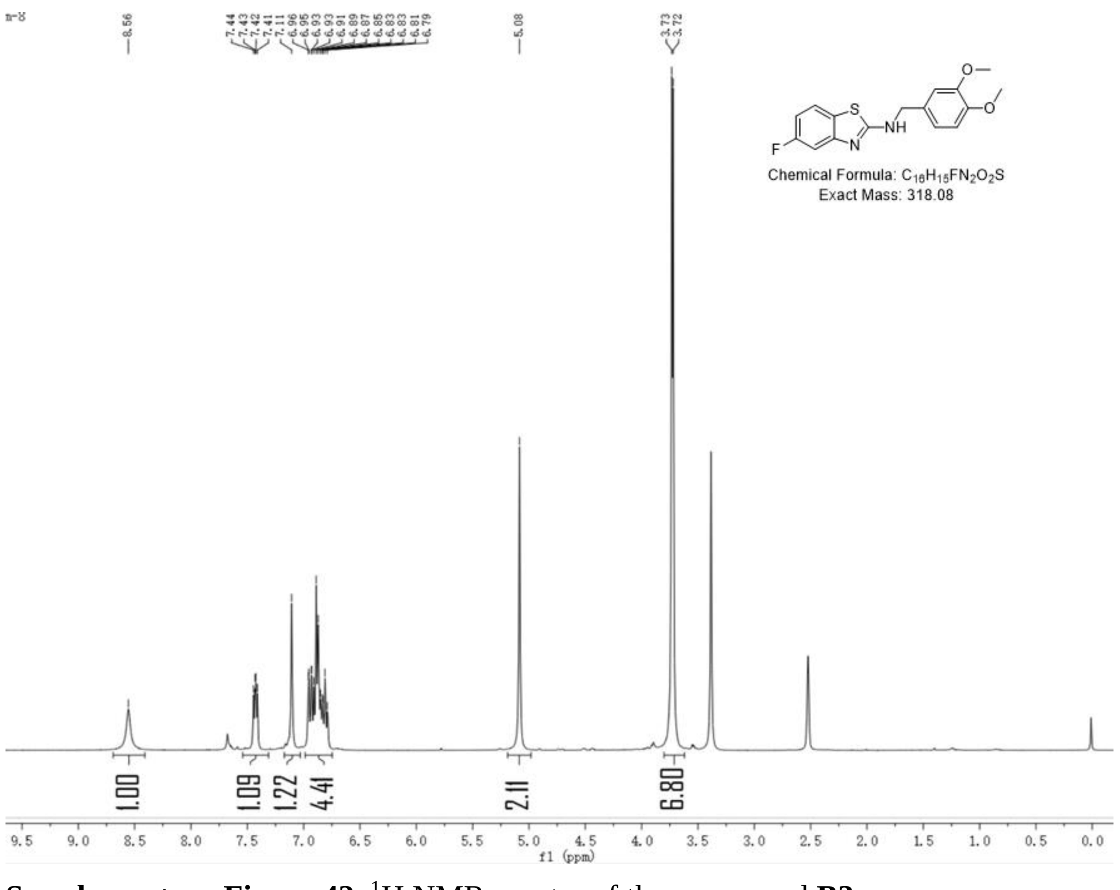

Supplementary Figure 42. ^{1}H NMR spectra of the compound **B3**.

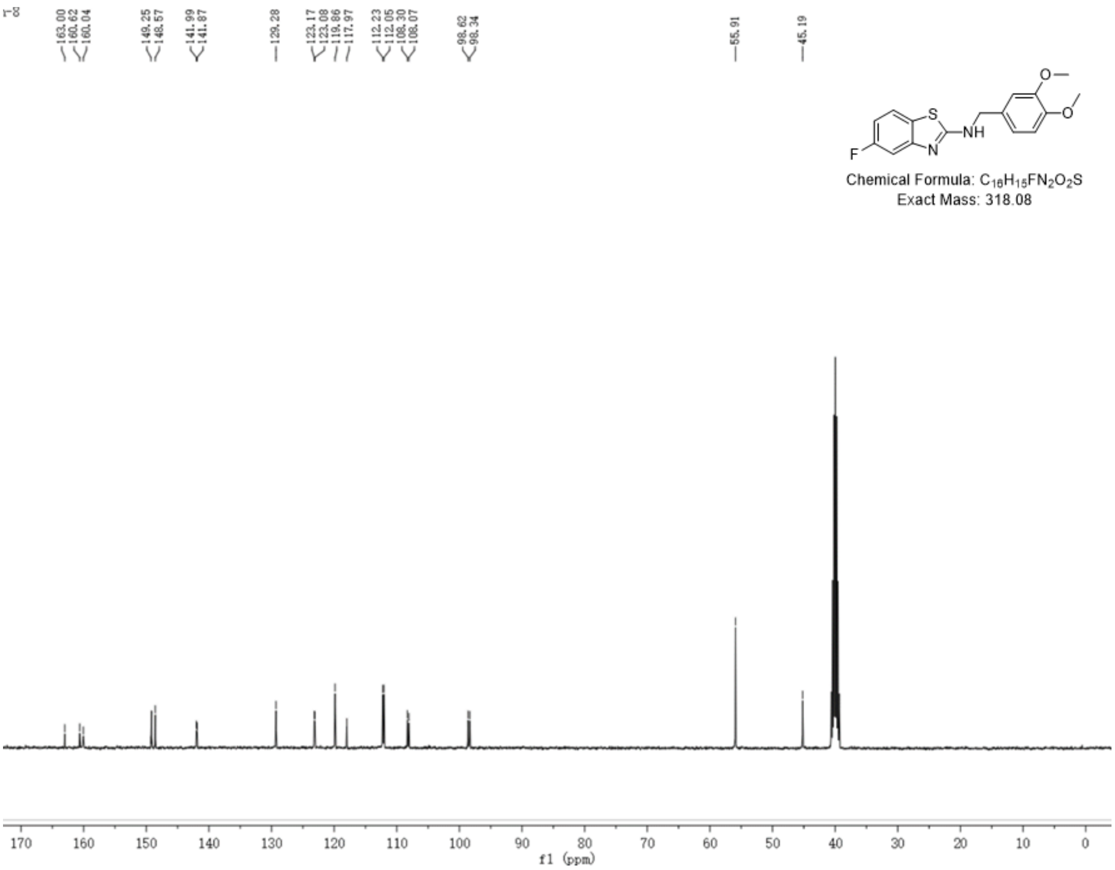

Supplementary Figure 43. ^{13}C NMR spectra of the compound **B3**.

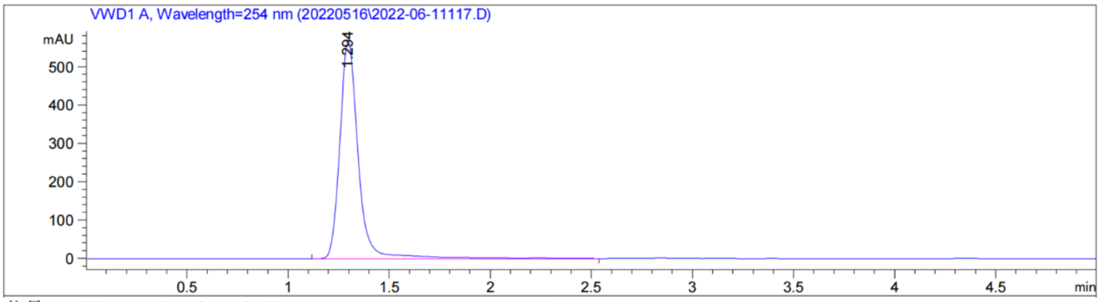

信号 1: VWD1 A, Wavelength=254 nm

峰 #	保留时间 [min]	类型	峰宽 [min]	峰面积 [mAU*s]	峰高 [mAU]	峰面积 %
1	1.294	BV R	0.0986	3627.86719	567.36652	100.0000
总量 :				3627.86719	567.36652	

Supplementary Figure 44. HPLC of the compound **B3.**

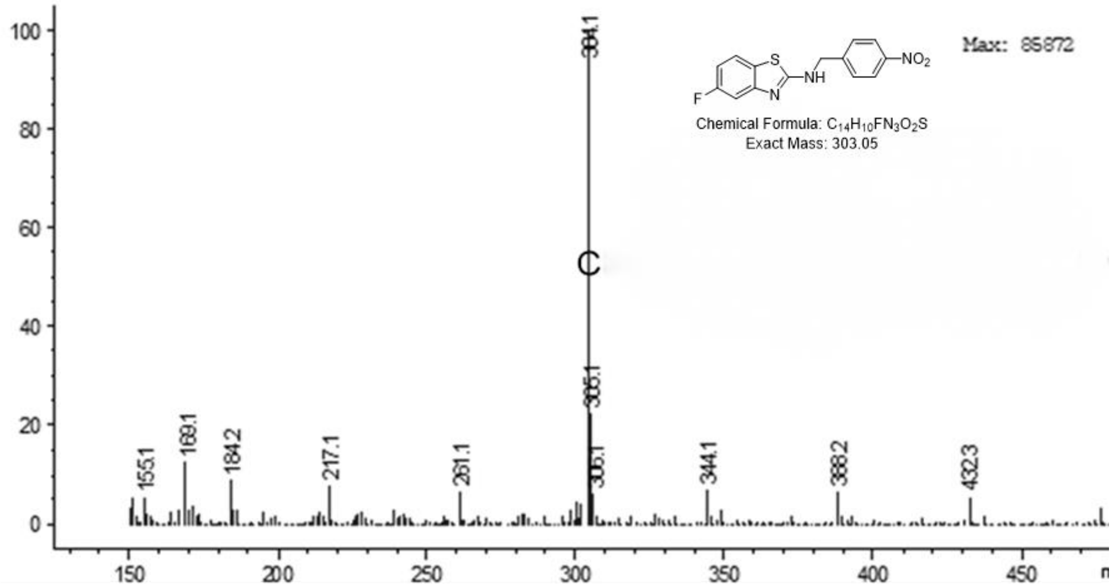

Supplementary Figure 45. Mass spectra of the compound **B4**.

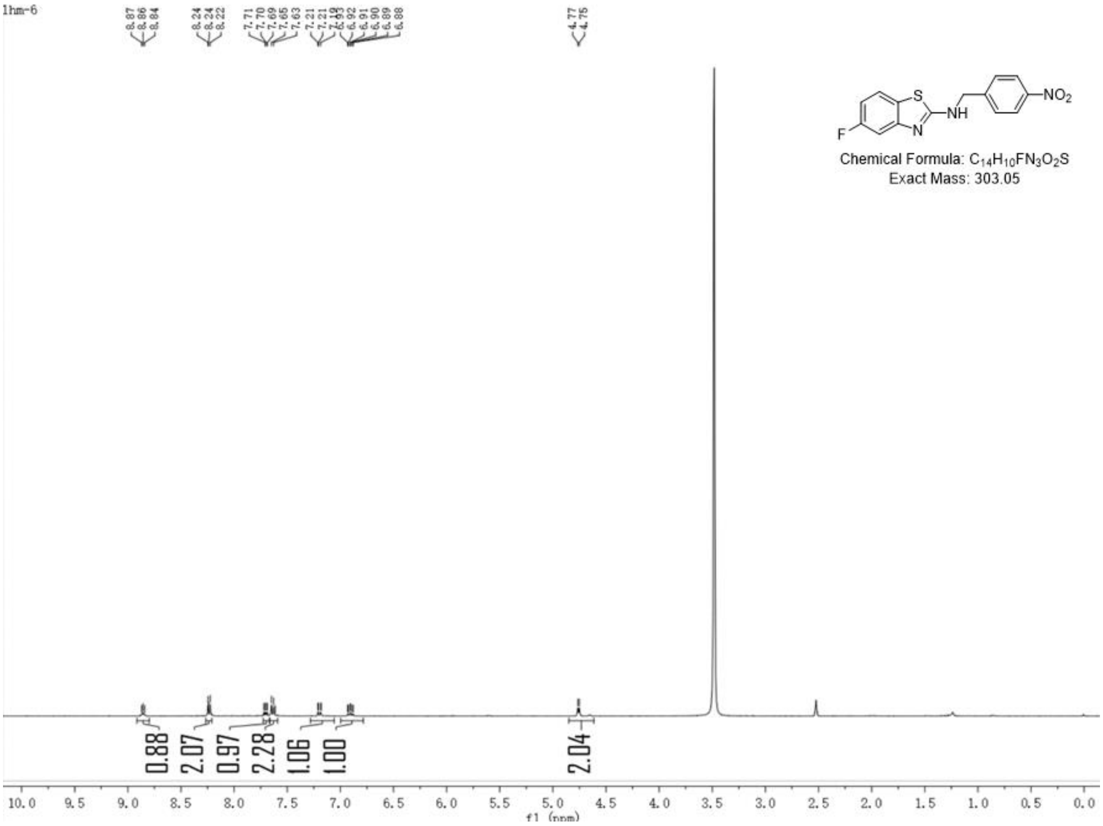

Supplementary Figure 46. ^{1}H NMR spectra of the compound **B4**.

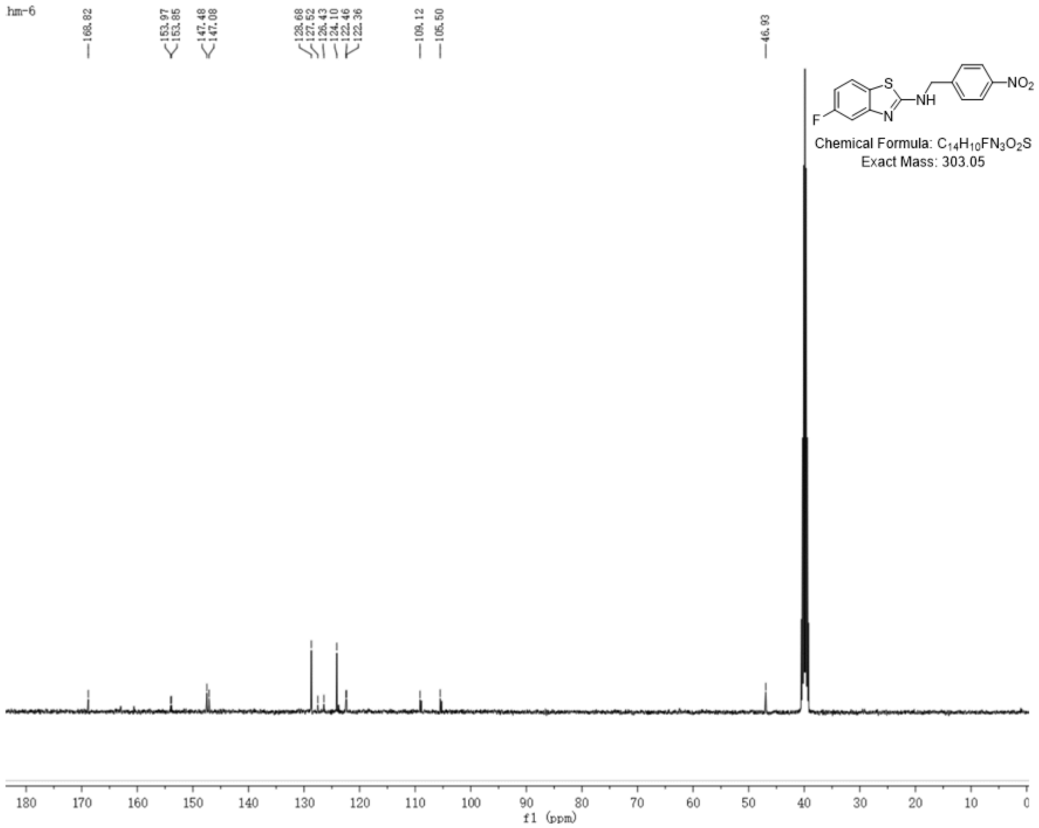

Supplementary Figure 47. ^{13}C NMR spectra of the compound **B4**.

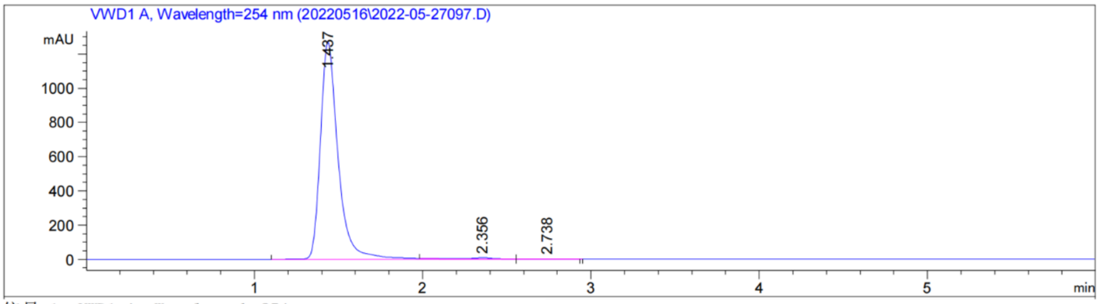

信号 1: VWD1 A, Wavelength=254 nm

峰 #	保留时间 [min]	类型	峰宽 [min]	峰面积 [mAU*s]	峰高 [mAU]	峰面积 %
1	1.437	BV R	0.1114	9306.30566	1272.00769	98.8510
2	2.356	VV E	0.1471	86.32014	8.18251	0.9169
3	2.738	VB E	0.1640	21.85567	1.87508	0.2321
总量 :				9414.48148	1282.06529	

Supplementary Figure 48. HPLC of the compound **B4.**

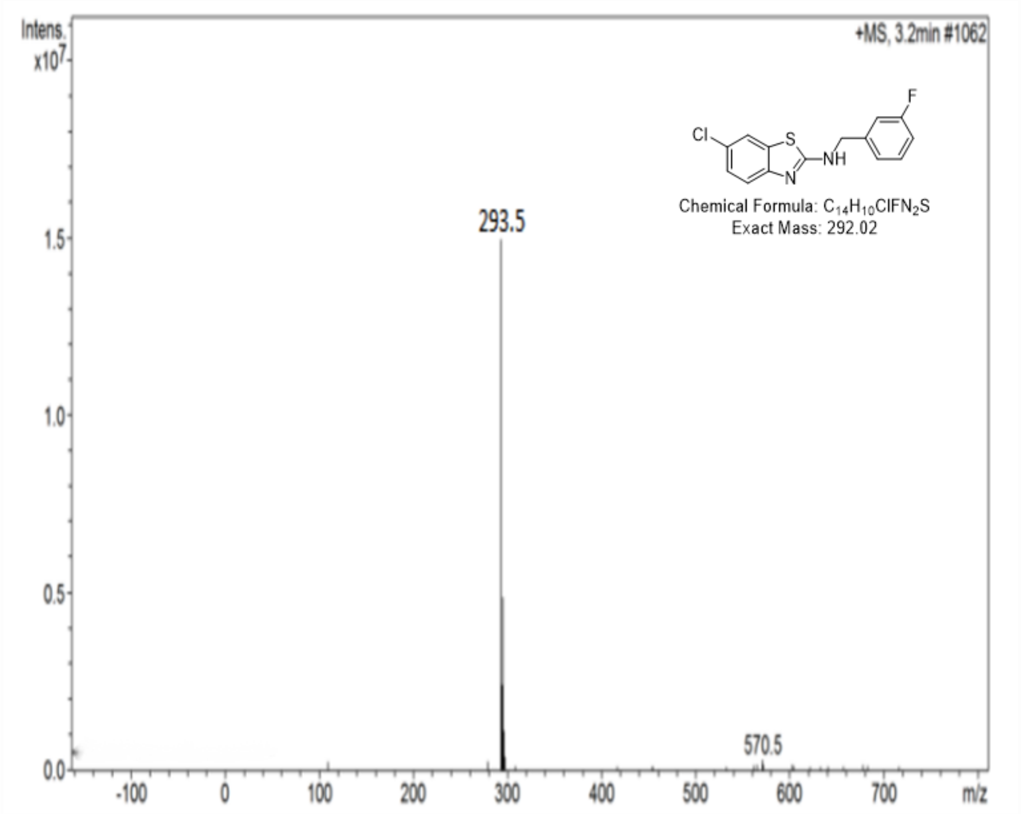

Supplementary Figure 49. Mass spectra of the compound **B5**.

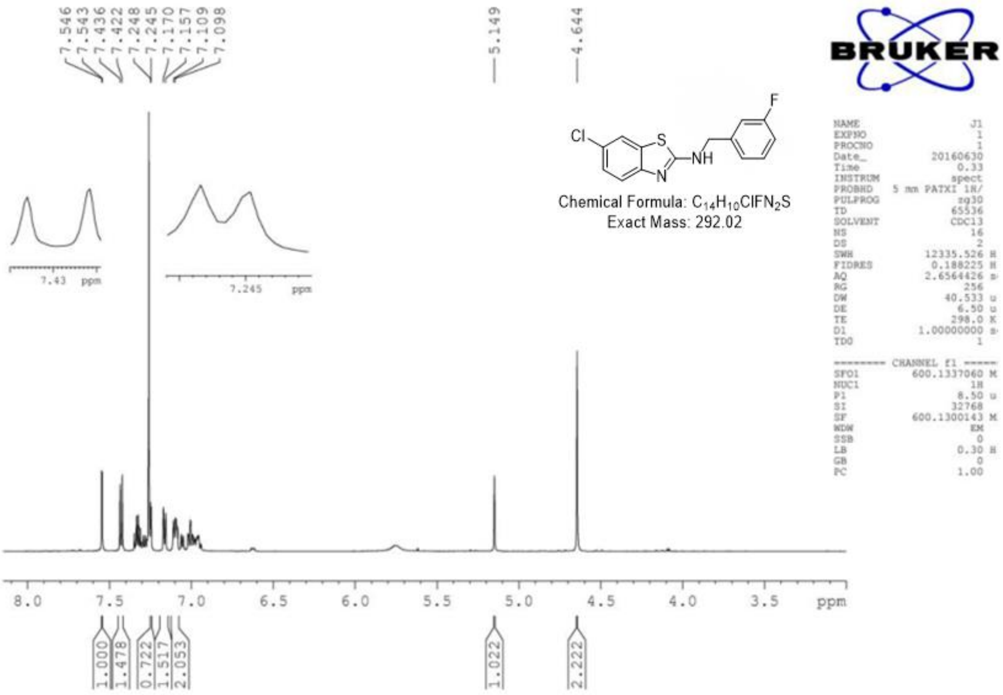

Supplementary Figure 50. ^{1}H NMR spectra of the compound **B5**.

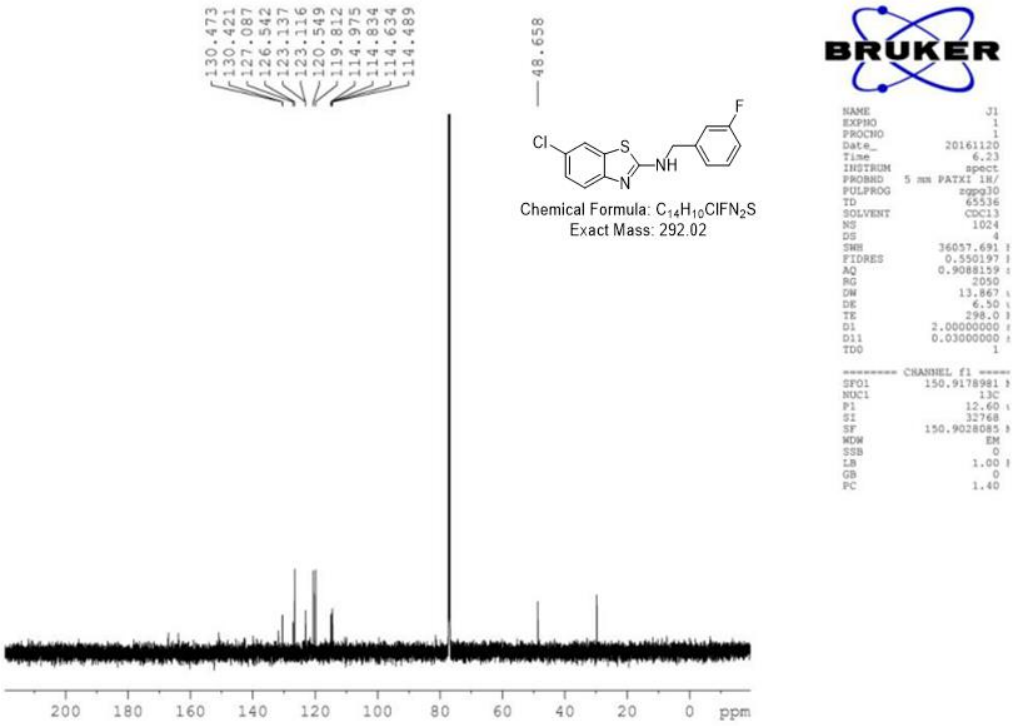

Supplementary Figure 51. ^{13}C NMR spectra of the compound **B5**.

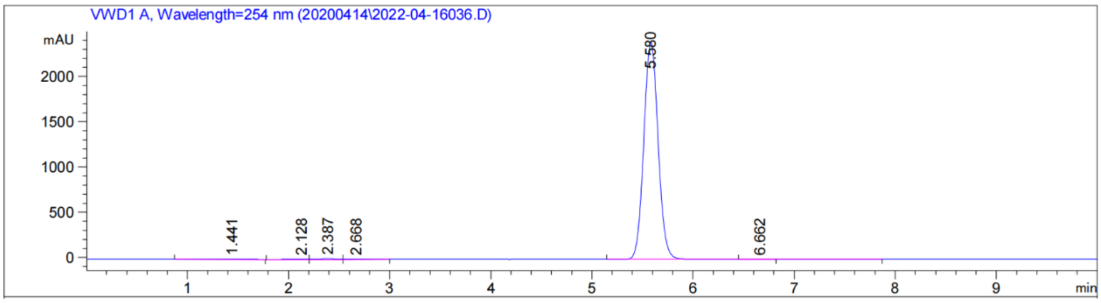

信号 1: VWD1 A, Wavelength=254 nm

峰 #	保留时间 [min]	类型	峰宽 [min]	峰面积 [mAU*s]	峰高 [mAU]	峰面积 %
1	1.441	VB R	0.2301	90.49728	5.49748	0.3781
2	2.128	BV	0.2024	39.36668	2.72742	0.1645
3	2.387	VV	0.1897	84.44499	6.85501	0.3528
4	2.668	VB	0.1599	48.12680	4.68145	0.2011
5	5.580	BV R	0.1530	2.36479e4	2398.47437	98.8083
6	6.662	VV E	0.1564	22.76661	2.32222	0.0951

总量 : 2.39331e4 2420.55795

Supplementary Figure 52. HPLC of the compound **B5.**

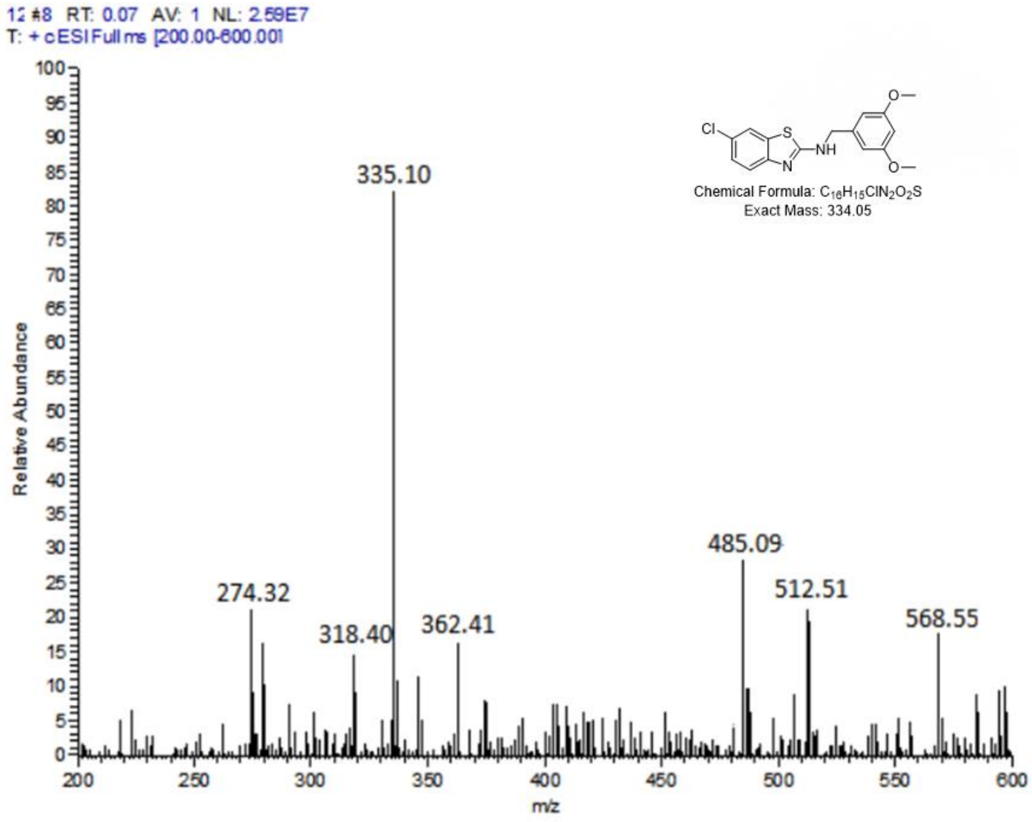

Supplementary Figure 53. Mass spectra of the compound **B6**.

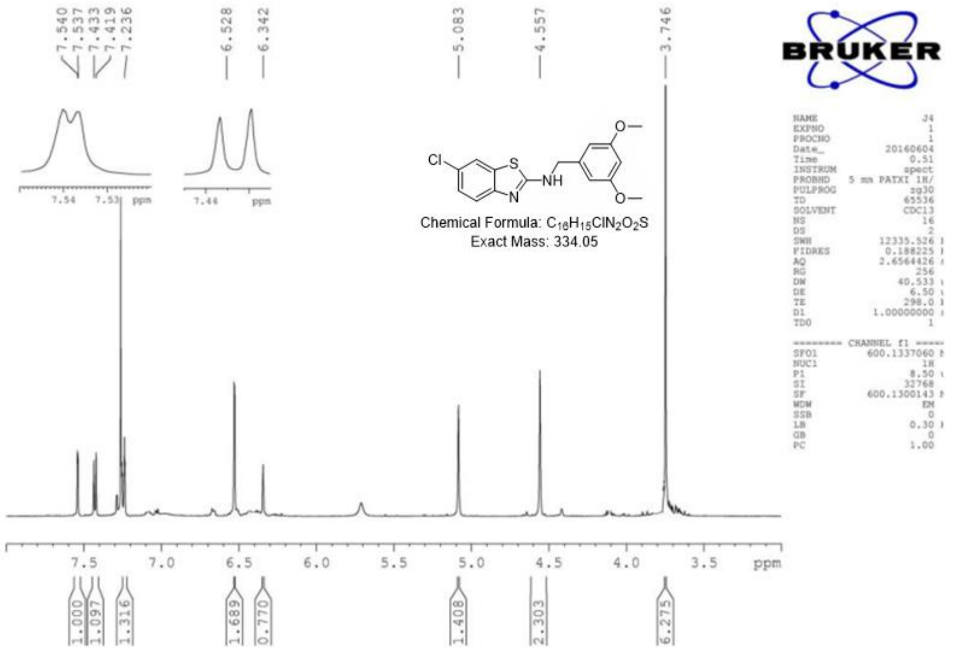

Supplementary Figure 54. ^{1}H NMR spectra of the compound **B6**.

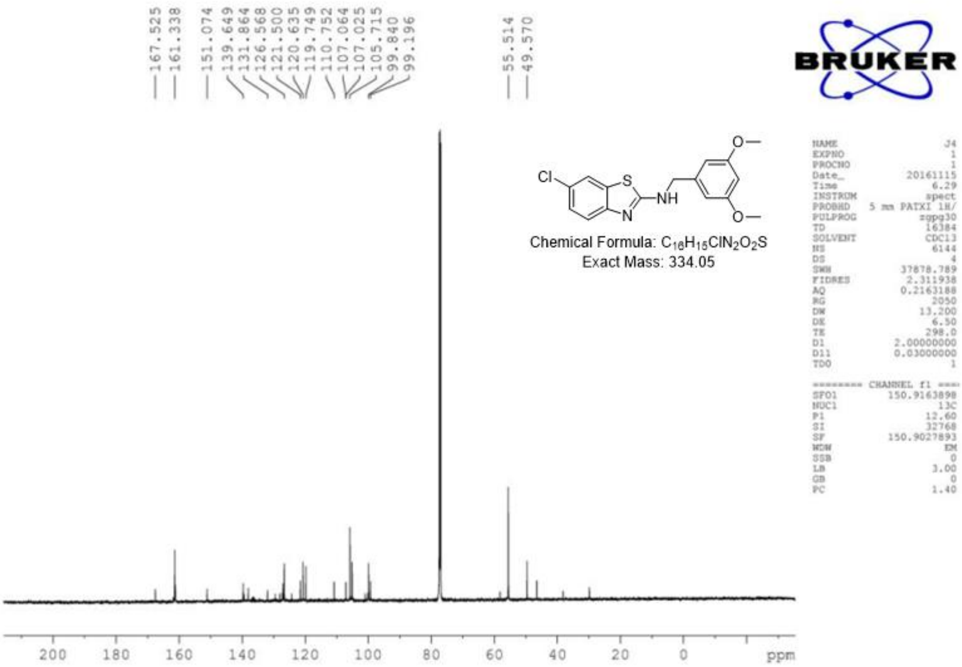

Supplementary Figure 55. ^{13}C NMR spectra of the compound **B6**.

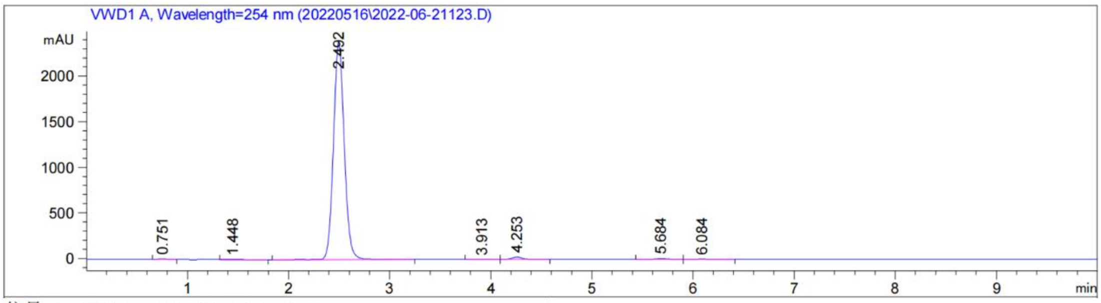

信号 1: VWD1 A, Wavelength=254 nm

峰 #	保留时间 [min]	类型	峰宽 [min]	峰面积 [mAU*s]	峰高 [mAU]	峰面积 %
1	0.751	VB	0.0903	44.39107	7.36489	0.2438
2	1.448	VB	0.1823	24.92228	2.07237	0.1369
3	2.492	VV R	0.1167	1.77363e4	2385.54199	97.3922
4	3.913	VV	0.1403	32.54037	3.38163	0.1787
5	4.253	VB	0.1233	214.48949	26.83306	1.1778
6	5.684	BV	0.1413	105.43229	11.45937	0.5789
7	6.084	VB	0.1523	53.14524	5.42684	0.2918
总量 :				1.82112e4	2442.08015	

Supplementary Figure 56. HPLC of the compound **B6.**

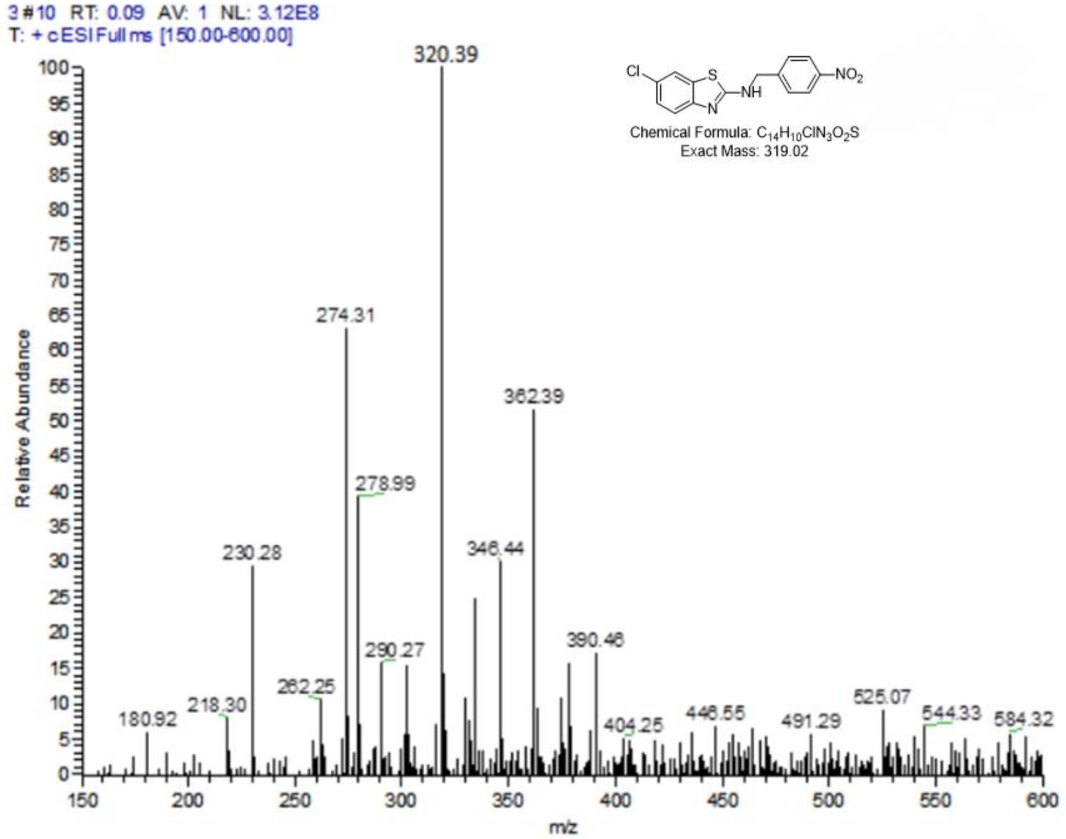

Supplementary Figure 57. Mass spectra of the compound **B7**.

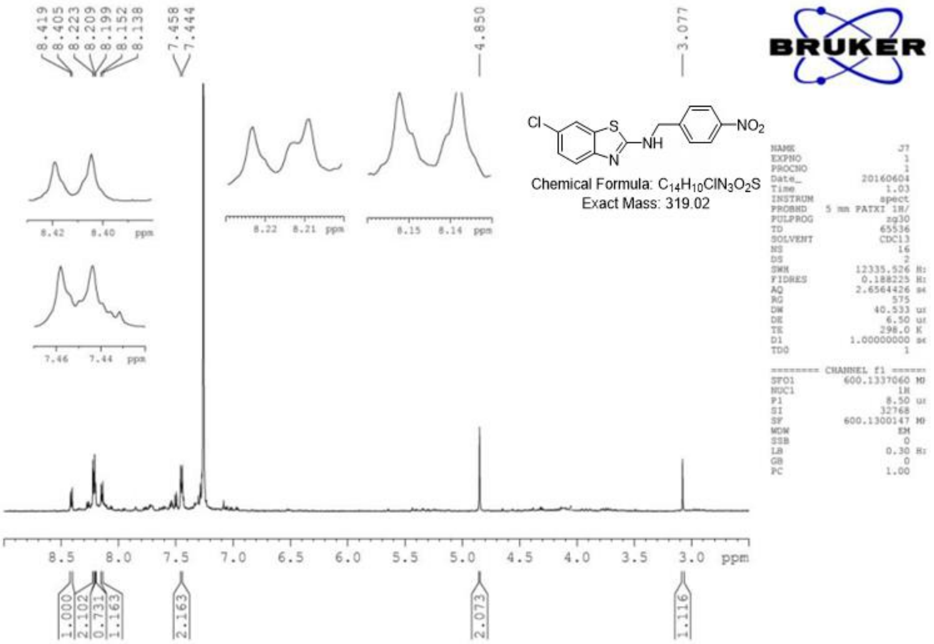

Supplementary Figure 58. ^{1}H NMR spectra of the compound **B7**.

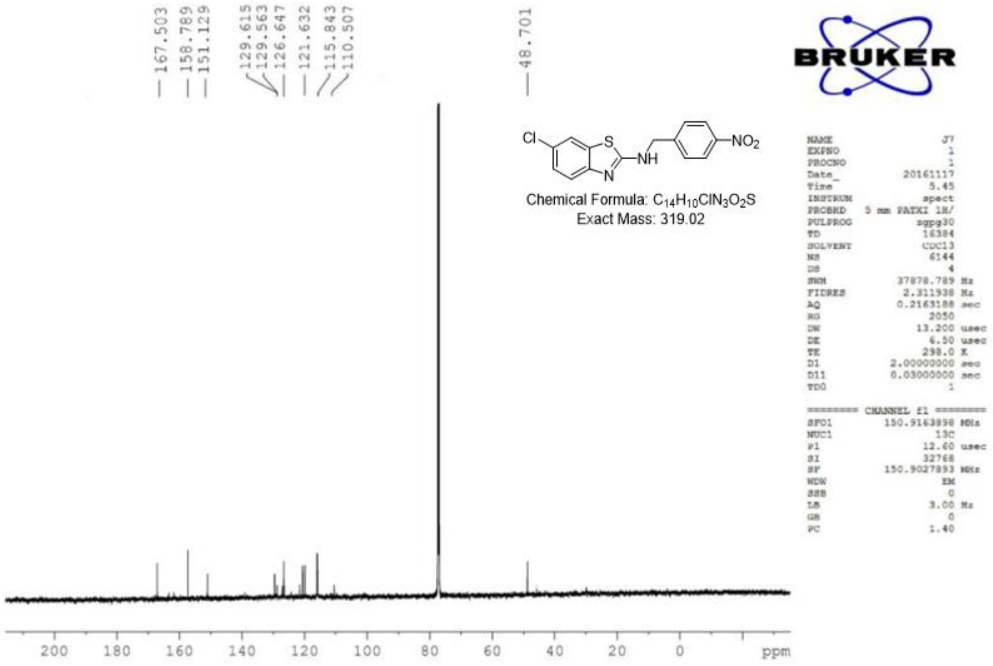

Supplementary Figure 59. ^{13}C NMR spectra of the compound **B7**.

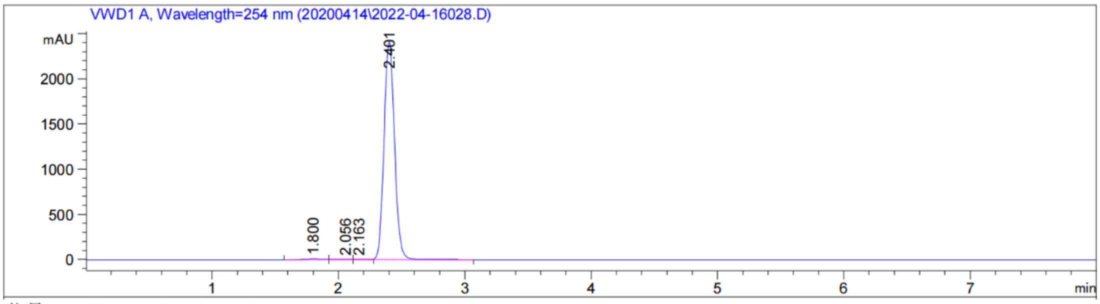

信号 1: VWD1 A, Wavelength=254 nm

峰 #	保留时间 [min]	类型	峰宽 [min]	峰面积 [mAU*s]	峰高 [mAU]	峰面积 %
1	1.800	BB	0.0915	39.38668	6.80413	0.2829
2	2.056	BV E	0.0907	20.57509	3.39330	0.1478
3	2.163	VV E	0.0976	18.12350	2.79850	0.1302
4	2.401	VB R	0.0910	1.38458e4	2412.68262	99.4392

总量 : 1.39239e4 2425.67855

Supplementary Figure 60. HPLC of the compound **B7.**

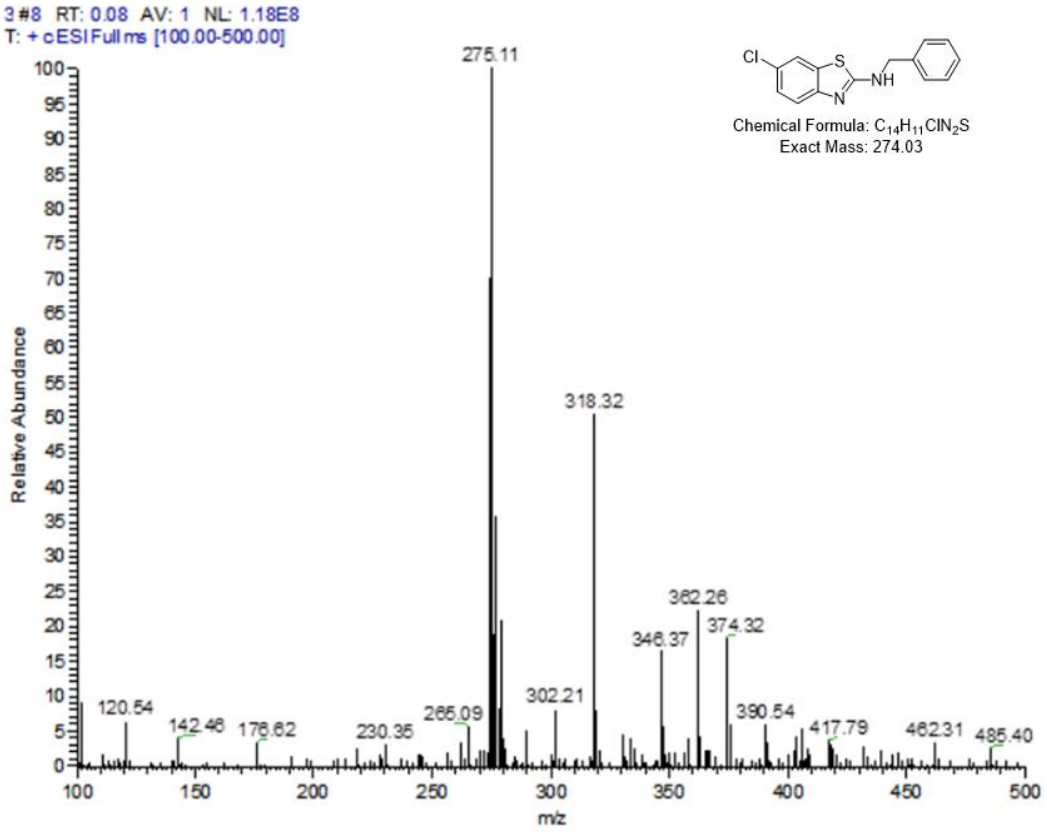

Supplementary Figure 61. Mass spectra of the compound **B8**.

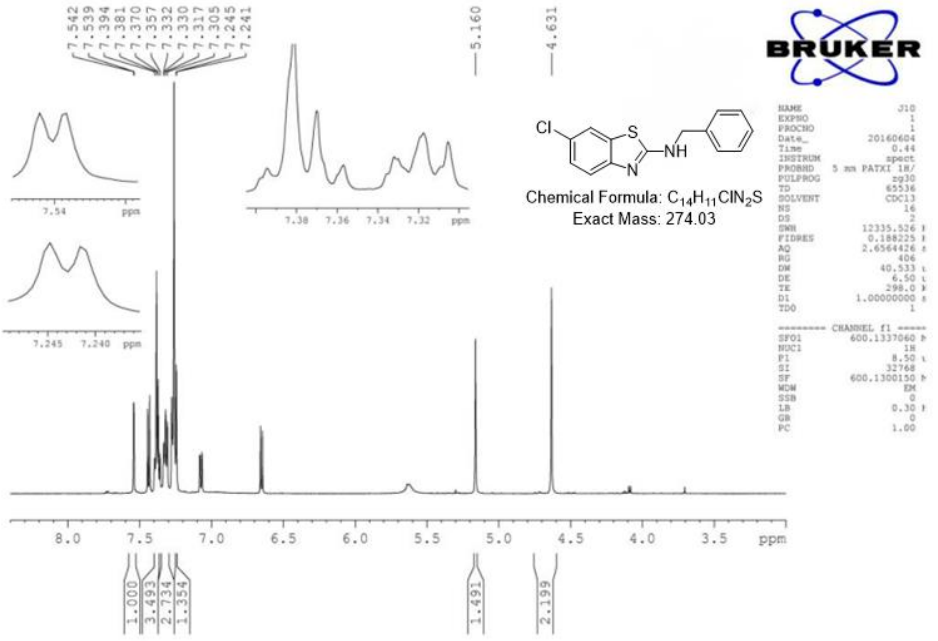

Supplementary Figure 62. ^{1}H NMR spectra of the compound **B8**.

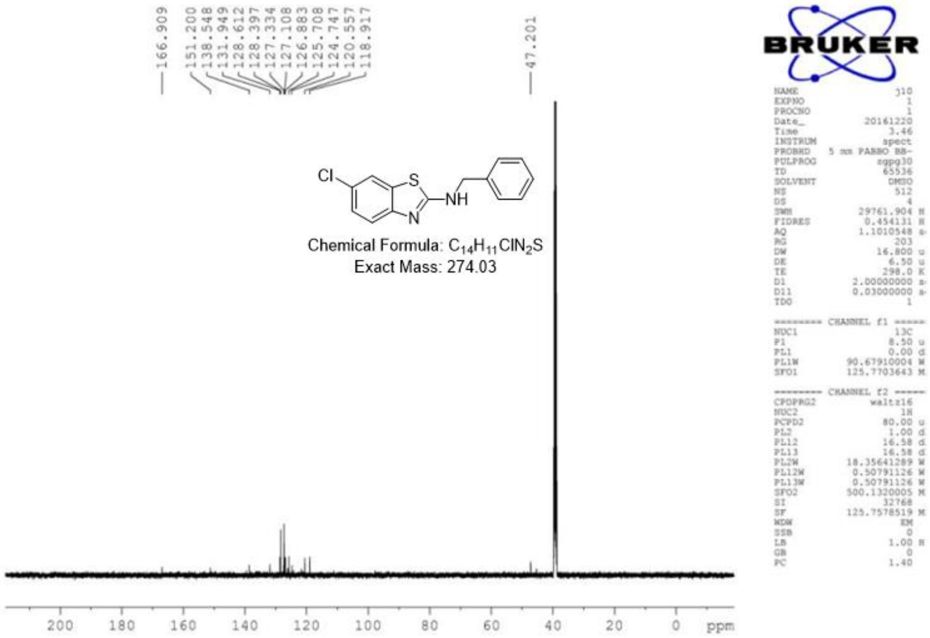

Supplementary Figure 63. ^{13}C NMR spectra of the compound **B8**.

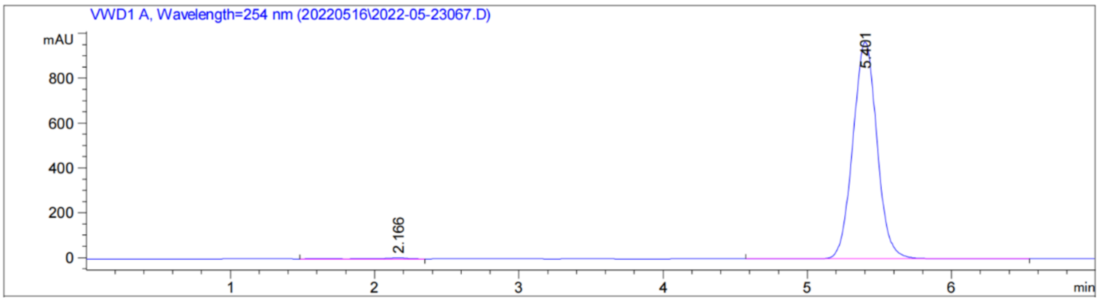

信号 1: VWD1 A, Wavelength=254 nm

峰 #	保留时间 [min]	类型	峰宽 [min]	峰面积 [mAU*s]	峰高 [mAU]	峰面积 %
1	2.166	VB R	0.1596	65.12163	5.68766	0.5755
2	5.401	VB R	0.1817	1.12502e4	967.12628	99.4245
总量 :				1.13153e4	972.81394	

Supplementary Figure 64. HPLC of the compound **B8.**

1.3 Spectrums of the compounds C1-C7

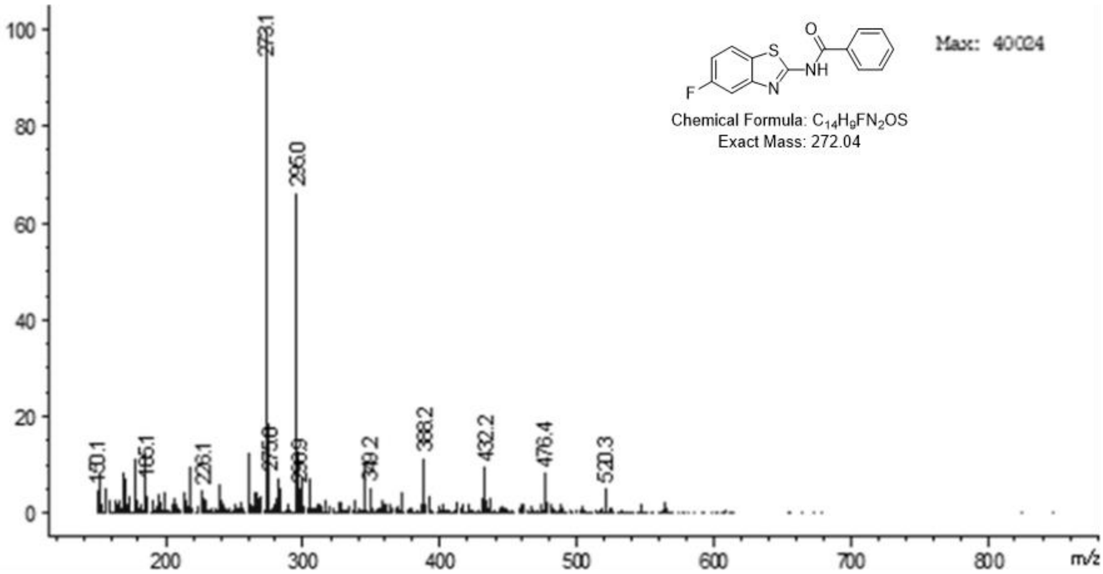

Supplementary Figure 65. Mass spectra of the compound **C1**.

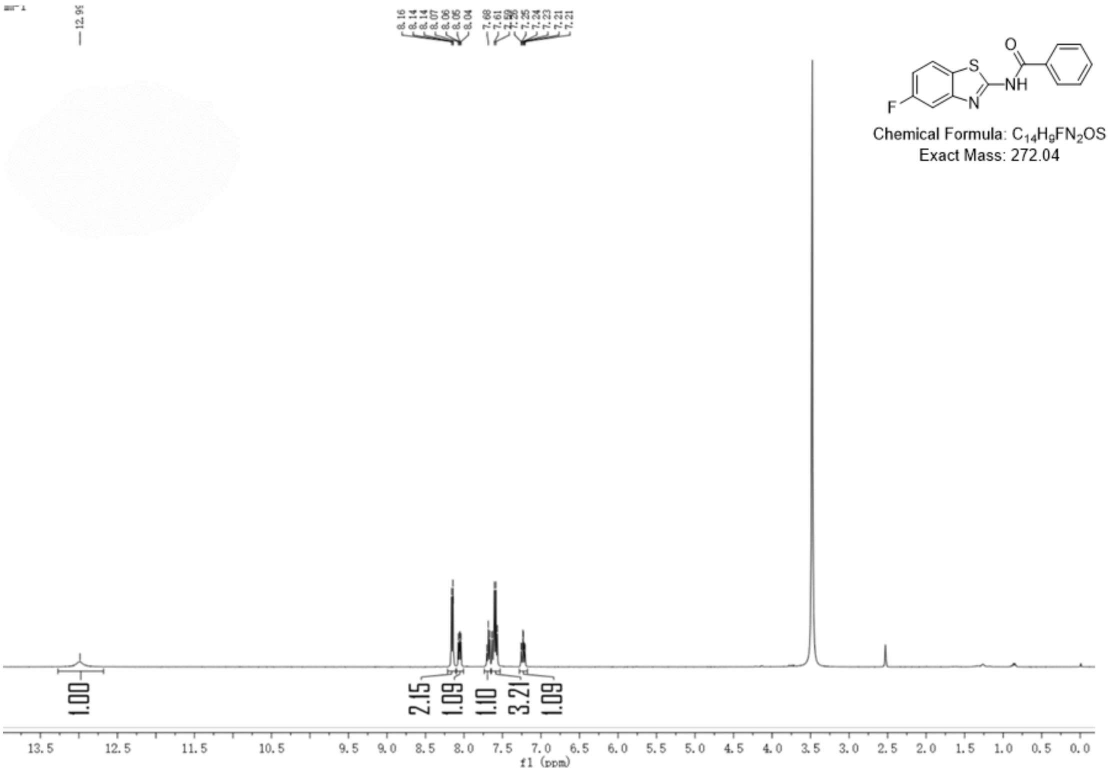

Supplementary Figure 66. ^{1}H NMR spectra of the compound **C1**.

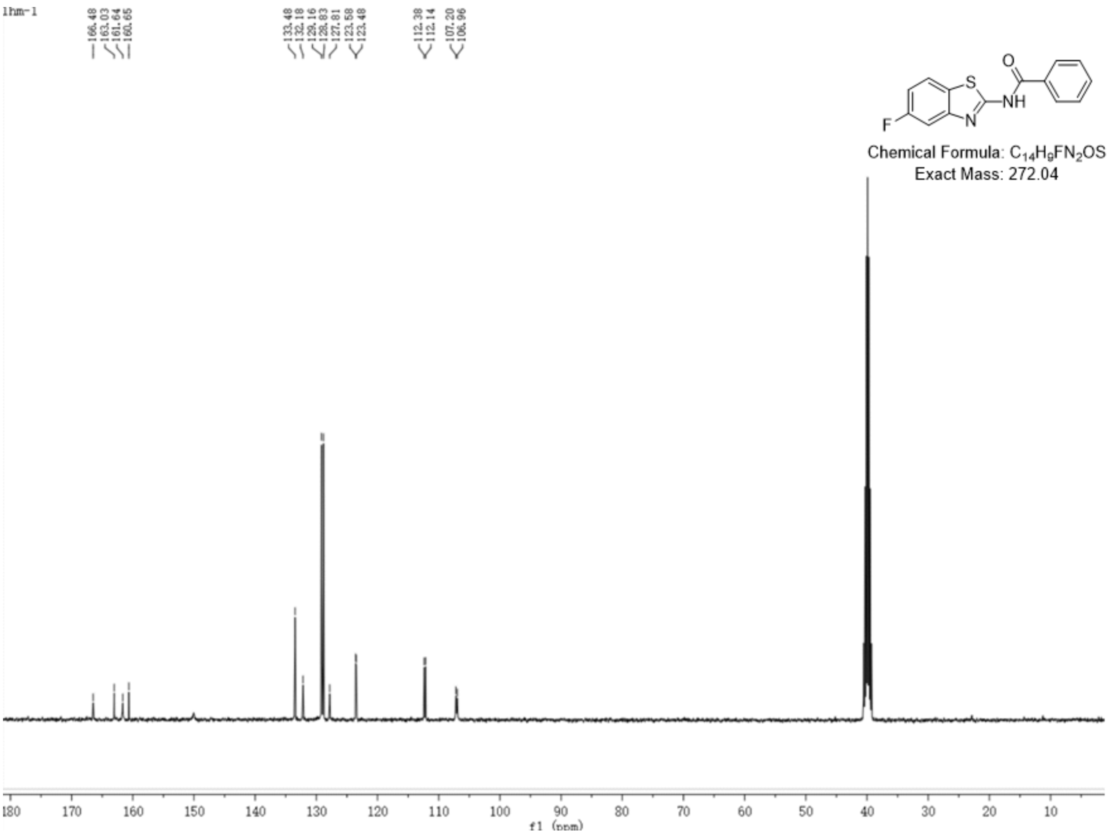

Supplementary Figure 67. ^{13}C NMR spectra of the compound **C1**.

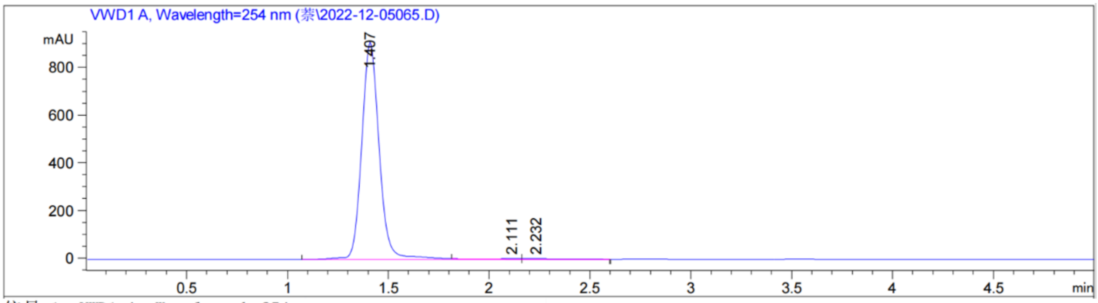

信号 1: VWD1 A, Wavelength=254 nm

峰 #	保留时间 [min]	类型	峰宽 [min]	峰面积 [mAU*s]	峰高 [mAU]	峰面积 %
1	1.407	BV R	0.0928	5528.14844	911.30481	98.8934
2	2.111	VV E	0.1614	26.70189	2.23530	0.4777
3	2.232	VB E	0.1816	35.15814	2.60312	0.6289

Supplementary Figure 68. HPLC of the compound **C1.**

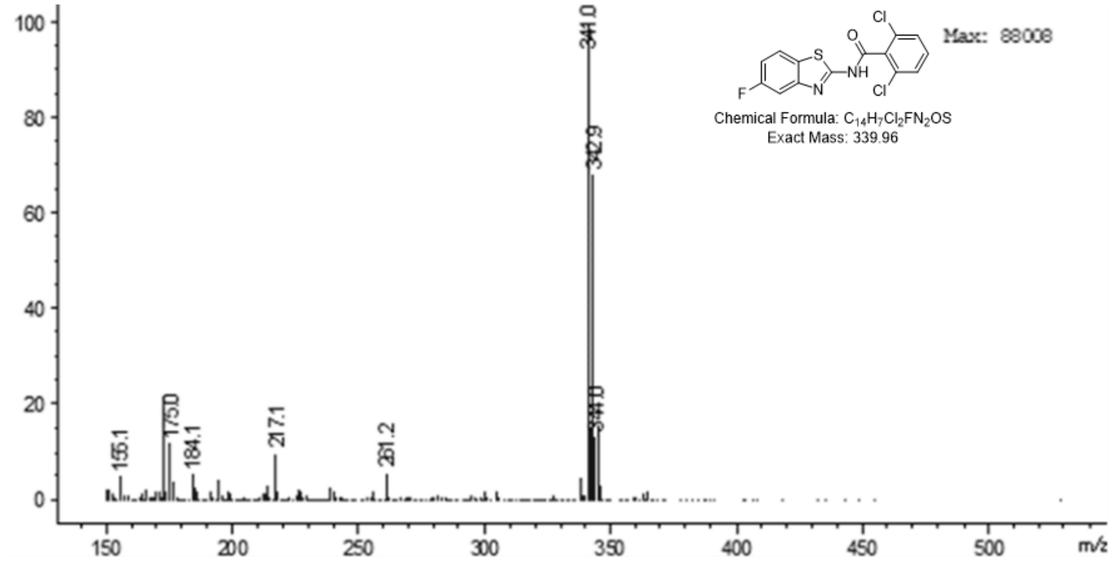

Supplementary Figure 69. Mass spectra of the compound **C2**.

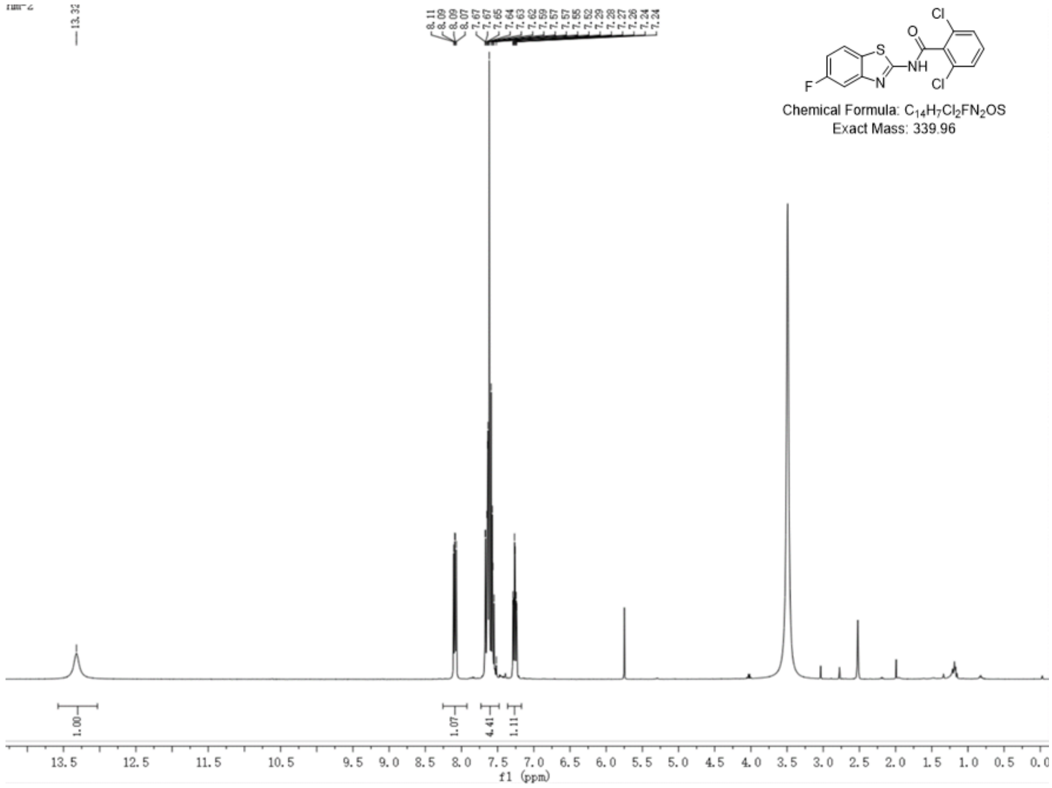

Supplementary Figure 70. ^{1}H NMR spectra of the compound **C2**.

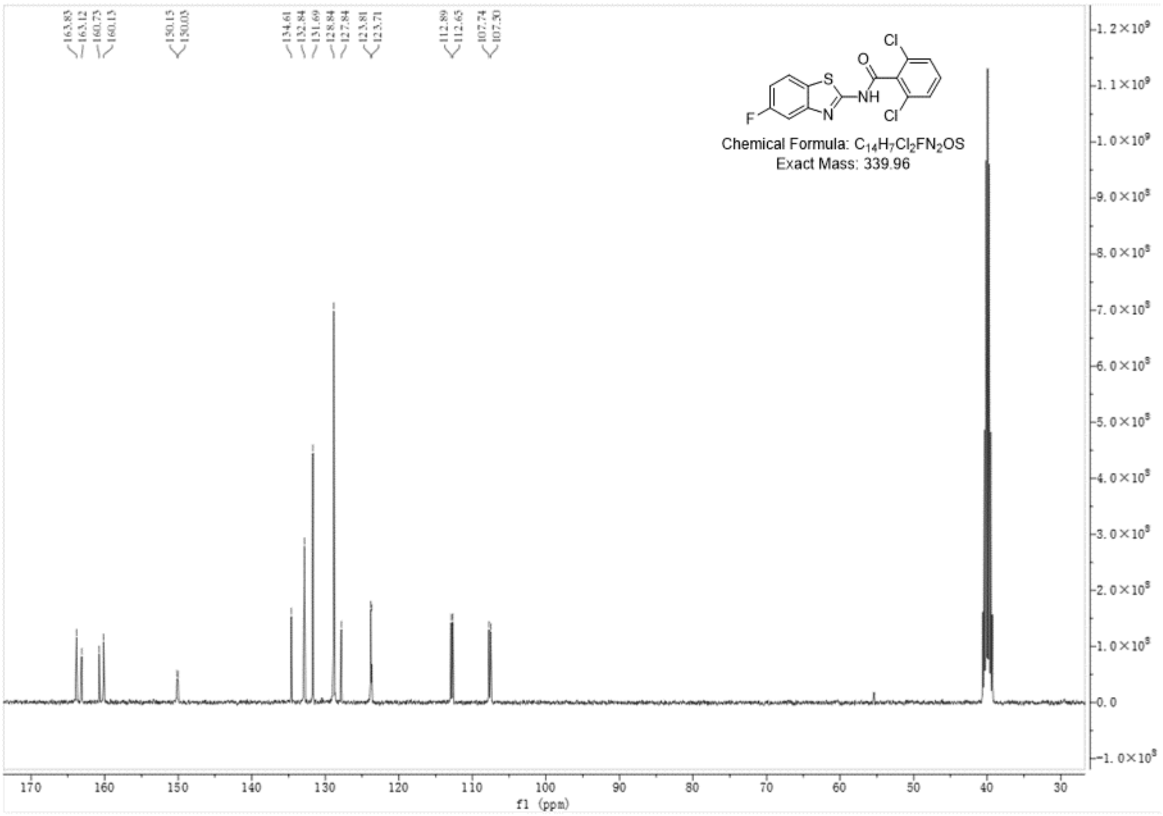

Supplementary Figure 71. ^{13}C NMR spectra of the compound **C2**.

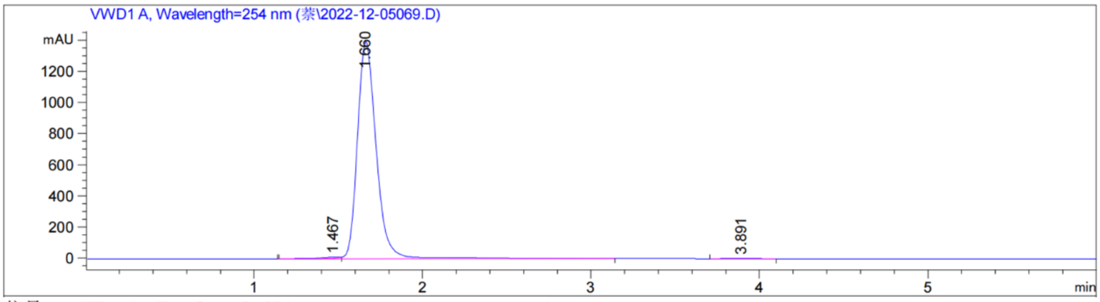

信号 1: VWD1 A, Wavelength=254 nm

峰 #	保留时间 [min]	类型	峰宽 [min]	峰面积 [mAU*s]	峰高 [mAU]	峰面积 %
1	1.467	BV E	0.1328	65.63139	7.16690	0.5953
2	1.660	VV R	0.1215	1.09425e4	1395.61694	99.2495
3	3.891	BB	0.1343	17.11805	1.99118	0.1553

总量 : 1.10252e4 1404.77502

Supplementary Figure 72. HPLC of the compound **C2.**

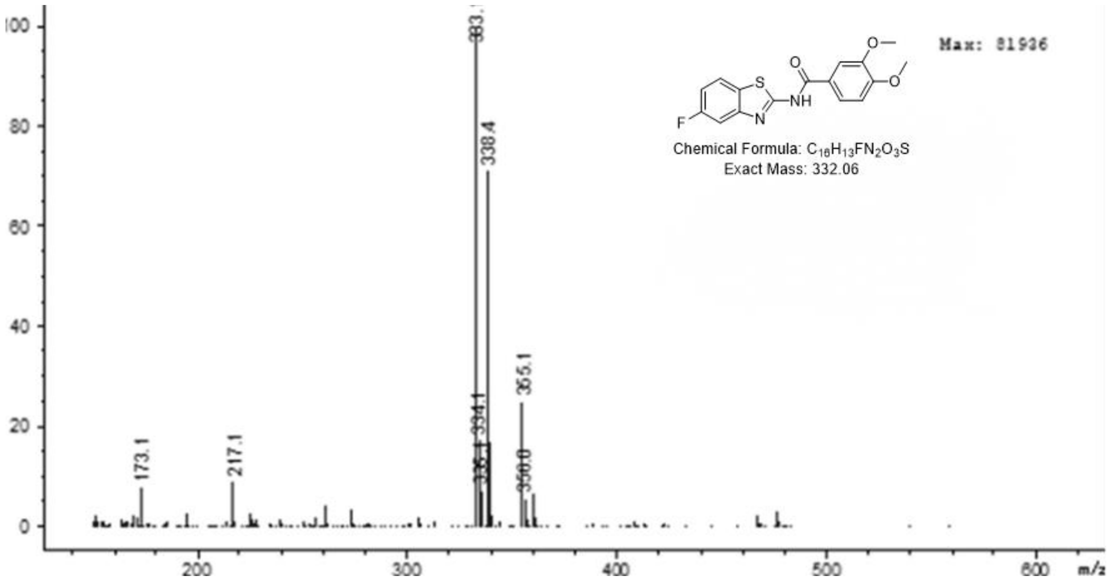

Supplementary Figure 73. Mass spectra of the compound **C3**.

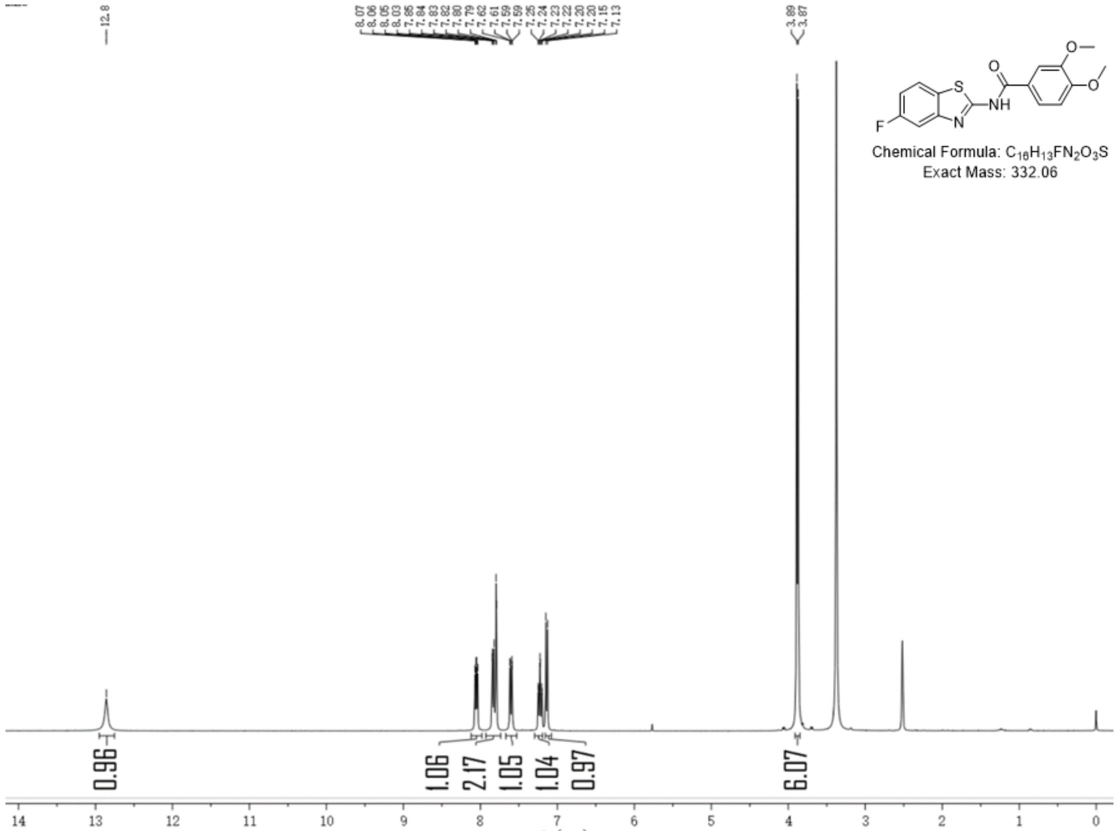

Supplementary Figure 74. ^{1}H NMR spectra of the compound **C3**.

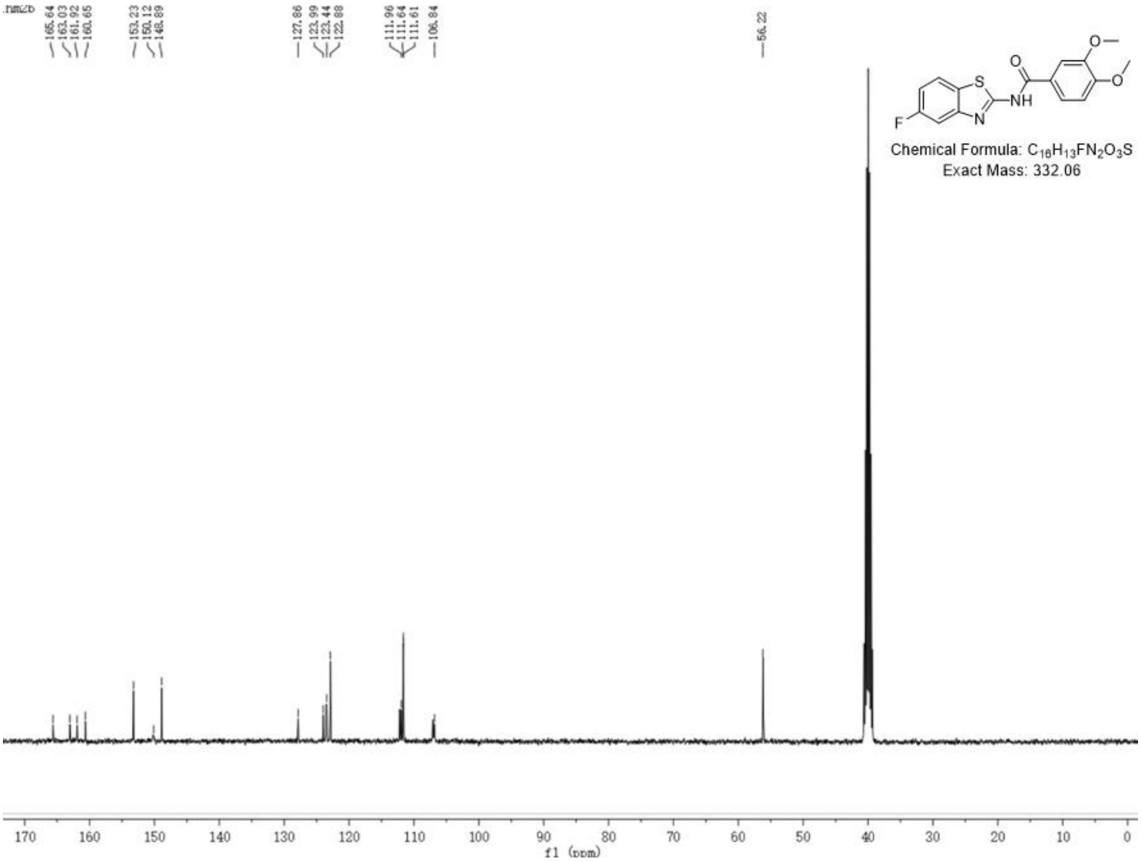

Supplementary Figure 75. ^{13}C NMR spectra of the compound **C3**.

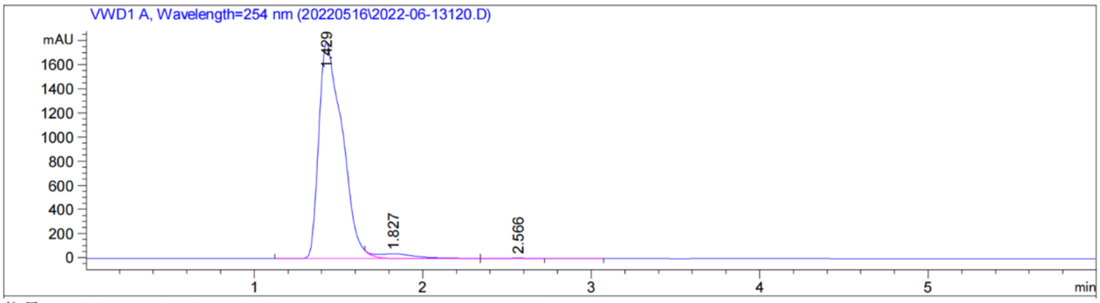

信号 1: VWD1 A, Wavelength=254 nm

峰 #	保留时间 [min]	类型	峰宽 [min]	峰面积 [mAU*s]	峰高 [mAU]	峰面积 %
1	1.429	BV R	0.1366	1.73575e4	1798.01392	96.1992
2	1.827	VV E	0.2336	628.71924	38.28700	3.4845
3	2.566	VV E	0.2002	57.07234	3.95877	0.3163
总量 :				1.80433e4	1840.25969	

Supplementary Figure 76. HPLC of the compound **C3.**

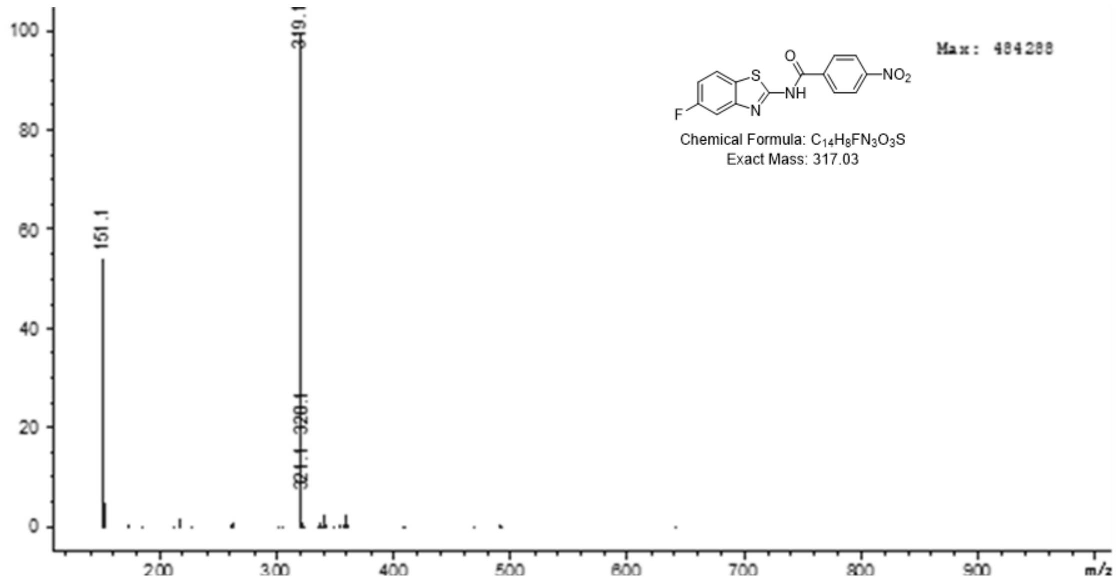

Supplementary Figure 77. Mass spectra of the compound **C4**.

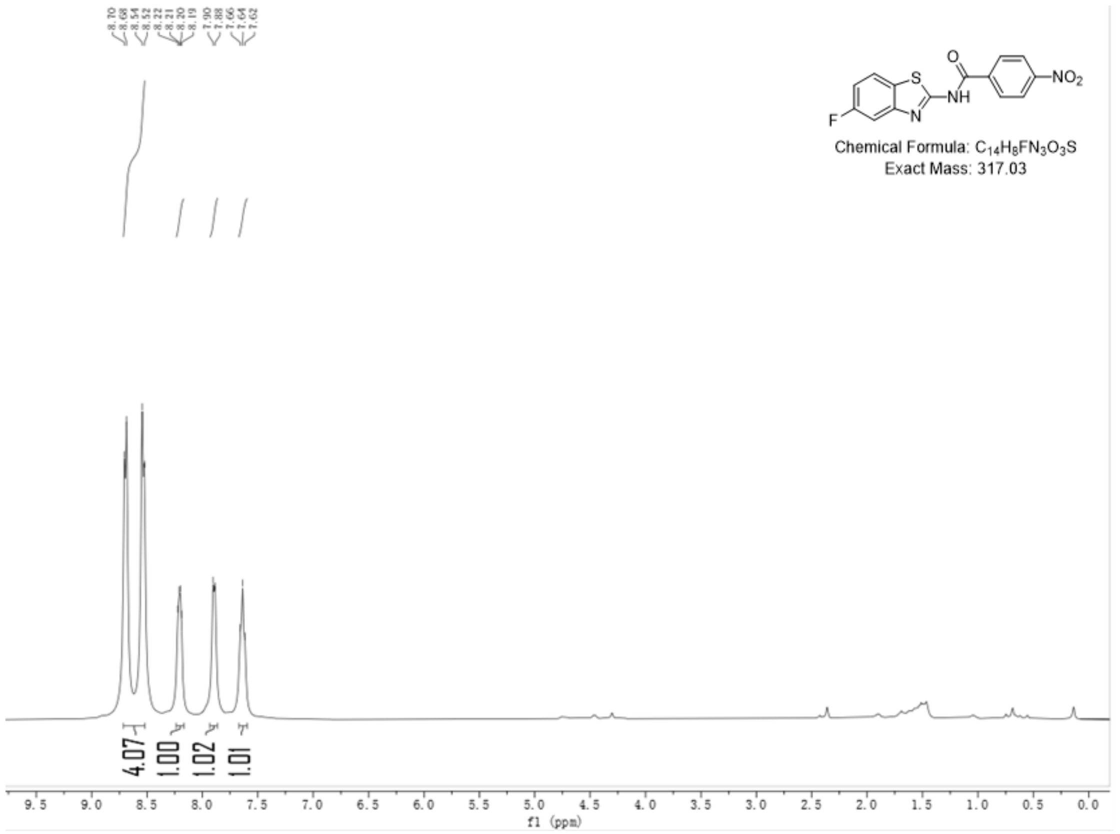

Supplementary Figure 78. ^{1}H NMR spectra of the compound **C4**.

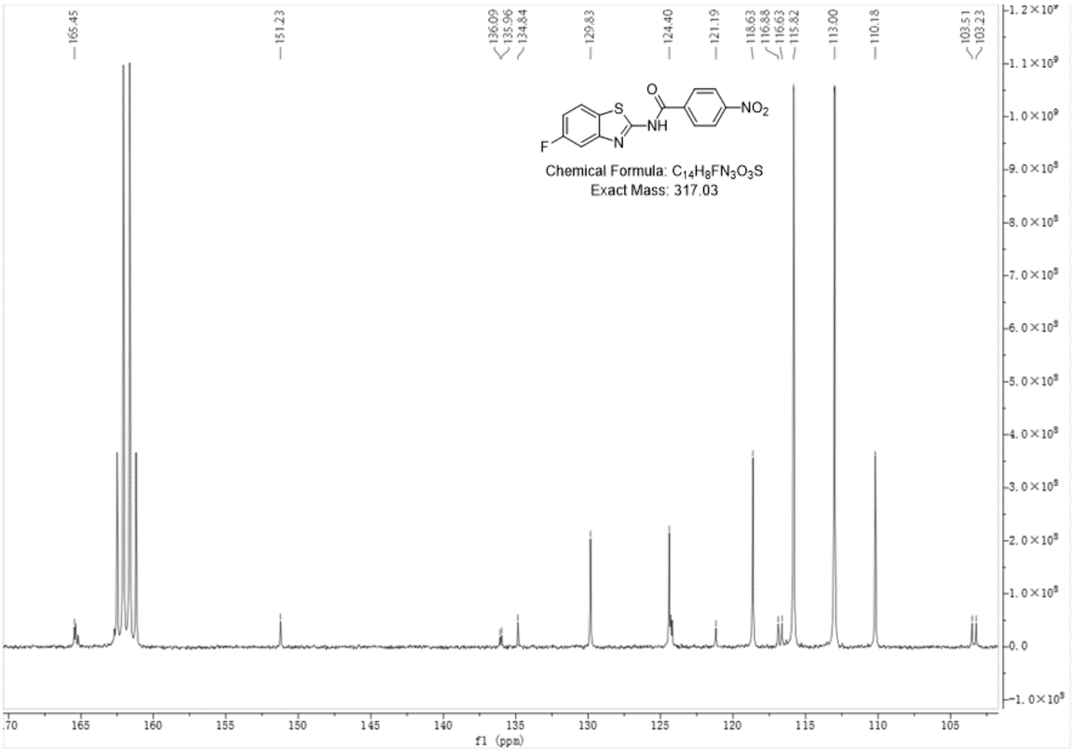

Supplementary Figure 79. ^{13}C NMR spectra of the compound **C4**.

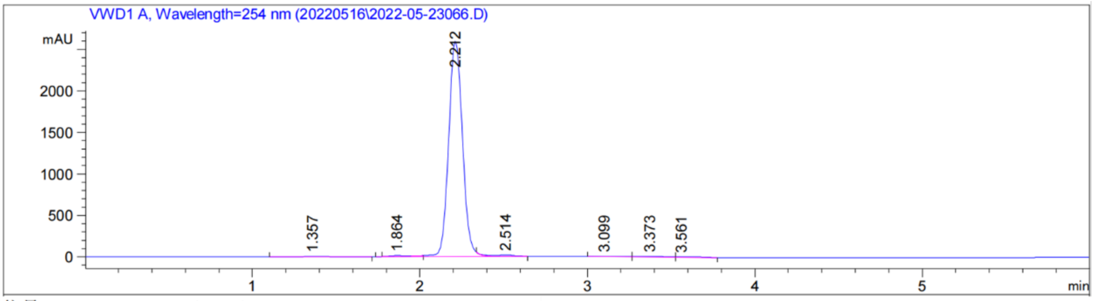

信号 1: VWD1 A, Wavelength=254 nm

峰 #	保留时间 [min]	类型	峰宽 [min]	峰面积 [mAU*s]	峰高 [mAU]	峰面积 %
1	1.357	BB	0.1771	25.90920	1.94924	0.1689
2	1.864	BV E	0.0943	63.63856	10.26669	0.4148
3	2.212	VV R	0.0890	1.48973e4	2595.33789	97.1042
4	2.514	VB E	0.1313	142.74597	15.52310	0.9305
5	3.099	BV	0.2258	35.39524	2.04590	0.2307
6	3.373	VV	0.1888	98.12762	7.03182	0.6396
7	3.561	VB	0.1577	78.43836	7.91134	0.5113
总量 :				1.53415e4	2640.06598	

Supplementary Figure 80. HPLC of the compound **C4.**

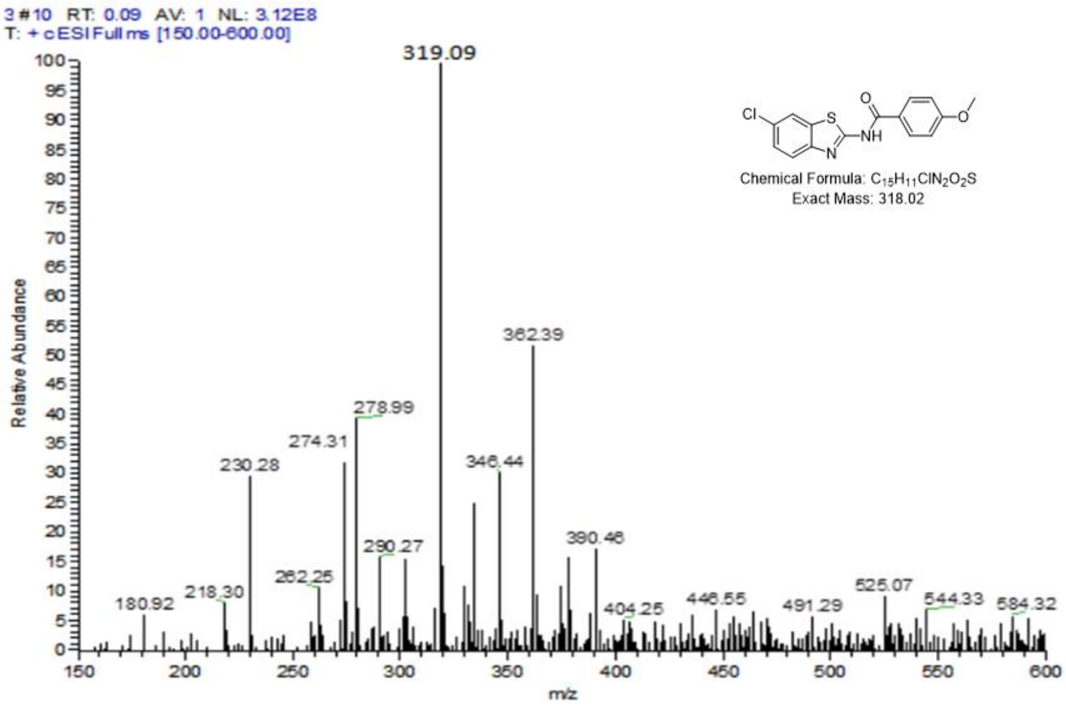

Supplementary Figure 81. Mass spectra of the compound **C5**.

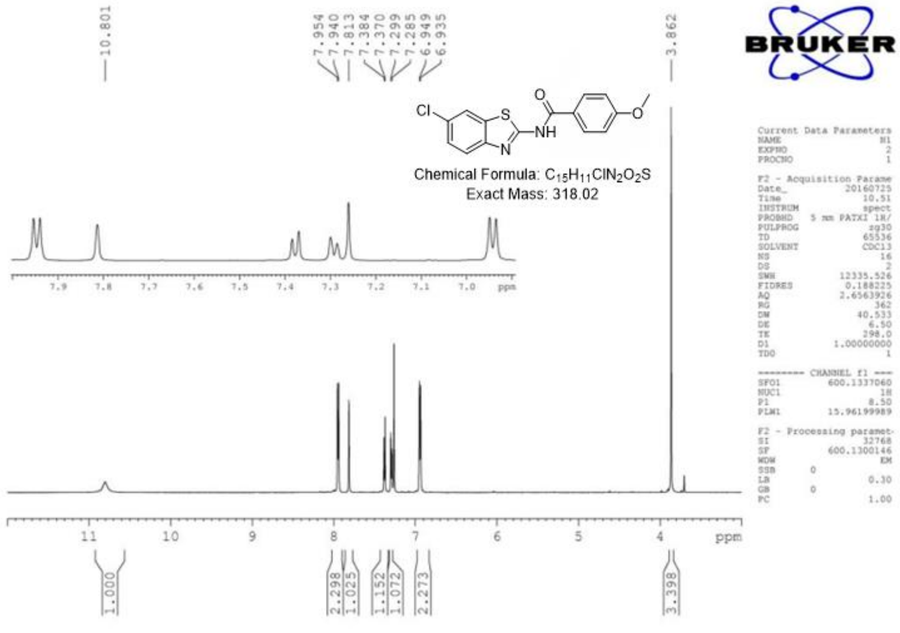

Supplementary Figure 82. ^{1}H NMR spectra of the compound **C5**.

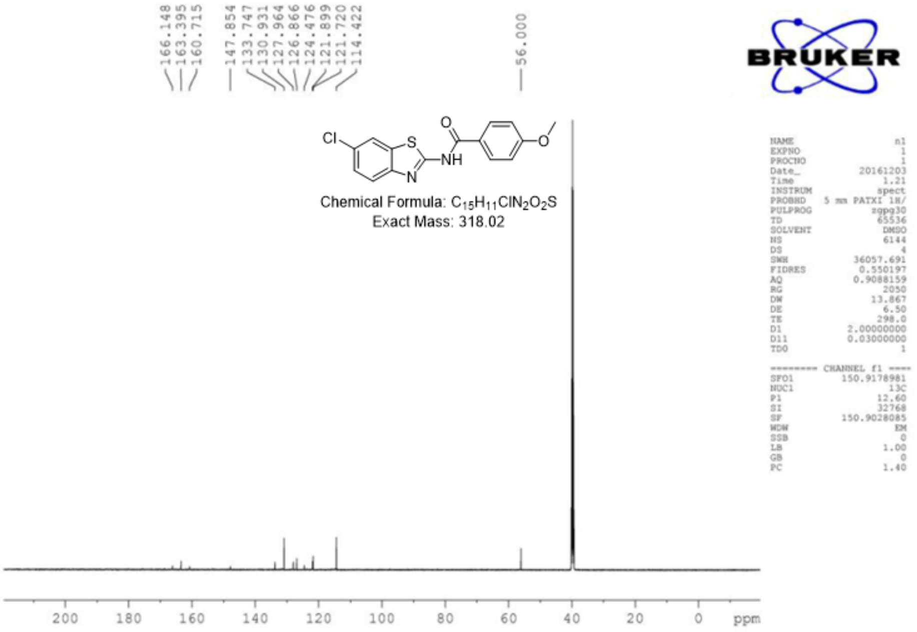

Supplementary Figure 83. ^{13}C NMR spectra of the compound **C5**.

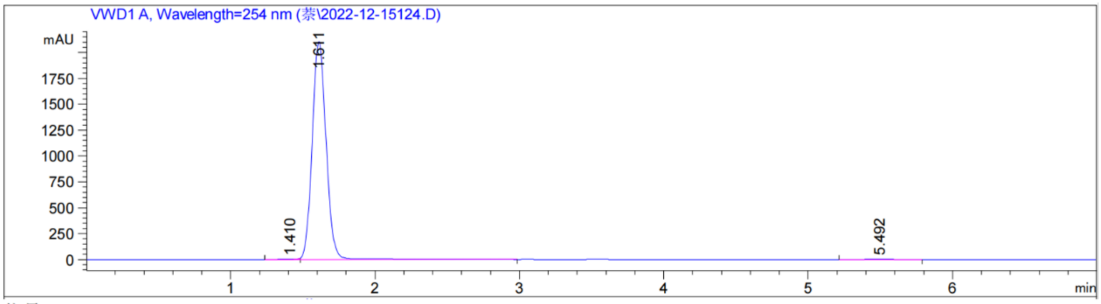

信号 1: VWD1 A, Wavelength=254 nm

峰 #	保留时间 [min]	类型	峰宽 [min]	峰面积 [mAU*s]	峰高 [mAU]	峰面积 %
1	1.410	BV E	0.0928	21.31263	3.51242	0.1499
2	1.611	VV R	0.1045	1.41701e4	2106.80054	99.6815
3	5.492	BB	0.1689	23.97013	2.20417	0.1686

总量 : 1.42154e4 2112.51712

Supplementary Figure 84. HPLC of the compound **C5.**

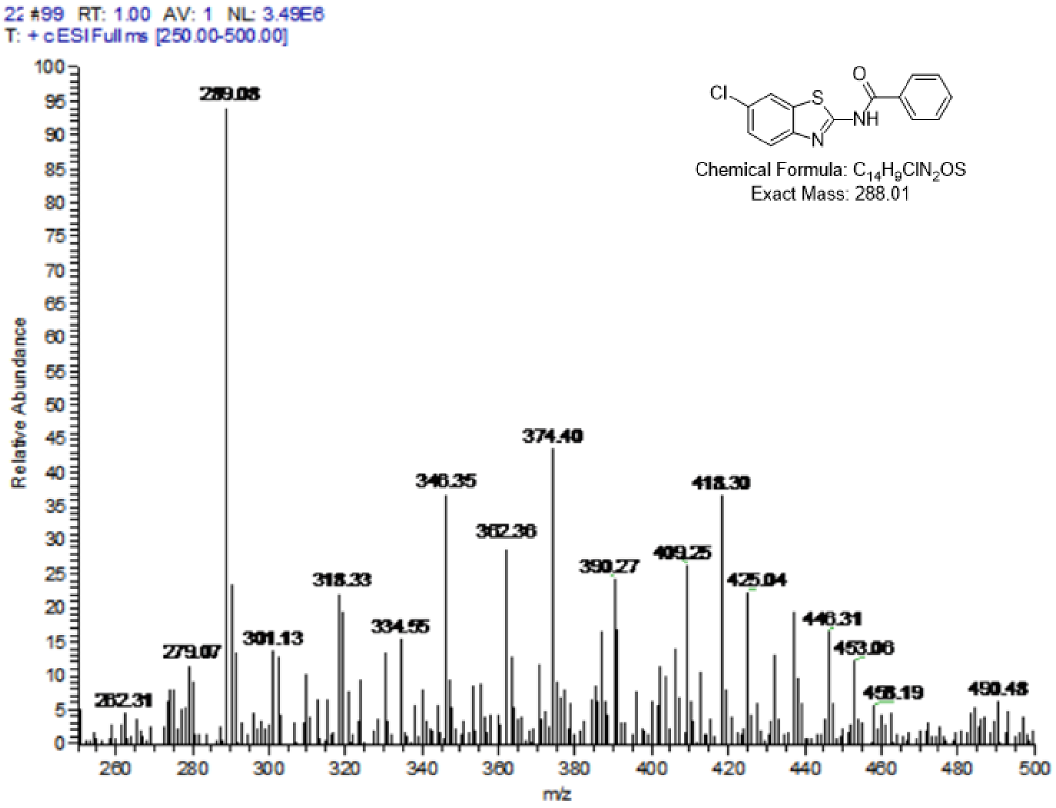

Supplementary Figure 85. Mass spectra of the compound **C6**.

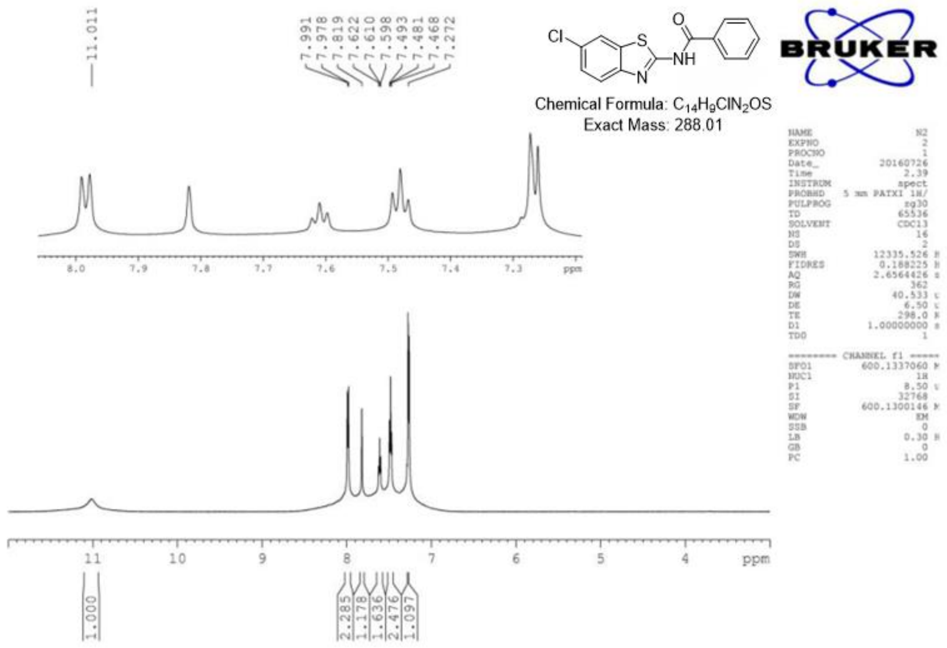

Supplementary Figure 86. ^{1}H NMR spectra of the compound **C6**.

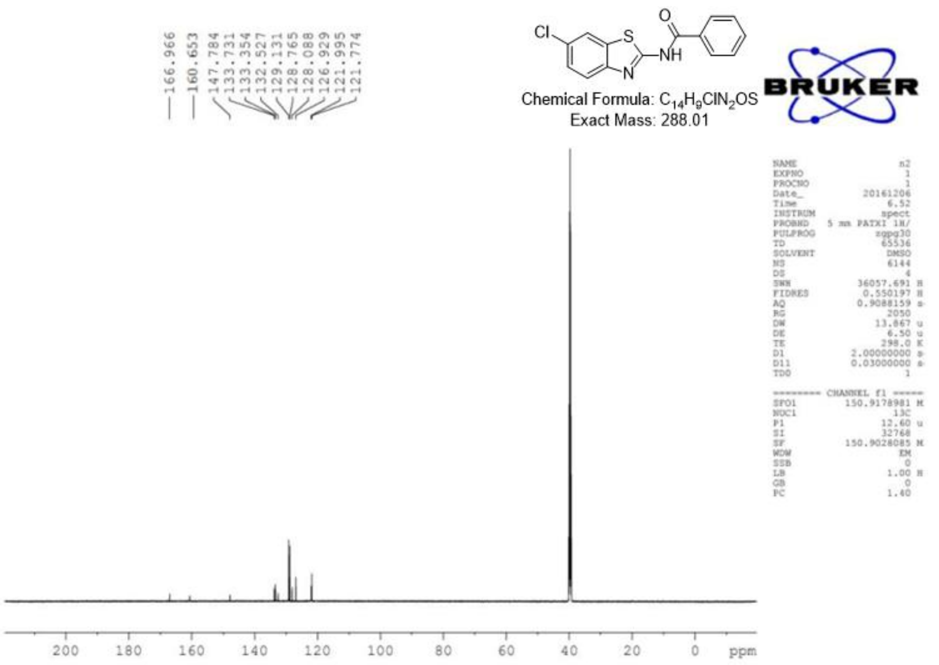

Supplementary Figure 87. ^{13}C NMR spectra of the compound **C6**.

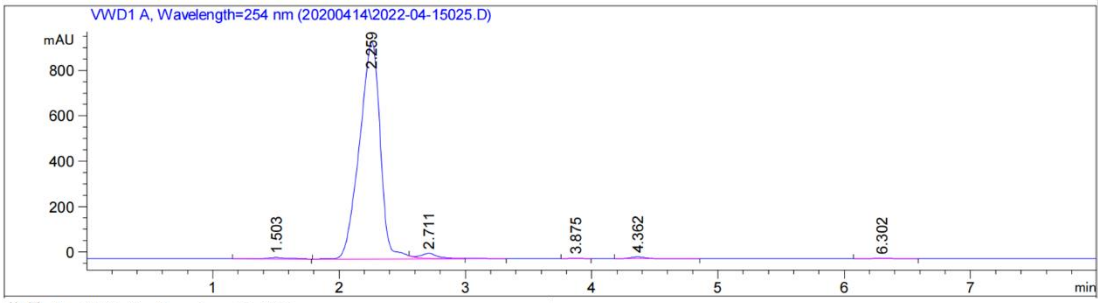

信号 1: VWD1 A, Wavelength=254 nm

峰 #	保留时间 [min]	类型	峰宽 [min]	峰面积 [mAU*s]	峰高 [mAU]	峰面积 %
1	1.503	BB	0.1666	77.96305	6.46919	0.6857
2	2.259	BV R	0.1719	1.09537e4	954.67163	96.3354
3	2.711	VV E	0.1360	210.89938	22.37305	1.8548
4	3.875	VV	0.1247	20.52112	2.52842	0.1805
5	4.362	BV R	0.1477	73.78130	7.70802	0.6489
6	6.302	BB	0.1501	33.51290	3.48674	0.2947
总量 :				1.13704e4	997.23705	

Supplementary Figure 88. HPLC of the compound **C6.**

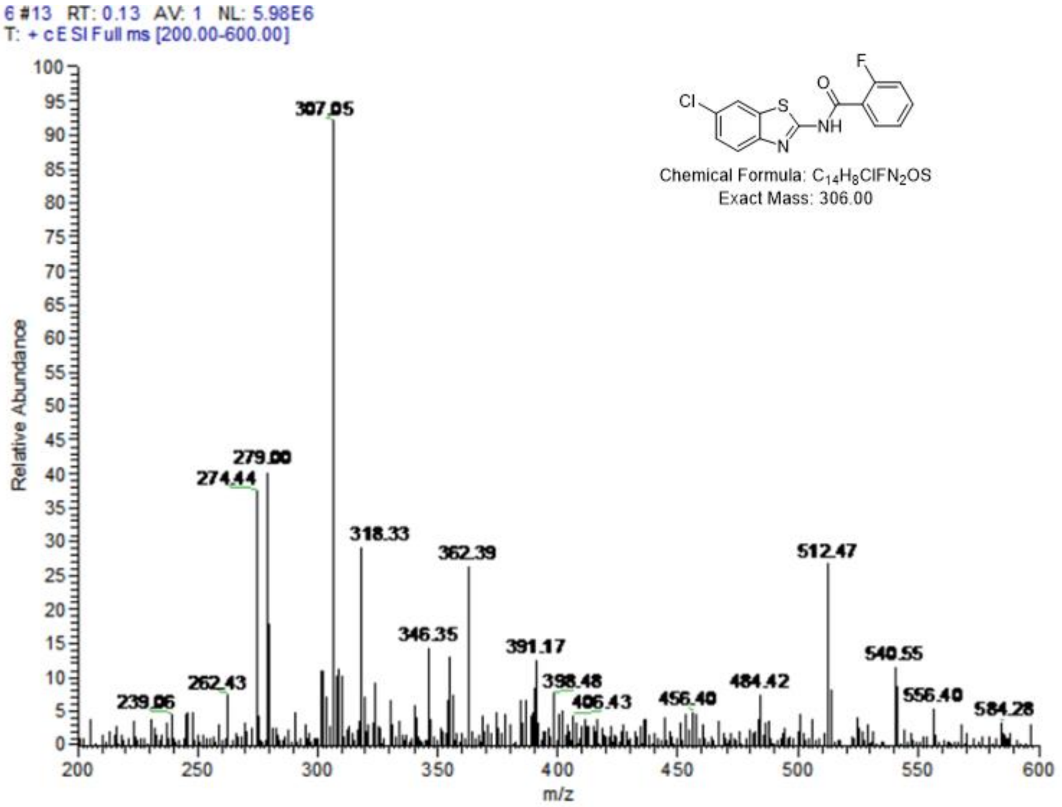

Supplementary Figure 89. Mass spectra of the compound **C7**.

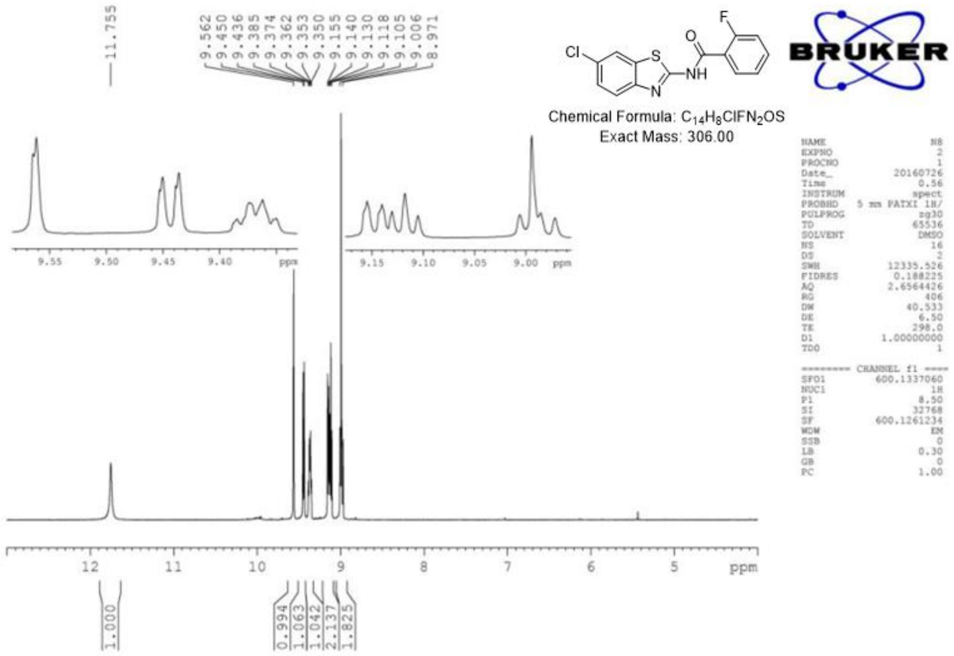

Supplementary Figure 90. ¹H NMR spectra of the compound **C7**.

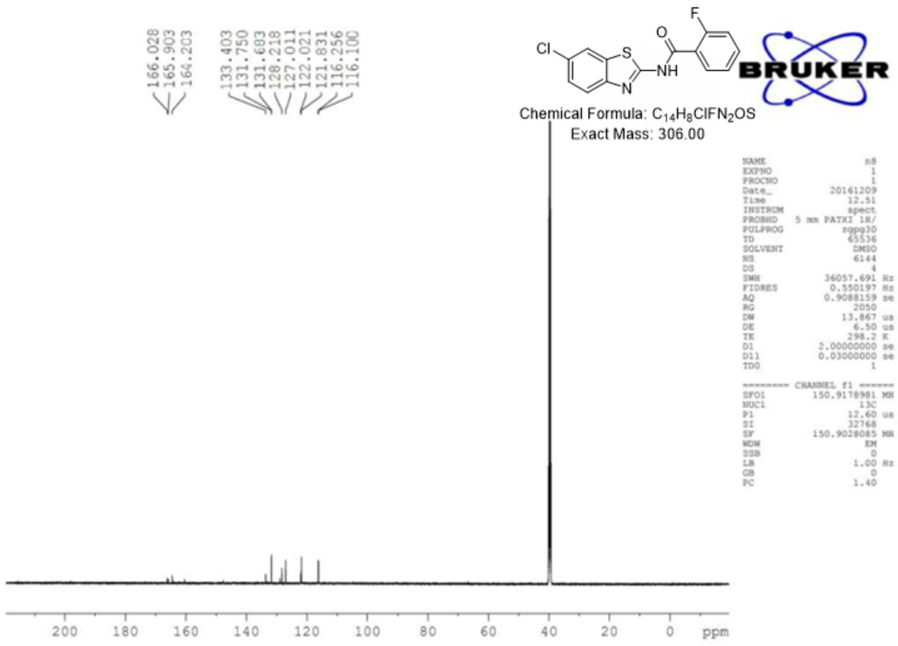

Supplementary Figure 91. ^{13}C NMR spectra of the compound **C7**.

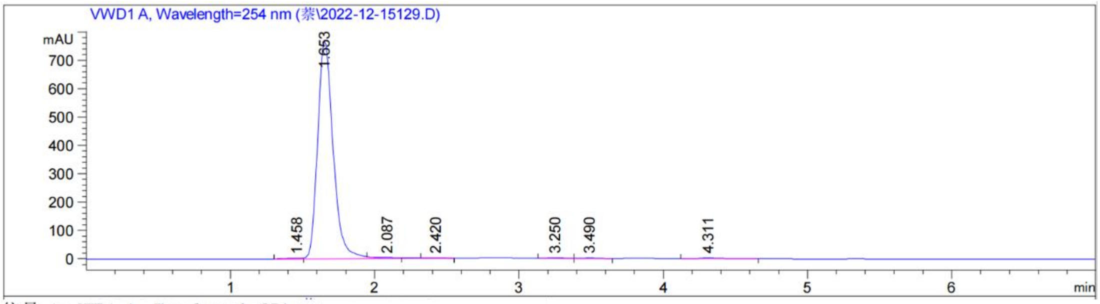

信号 1: VWD1 A, Wavelength=254 nm

峰 #	保留时间 [min]	类型	峰宽 [min]	峰面积 [mAU*s]	峰高 [mAU]	峰面积 %
1	1.458	BV E	0.1156	13.01736	1.77477	0.2219
2	1.653	VV R	0.1155	5738.22119	765.23108	97.8285
3	2.087	VV E	0.1112	23.48521	3.00516	0.4004
4	2.420	VB E	0.1134	17.29548	2.30812	0.2949
5	3.250	BV	0.1107	21.47311	3.03067	0.3661
6	3.490	VB	0.1216	16.24281	2.11556	0.2769
7	4.311	BB	0.1328	35.85584	3.91755	0.6113
总量 :				5865.59099	781.38291	

Supplementary Figure 92. HPLC of the compound **C7.**

1.4 Spectrums of the compounds D1-D2

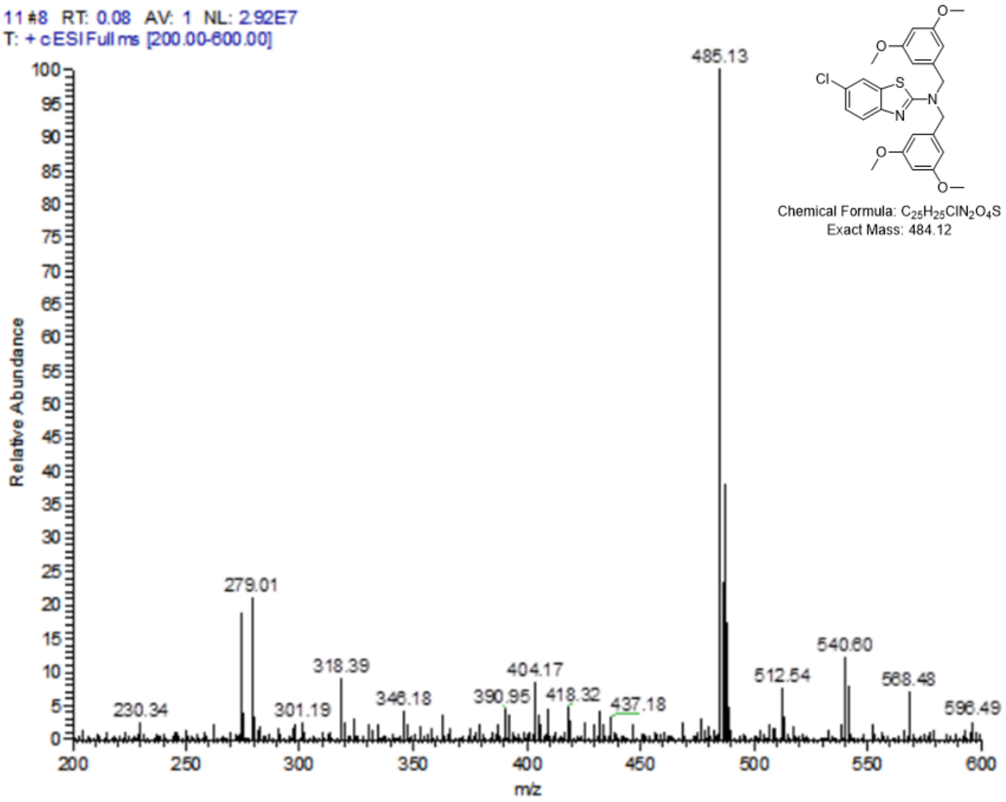

Supplementary Figure 93. Mass spectra of the compound **D1**.

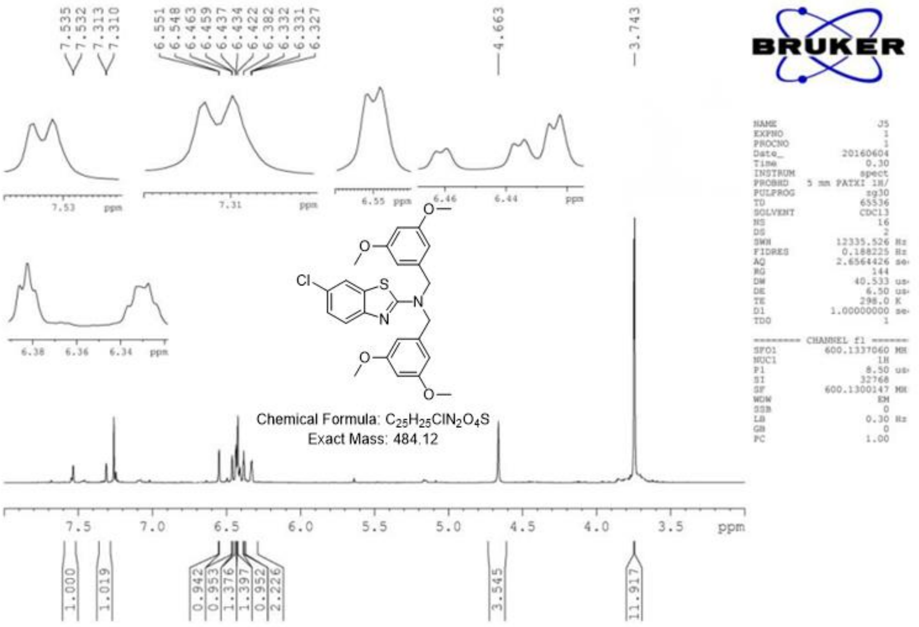

Supplementary Figure 94. ^{1}H NMR spectra of the compound **D1**.

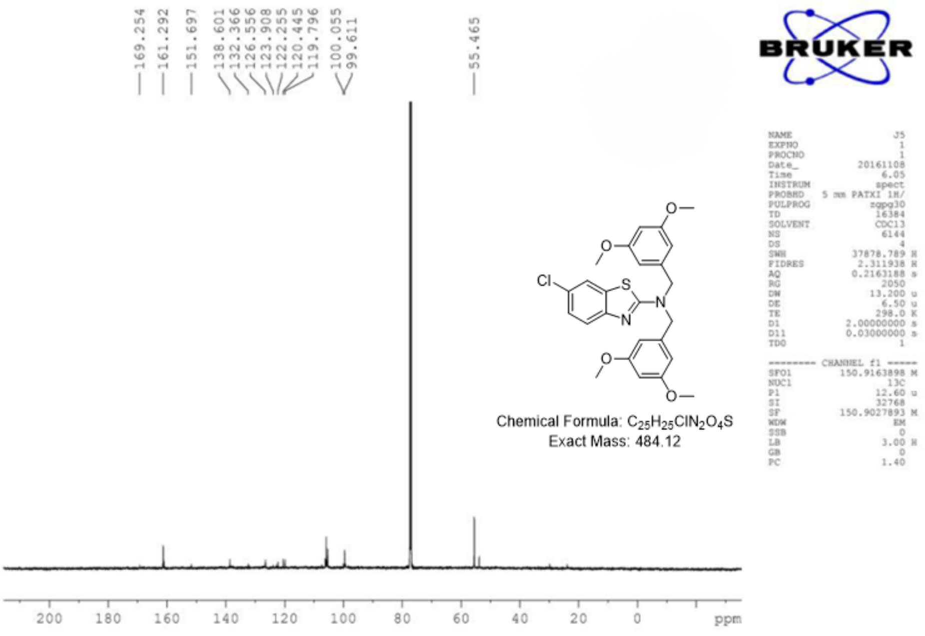

Supplementary Figure 95. ^{13}C NMR spectra of the compound **D1**.

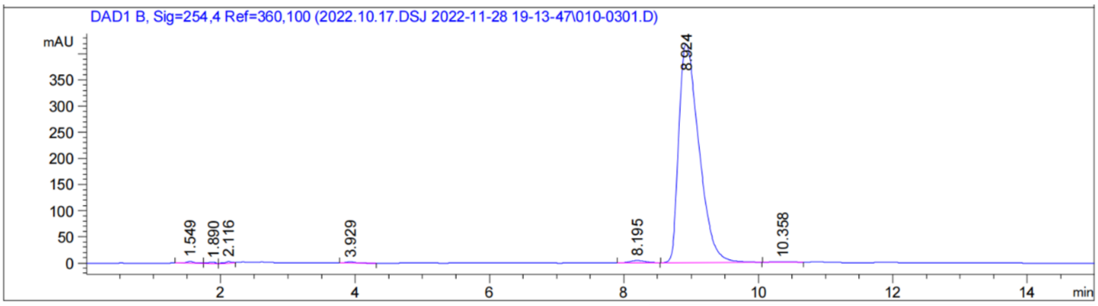

信号 2: DAD1 B, Sig=254,4 Ref=360,100

峰 #	保留时间 [min]	类型	峰宽 [min]	峰面积 [mAU*s]	峰高 [mAU]	峰面积 %
1	1.549	BV	0.1093	29.78510	3.72530	0.3358
2	1.890	VB	0.1165	18.89177	2.28359	0.2130
3	2.116	BV	0.0904	19.84792	3.03162	0.2238
4	3.929	VB	0.1410	19.39939	2.11596	0.2187
5	8.195	BB	0.2304	65.57861	4.33762	0.7394
6	8.924	BB	0.3249	8697.54688	417.76434	98.0618
7	10.358	BB	0.2791	18.40443	1.02681	0.2075
总量 :				8869.45409	434.28524	

Supplementary Figure 96. HPLC of the compound **D1.**

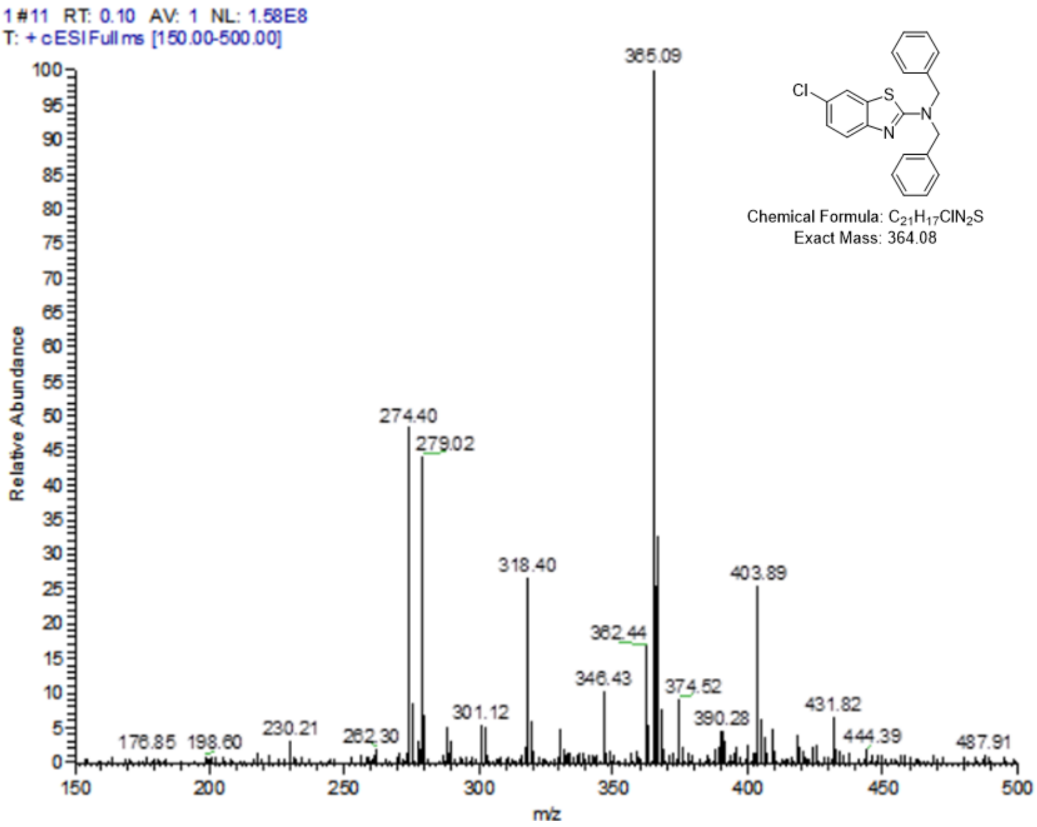

Supplementary Figure 97. Mass spectra of the compound **D2**.

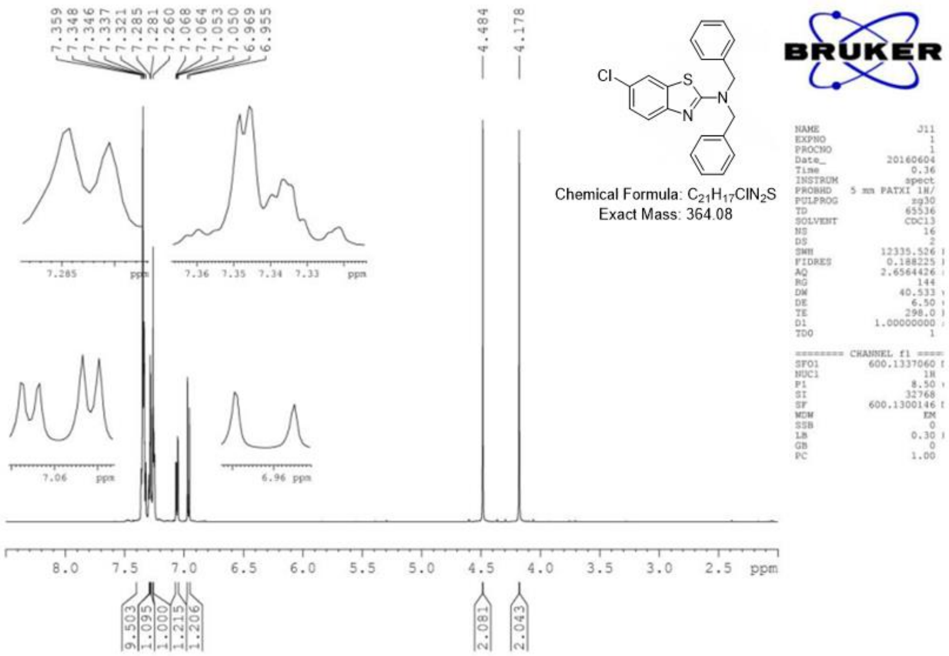

Supplementary Figure 98. ^{1}H NMR spectra of the compound **D2**.

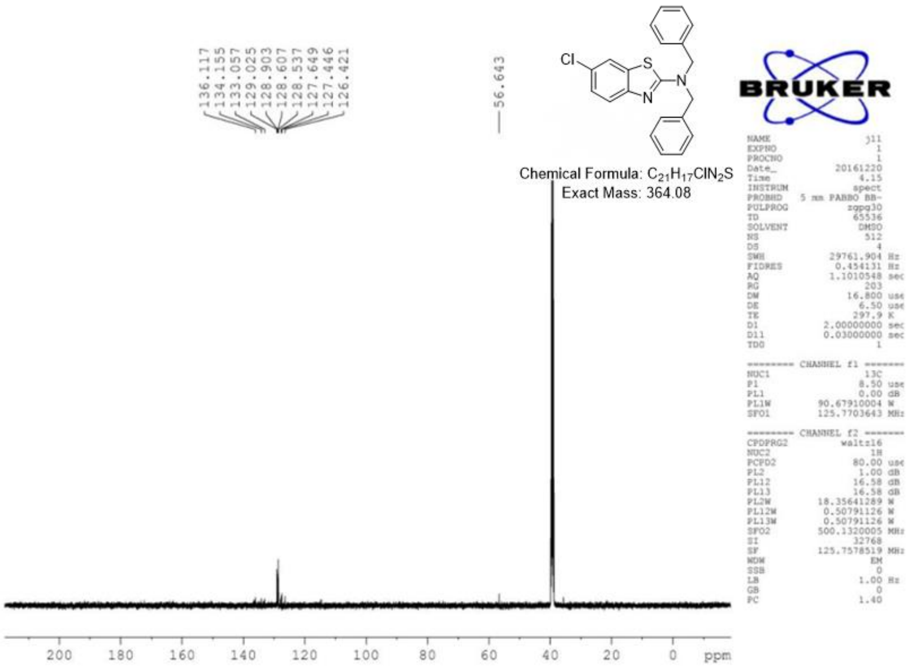

Supplementary Figure 99. ^{13}C NMR spectra of the compound **D2**.

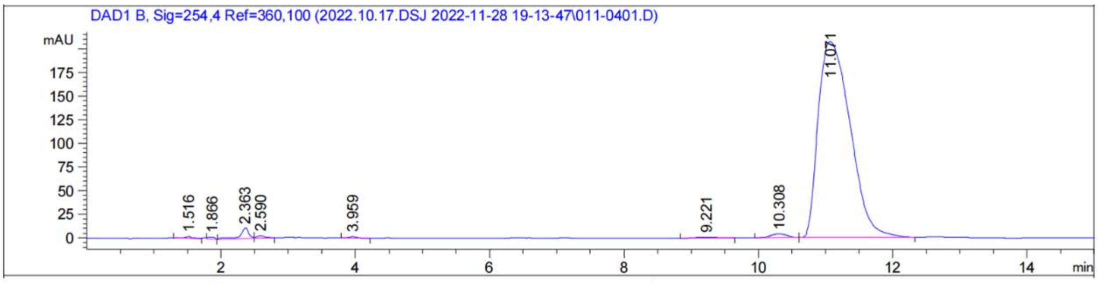

信号 2: DAD1 B, Sig=254,4 Ref=360,100

峰 #	保留时间 [min]	类型	峰宽 [min]	峰面积 [mAU*s]	峰高 [mAU]	峰面积 %
1	1.516	BV	0.1032	19.14886	2.56305	0.2689
2	1.866	VB	0.0986	15.31214	2.27143	0.2150
3	2.363	BV	0.1316	102.65117	11.33987	1.4416
4	2.590	VB	0.1243	24.39968	2.83809	0.3427
5	3.959	BB	0.1141	15.17353	2.05623	0.2131
6	9.221	BB	0.2680	20.92028	1.15149	0.2938
7	10.308	BV	0.2902	78.85789	4.33559	1.1074
8	11.071	VB	0.5483	6844.39893	206.73447	96.1176
总量 :				7120.86247	233.29021	

Supplementary Figure 100. HPLC of the compound **D2.**
